# Supplementary material for: Genome-wide association study of thyroid-stimulating hormone highlights new genes, pathways and associations with thyroid disease
Source: Nat Commun. 2023 Oct 23;14:6713. doi: 10.1038/s41467-023-42284-5 (PMC10593800; doi:10.1038/s41467-023-42284-5)
Supplement: Supplementary file 1 — Supplementary Information [file 41467_2023_42284_MOESM1_ESM.pdf]

Supplementary Data and Figures for the manuscript entitled "Genome-wide association study of thyroid-stimulating hormone highlights new genes, pathways and association with thyroid disease

Contents

Supplementary Note 2

Supplementary Methods 4

TSH phenotype definition 4

Supplementary Figures 4

Supplementary Figure 1 4

Supplementary Figure 2 5

Supplementary Figure 3 25

Supplementary Figure 4 26

Supplementary Figure 5 38

Supplementary Figure 6 39

Supplementary Figure 7 40

Supplementary Figure 8 41

Supplementary Figure 9 42

Supplementary Figure 10 43

Supplementary Data Legends 44

Supplementary Note

Consortium Authors

Estonian Biobank Research Team

Andres Metspalu[1,2], Lili Milani[1], Reedik Mägi[1], Mari Nelis[1], Georgi Hudjashov[1], Tõnu Esko[1].

Genes & Health Research Team

In alphabetical order by surname: Shaheen Akhtar[3], Mohammad Anwar[4], Elena Arciero[3], Omar Asgar[5], Samina Ashraf[6], Saeed Bidi[7], Gerome Breen[8], James Broster[7], Raymond Chung[8], David Collier[7], Charles J Curtis[8], Shabana Chaudhary[7], Megan Clinch[7], Grainne Colligan[4], Panos Deloukas[7], Ceri Durham[4], Faiza Durrani[7], Fabiola Eto[7], Sarah Finer[7], Joseph Gafton[7], Ana Angel Garcia[7], Chris Griffiths[7], Joanne Harvey[7], Teng Heng[3], Sam Hodgson[7], Qin Qin Huang[3], Matt Hurles[3], Karen A Hunt[7], Shapna Hussain[7], Kamrul Islam[7], Vivek Iyer[3], Ben Jacobs[7], Ahsan Khan[7], Cath Lavery[7], Sang Hyuck Lee[8], Robin Lerner[7], Daniel MacArthur[9], Daniel Malawsky[3], Hilary Martin[3], Dan Mason[10], Rohini Mathur[7], Mohammed Bodrul Mazid[7], John McDermott[11], Caroline Morton[7], Bill Newman[11], Elizabeth Owor[7], Asma Qureshi[7], Samiha Rahman[7], Shwetha Ramachandrappa[8], Mehru Reza[7], Jessry Russell[7], Nishat Safa[7], Miriam Samuel[7], Michael Simpson[8], John Solly[7], Marie Spreckley[7], Daniel Stow[7], Michael Taylor[7], Richard C Trembath[8], Karen Tricker[7], Nasir Uddin[7], David A van Heel[7], Klaudia Walter[3], Caroline Winckley[12], Suzanne Wood[7], John Wright[13], Julia Zollner[14].

Consortia affiliations

1. Estonian Genome Center, Institute of Genomics, University of Tartu, Tartu, Estonia
2. Institute of Cell and Molecular Biology, University of Tartu, Tartu, Estonia
3. Wellcome Sanger Institute, London, UK
4. Social Action for Health, London, UK
5. Manchester University NHS Trust, Manchester, UK
6. Bradford Teaching Hospitals, Bradford, UK
7. Queen Mary University of London, London, UK
8. King's College London, London, UK
9. Garvan Institute of Medical Research, Darlinghurst, Australia
10. Born in Bradford, Bradford, UK
11. University of Manchester, Manchester, UK
12. NIHR Clinical Research Clinical trials, Manchester, UK
13. Bradford Institute for Health Research, Bradford, UK
14. University College London, London, UK

Cohort Descriptions

Estonian Biobank (EstBB)

All EstBB participants have been genotyped at the Core Genotyping Lab of the Institute of Genomics, University of Tartu, using Illumina Global Screening Array v3.0_EST. Samples were genotyped and PLINK format files were created using Illumina GenomeStudio v2.0.4. Individuals were excluded from the analysis if their call-rate was <95% or if sex defined based on heterozygosity of X chromosome did not match sex in the phenotype data. Before imputation, variants were filtered by call-rate <95%, Hardy-Weinberg $P<1\times10^{-4}$ (autosomal variants only), and minor allele frequency <1%. Genotyped variant positions were in build 37 and were lifted over to build 38 using Picard. Phasing was performed using the Beagle v5.4 software. Imputation was performed with Beagle v5.4 software (beagle.22Jul22.46e.jar) and default settings. The dataset was split into batches of 5,000. A population-specific reference panel consisting of 2,695 whole-genome sequencing (WGS) samples was utilised for imputation and standard Beagle hg38 recombination maps were used. Based on principal component analysis, samples that were not of European ancestry were removed. Duplicates and monozygous twin were identified using KING 2.2.7, and one sample was removed out of every pair of duplicates.

The TSH concentrations were queried with LOINC code 3016-3 ("Thyrotropin in Serum or Plasma", n=63,326), with values below 0.4mU/L and above 4.0 mU/L being excluded from the analysis. We used earliest possible measurement from each participant and analyses were restricted to individuals with European ancestry.

Association analyses in Estonian Biobank were carried out for all variants with an imputation quality score >0.4 under an additive model as implemented in REGENIE v3.0.3 with standard quantitative trait settings. Linear regression was carried out on inverse normalised TSH concentration values and adjusted for current age, age^2, sex and 10 principal components as covariates, analysing only variants with a minimum minor allele count of 2.

Extended Cohort for E-Health, Environment and DNA (EXCEED)

Samples were genotyped using the UK Biobank Axiom array. Prior to imputation, individuals were excluded if their call rate was <97% or if genetic sex and phenotypic sex did not match; variants were excluded if the call rate was <95%, Hardy-Weinberg $P<1\times10^{-6}$ or minor allele frequency <1%. Imputation was conducted using the TOPMed Imputation Server (phasing: Eagle v2.4, imputation: Minimac4, reference panel: TOPMed r2).

TSH measurements were captured from primary care electronic healthcare records, taking the earliest measurement where more than one was available for an individual. Association analyses in EXCEED were conducted for all variants with an imputation quality score >0.5 and a minor allele frequency >0.1% under an additive genetic model adjusted for age, sex and the first 10 principal components of ancestry using PLINK 2.0. Overall, we tested 14,847,247 variants in EXCEED.

UK Biobank (UKB)

Genotyping and imputation for UK Biobank samples are described in detail in the following source: Bycroft C, Freeman C, Petkova D, et al. The UK Biobank resource with deep phenotyping and genomic data. Nature. 2018 Oct;562(7726):203-209. doi: 10.1038/s41586-018-0579-z

TSH measurements were captured from primary care electronic healthcare records, taking the earliest measurement where more than one was available for an individual. Association analyses in UK Biobank were conducted for all variants with an imputation quality score >0.5 and a minor allele count >20 under an additive genetic model adjusted for age, genotyping array, sex and the first 10 principal components of ancestry using PLINK 2.0. Overall, we tested 53,096,898 variants in UK Biobank.

Zhou et al

This study has been described in detail elsewhere: Zhou, W., Brumpton, B., Kabil, O. et al. GWAS of thyroid stimulating hormone highlights pleiotropic effects and inverse association with thyroid cancer. Nat Commun 11, 3981 (2020). https://doi.org/10.1038/s41467-020-17718-z

In the HUNT study, TSH was measured using DELFIA hTSH Ultra from Wallac Oy (Turku, Finland) in HUNT2 and a chemiluminescent microparticle immunoassay on an Architect ci8200 from Abbott (Abbott Ireland, Longford, Ireland; and Abbott Laboratories, Abbott Park, IL) in HUNT3. Where an individual had TSH measured in HUNT2 and HUNT3, the measurement recorded in HUNT2 was used for association testing. In the Michigan Genomics Initiative, TSH was captured from electronic healthcare records. Where an individual had more than one TSH measurement available, the average TSH level was used for association testing. The ThyroidOmics consortium consists of a further 22 independent cohorts which have been described in detail elsewhere (Teumer, A., Chaker, L., Groeneweg, S. et al. Genome-wide analyses identify a role for SLC17A4 and AADAT in thyroid hormone regulation. Nat Commun 9, 4455 (2018). https://doi.org/10.1038/s41467-018-06356-1). In brief, TSH was derived from serum or plasma samples.

Overall, 22.4 million variants in up to 119,715 individuals of European ancestry were analysed. Our analysis used publicly available summary statistics.

Genes & Health

This study has been described in detail elsewhere: Sarah Finer, Hilary C Martin, Ahsan Khan, et al. Cohort Profile: East London Genes & Health (ELGH), a community-based population genomics and health study in British Bangladeshi and British Pakistani people, International Journal of Epidemiology, Volume 49, Issue 1, February 2020, Pages 20–21i, https://doi.org/10.1093/ije/dyz174

TSH was captured from primary care electronic healthcare records after mapping the Read version 2/3 codes we used in EXCEED and UK Biobank to the SNOMED coding framework. Where an individual had more than one measurement, the earliest measurement was used for association testing.

One hundred and seventy-seven (177) of the 260 sentinel variants from our stage 1 analysis were analysed in 33,171 individuals of South Asian ancestry.

Supplementary Methods

TSH phenotype definition

We investigated the impact of having restricted the TSH phenotype to 0.4-4.0 mU/L in our study by comparing the effect estimates of 256 TSH sentinel variants from the GWAS in UK Biobank (where TSH measures analysed were restricted to a reference range) to an analysis in which the TSH measures were unrestricted. The comparison of effect estimates is shown in Supplementary Figure 10. The effect estimates of the TSH sentinel variants tested were attenuated towards the null in the analysis of unrestricted TSH compared to our UK Biobank GWAS. Restricting the TSH measures to the above reference range may result, therefore, in a more powerful analysis.

Supplementary Figures

Supplementary Figure 1: Manhattan plot for the stage 1 meta-analysis

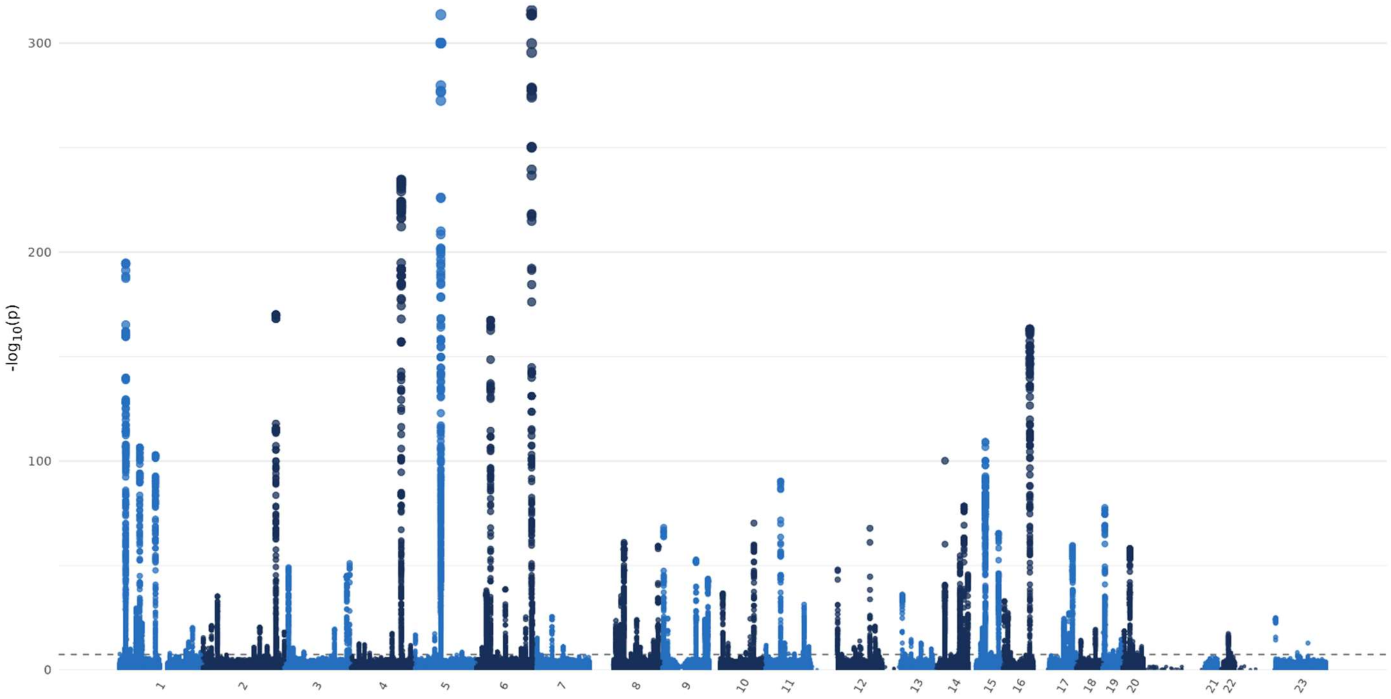

Supplementary Figure 2: Single variant PheWAS

a) rs115315671 (implicating *ADCY6*).

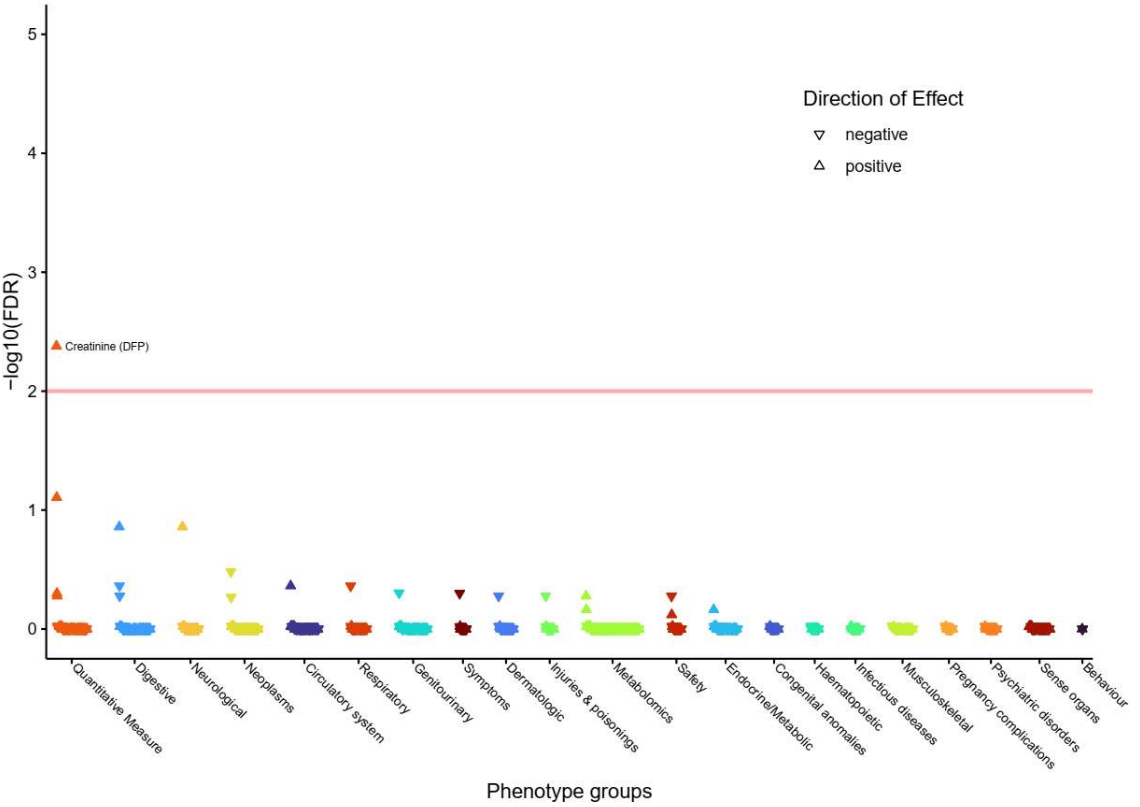

b) rs2494734 (implicating *AKT1* and *ZBTB42*).

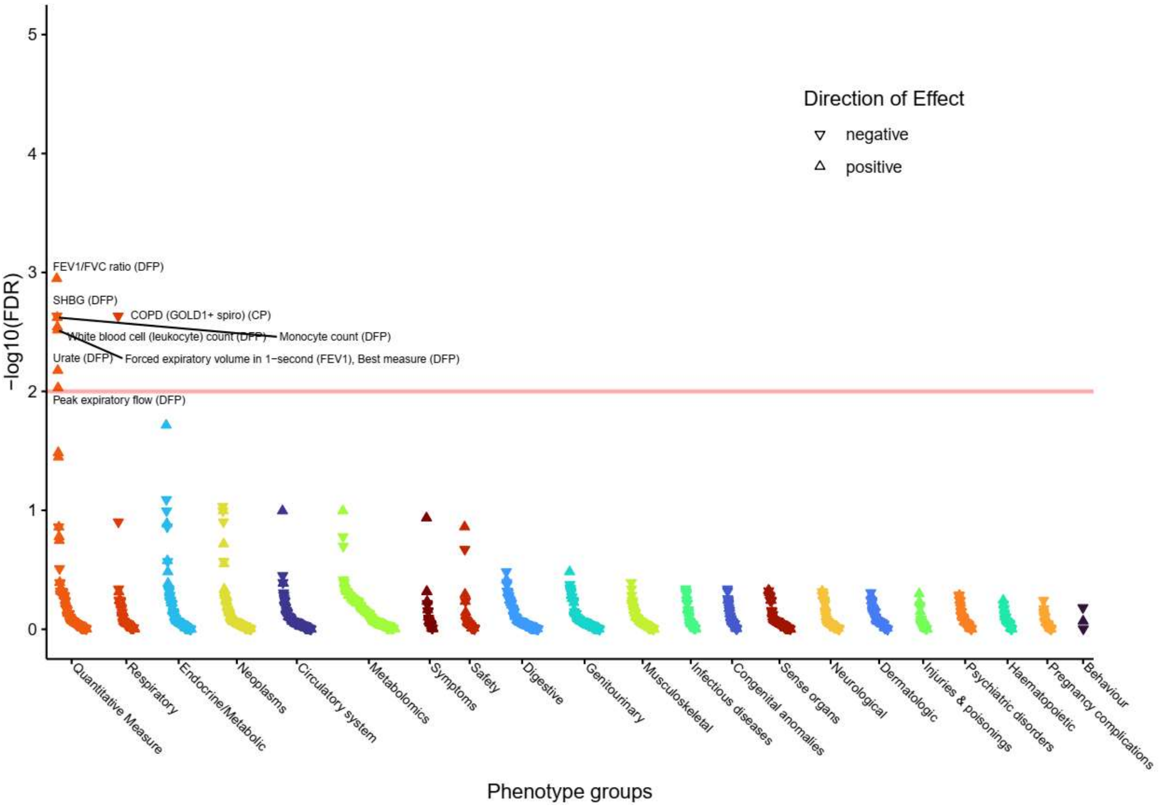

c) **rs17450274 (implicating *ANXA5*).**

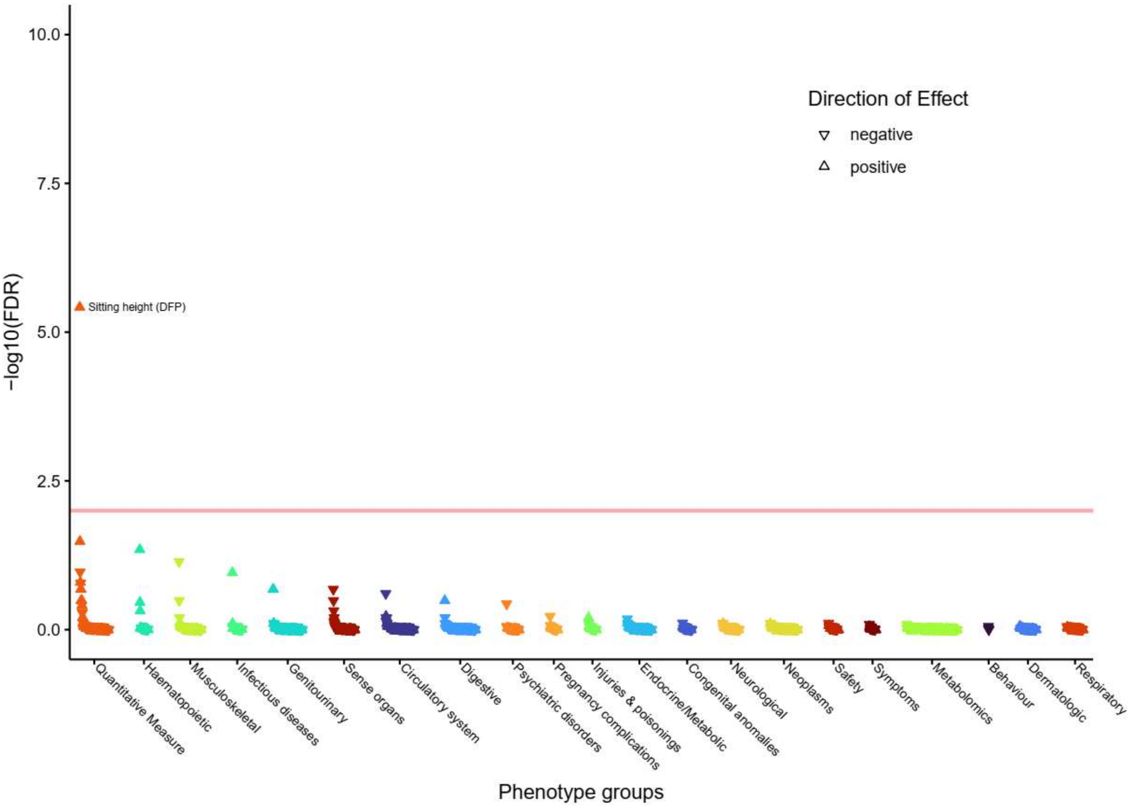

d) **rs11605461 (implicating *CADM1*).**

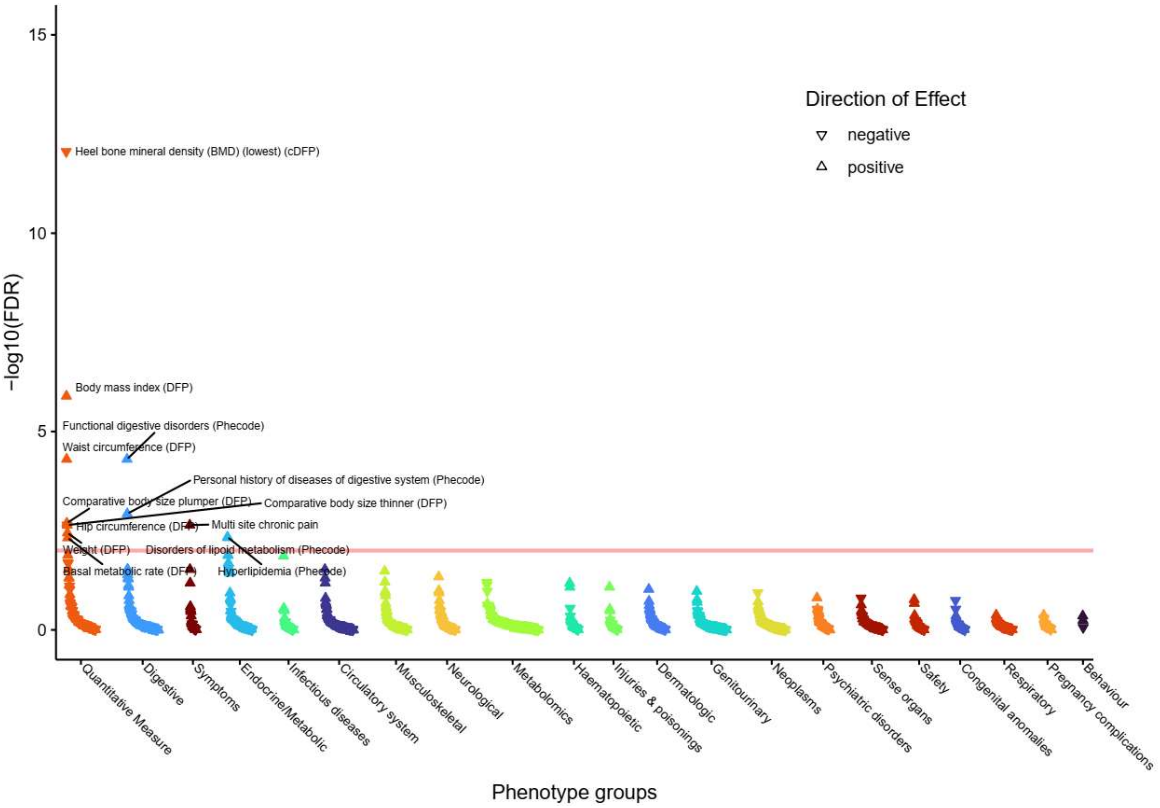

e) rs149363012 (implicating *CCDC77*, *WNK1* and *B4GALNT3*).

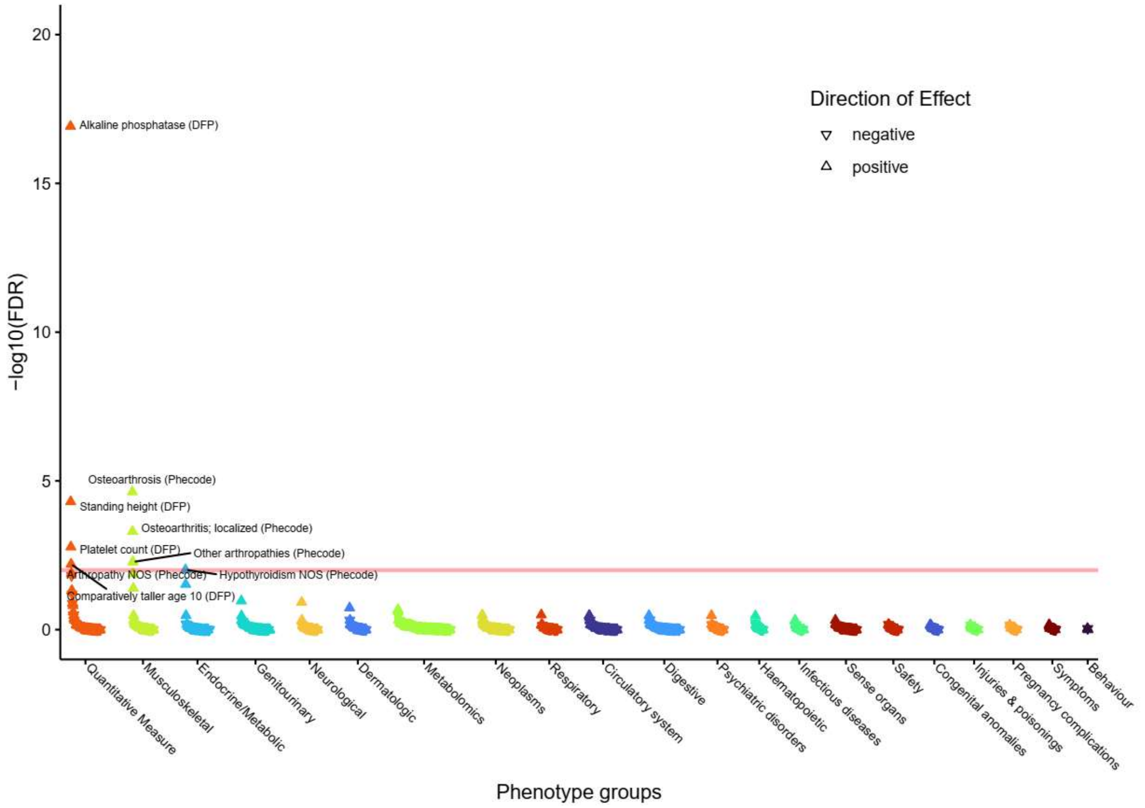

f) rs3124747 (implicating *DBH*, *C9orf96* and *STKLD1*).

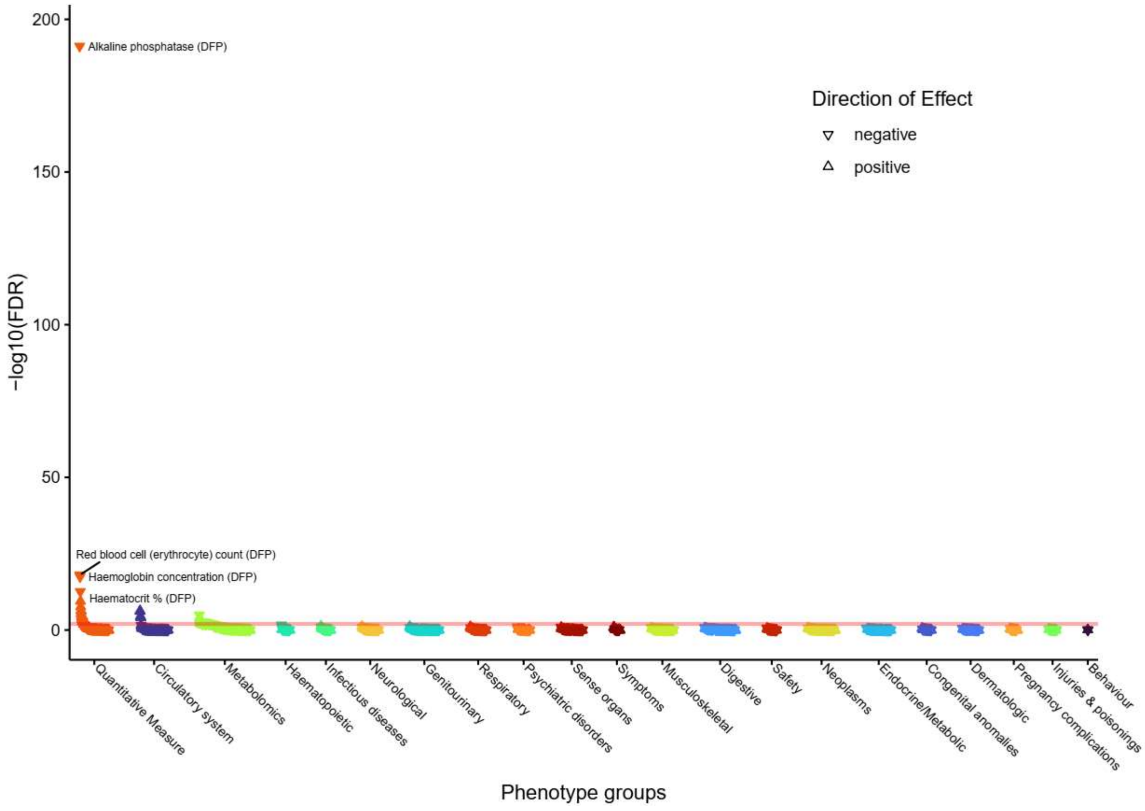

g) **rs139411099 (implicating *DIO2*).**

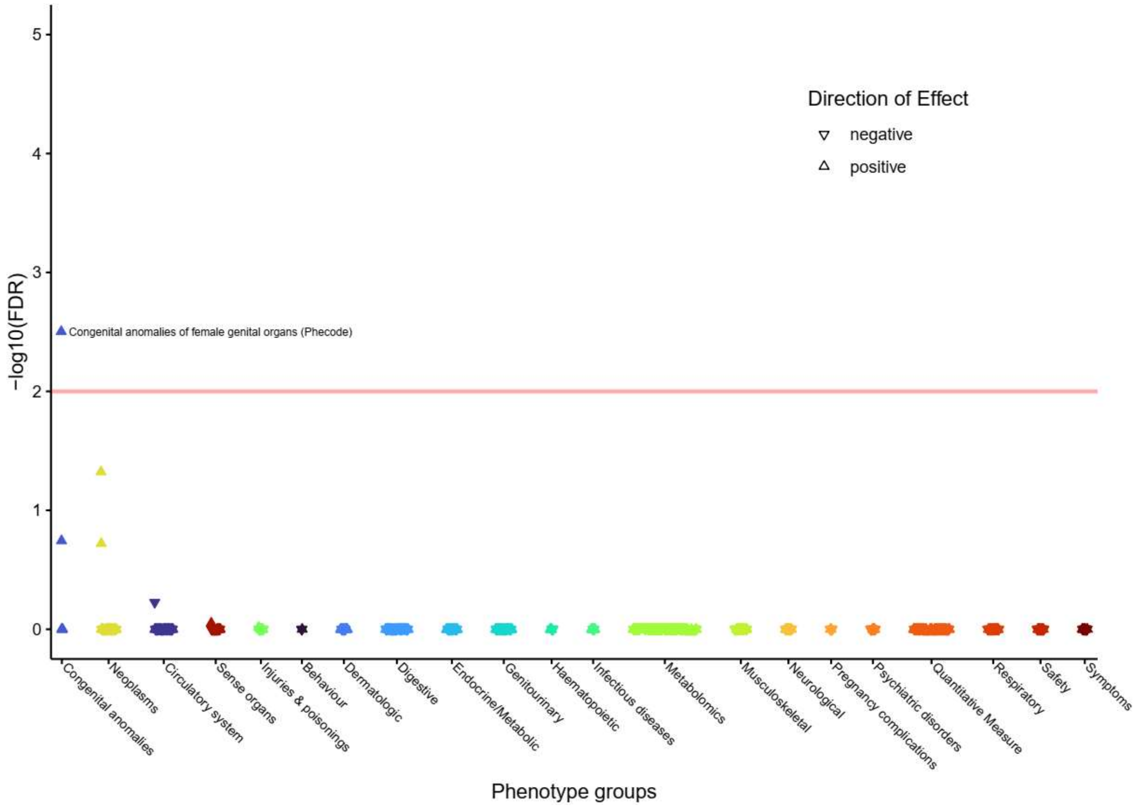

h) **rs2282192 (implicating *FOXE1*, *ANP32B* and *C9orf156*).**

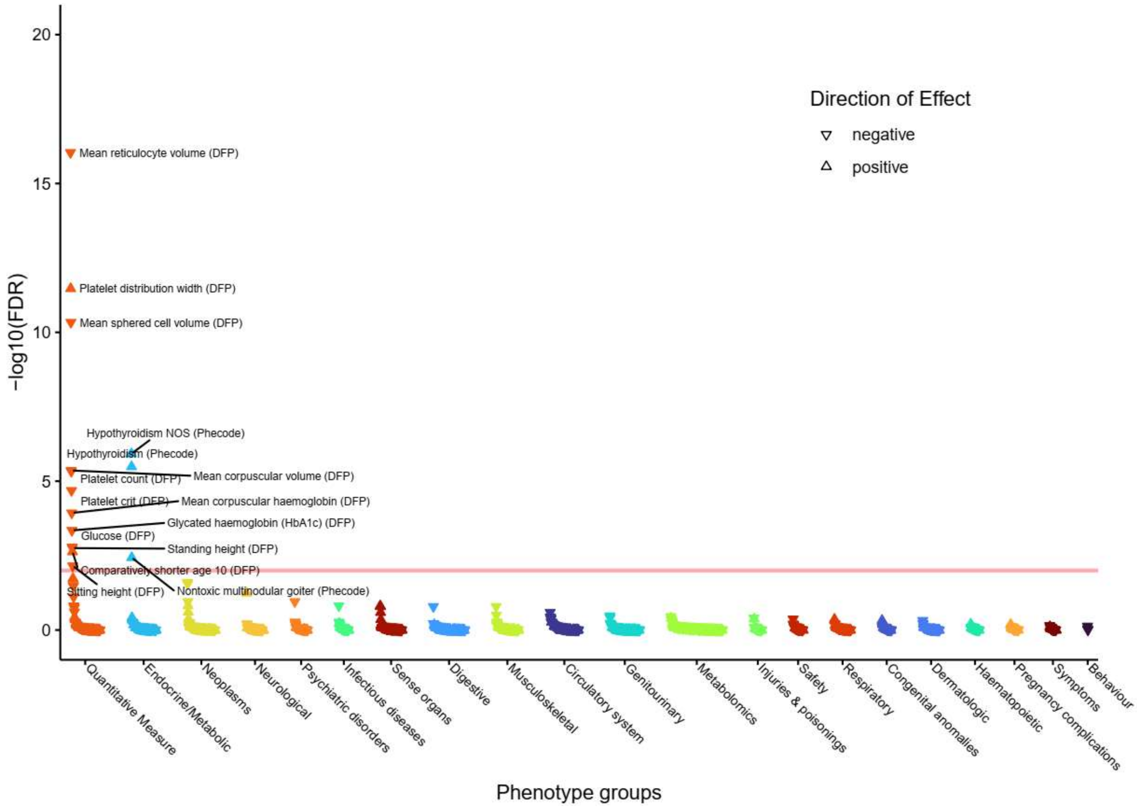

i) rs10814915 (implicating *GLIS3*).

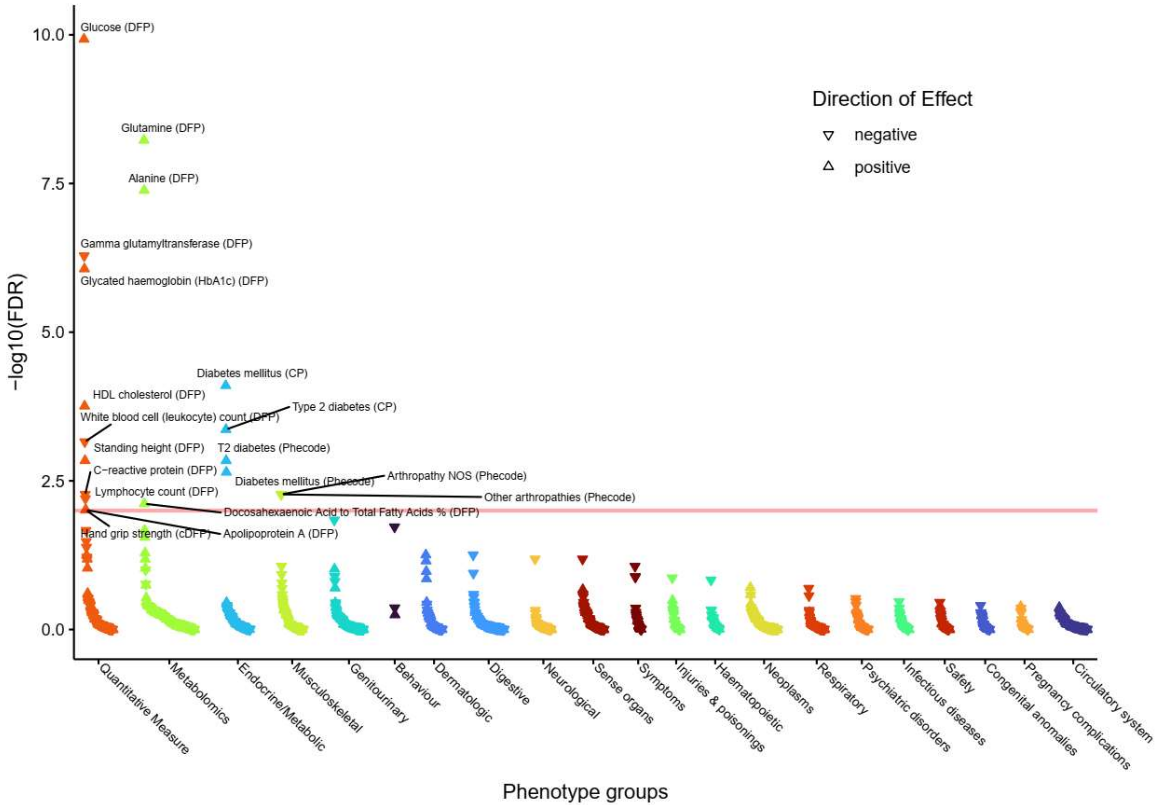

j) rs1861628 (implicating *IGFBP5*).

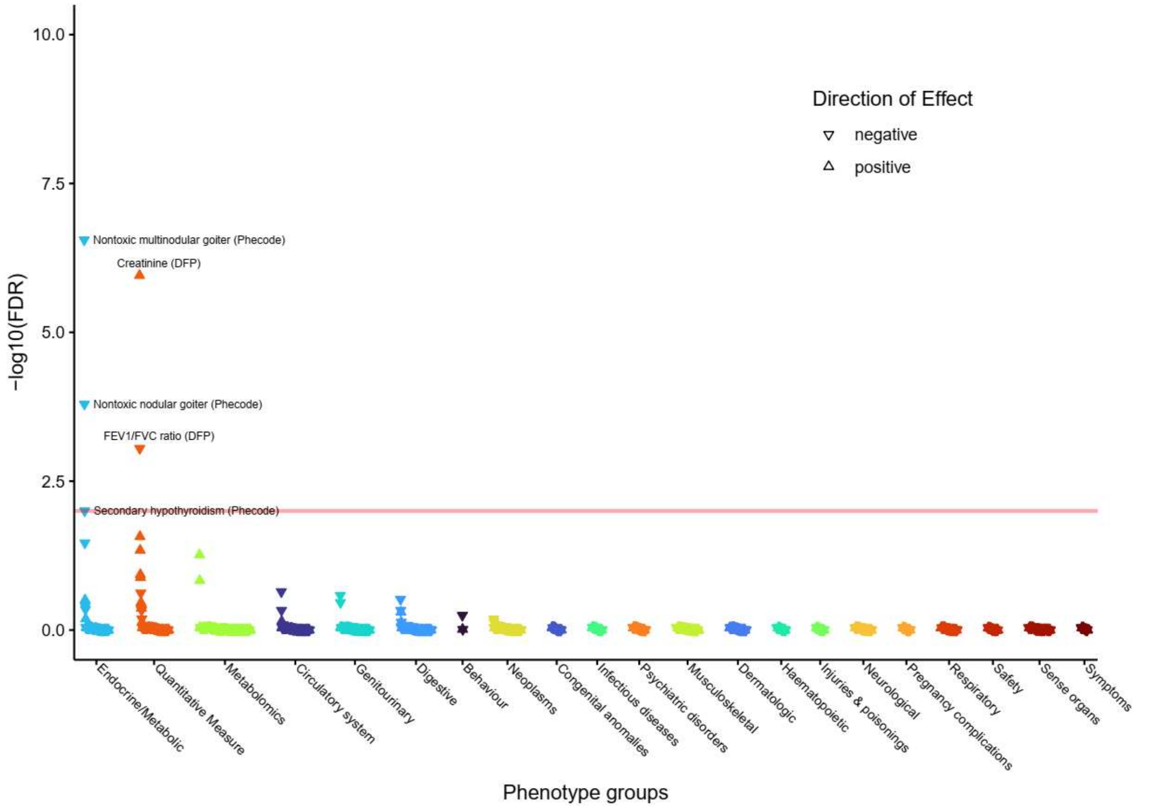

k) rs1861630 (implicating *IGFBP5*).

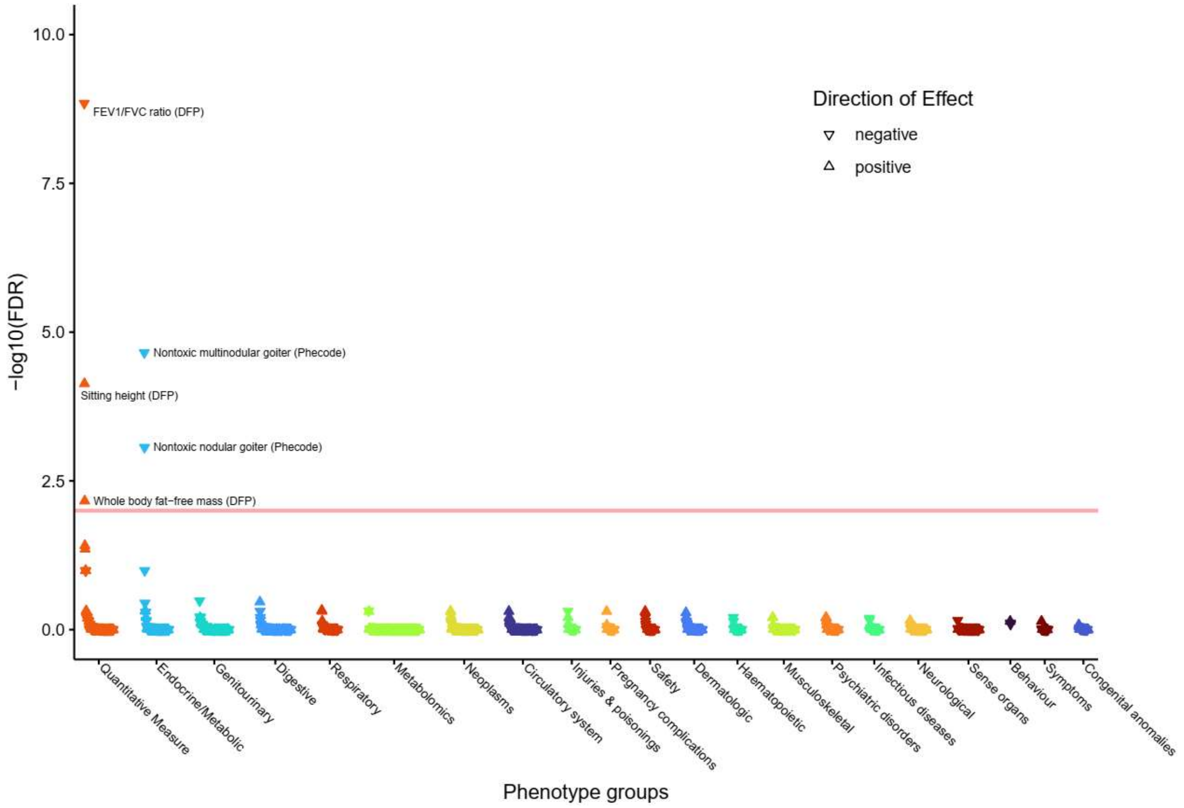

l) rs6711608 (implicating *IGFBP5*).

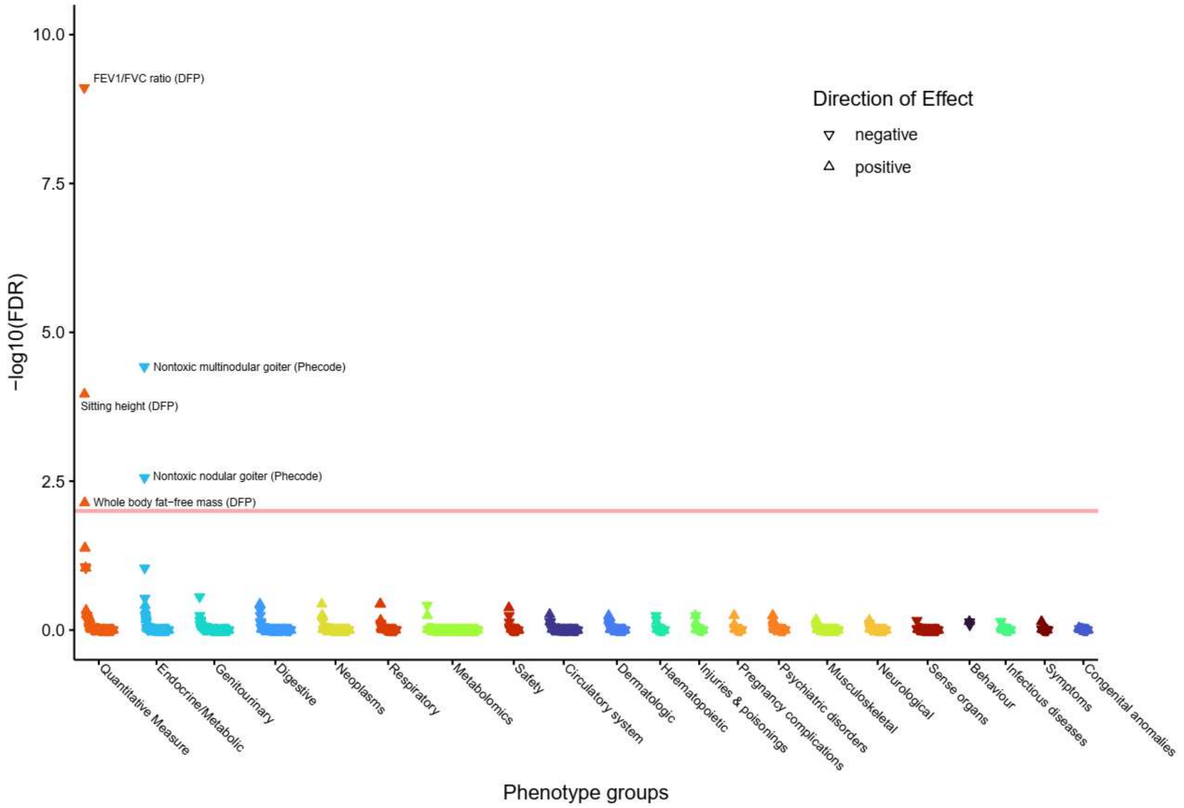

m) rs57866041 (implicating *IGFBP5*).

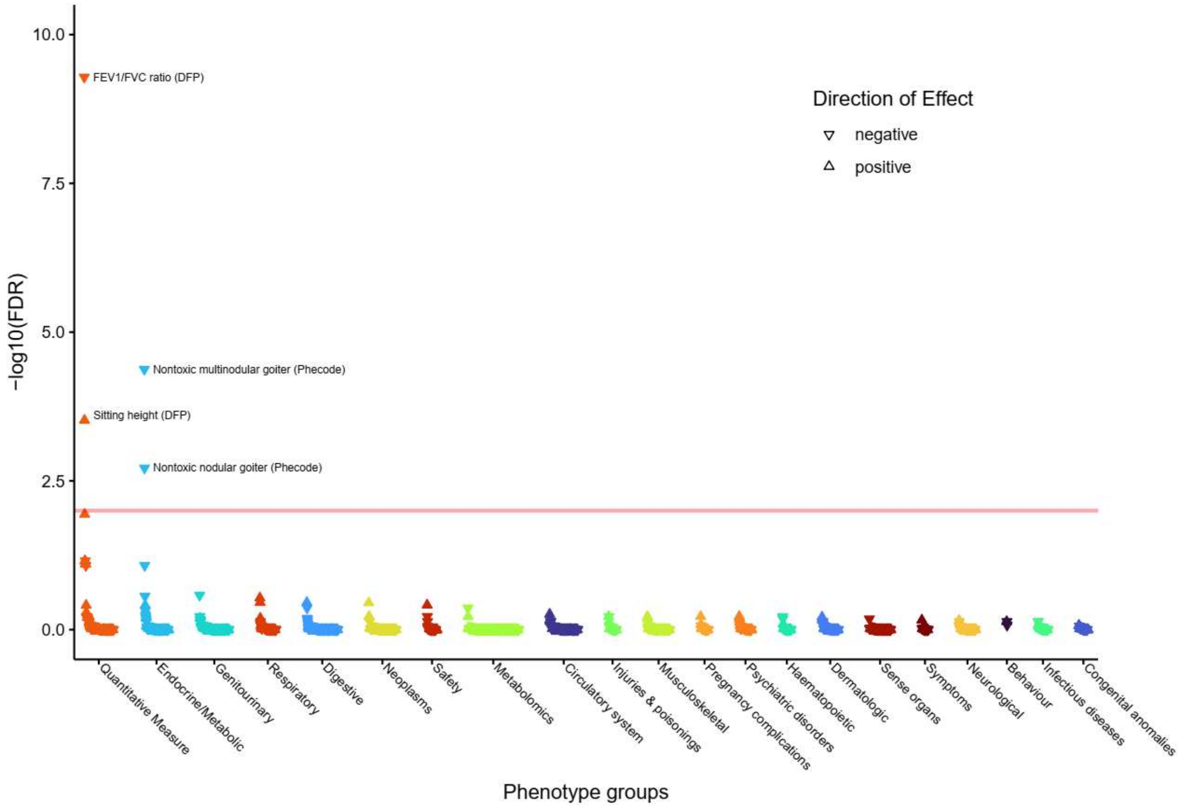

n) rs2229738 (implicating *IGHMBP2*, *CPT1A* and *SCD*).

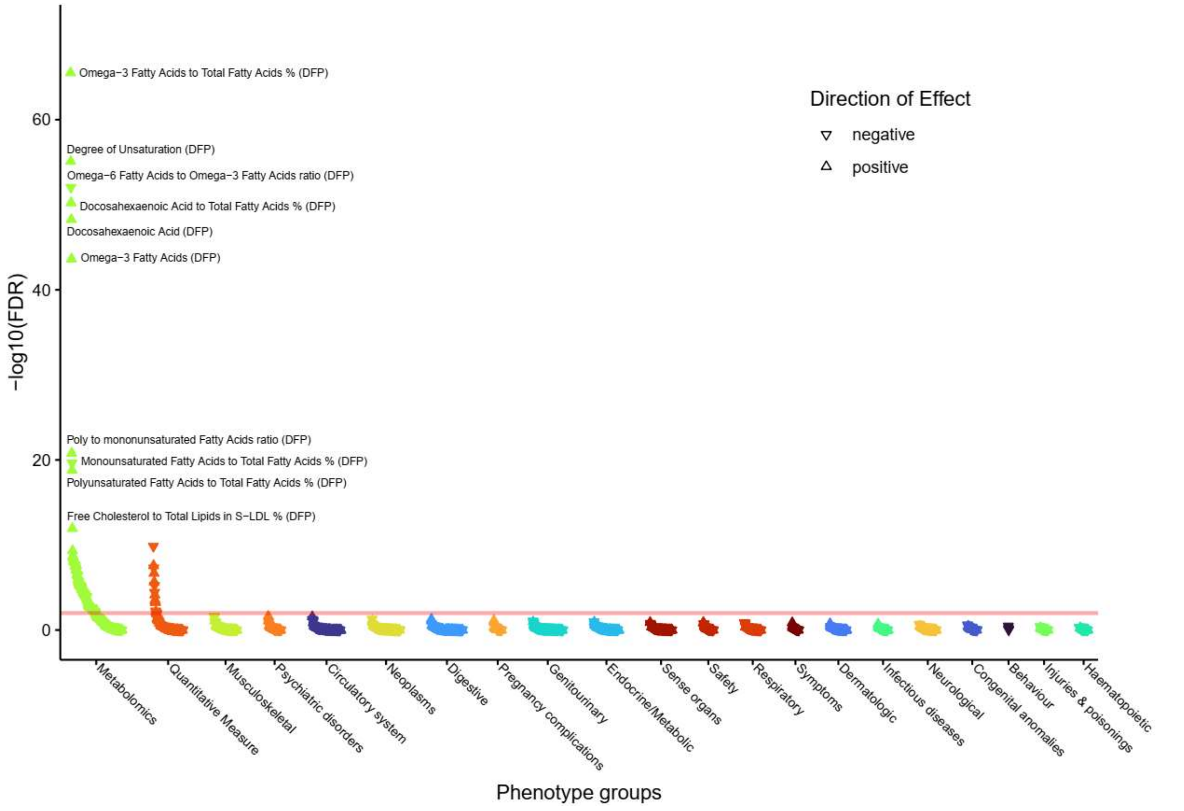

o) rs4804416 (implicating *INSR* and *ACTL9*).

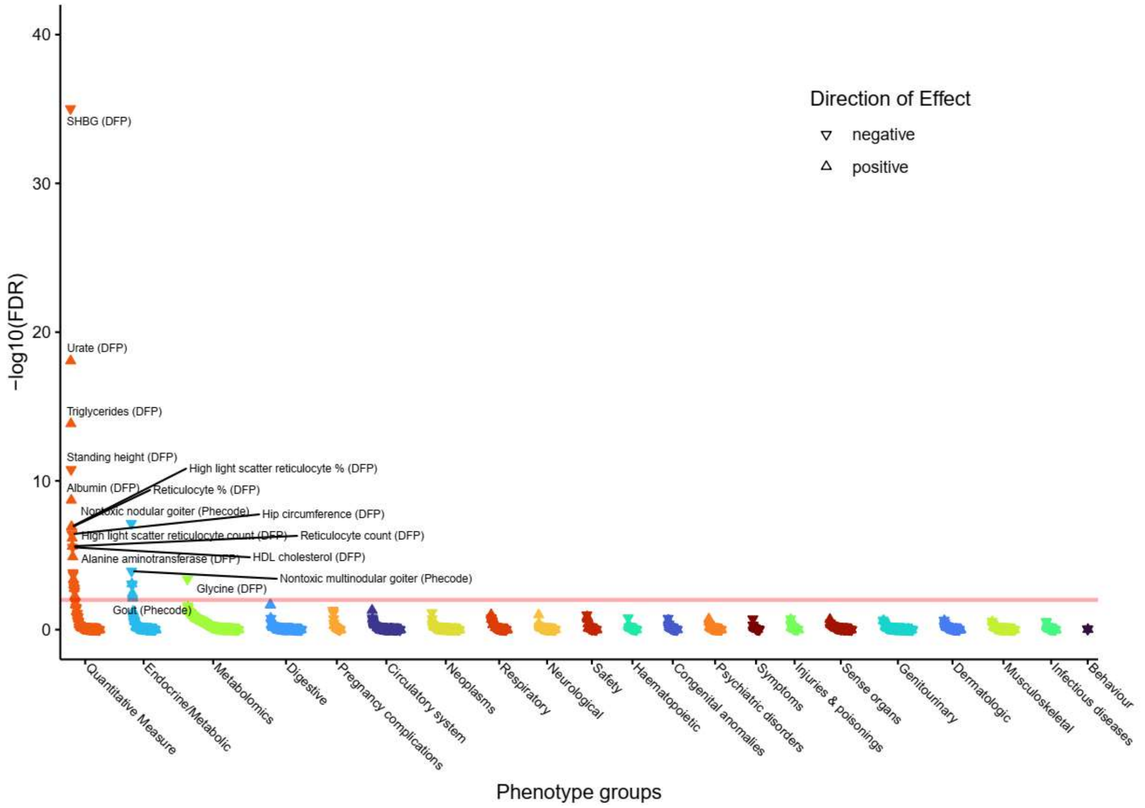

p) rs7306508 (implicating *KRT18*).

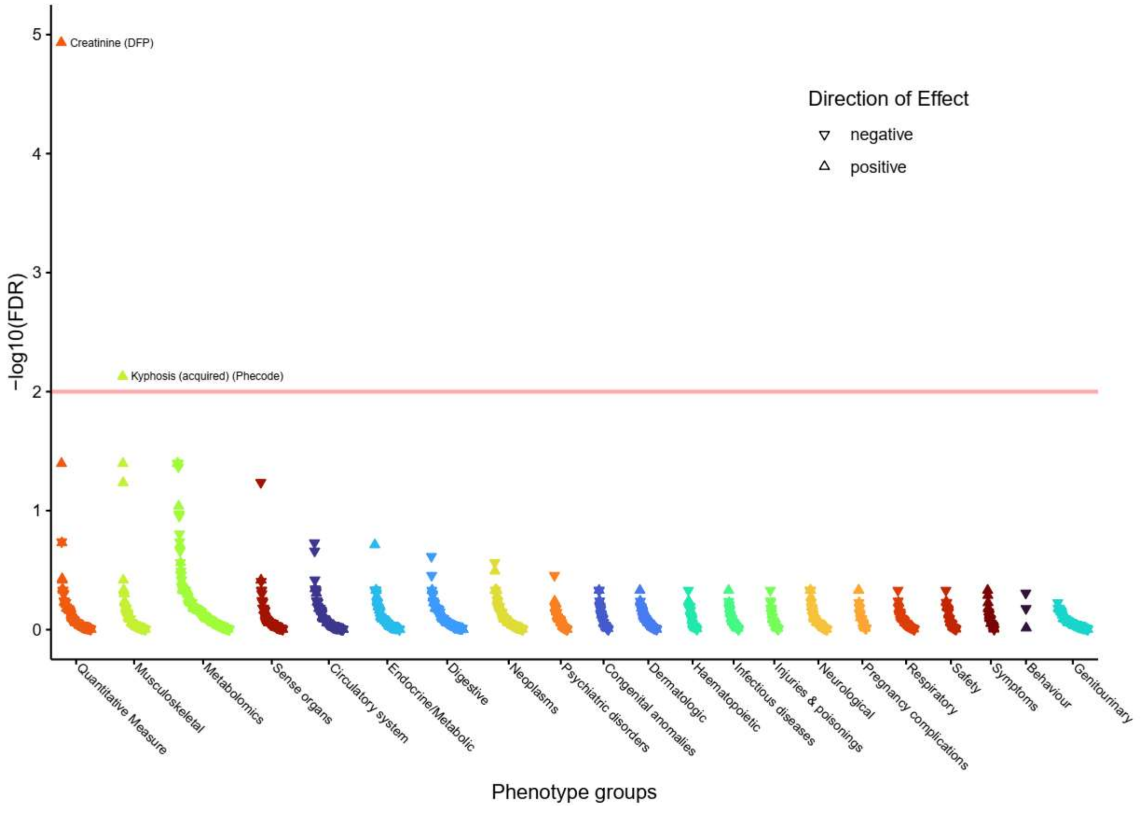

q) rs2393717 (implicating *MLEC* and *SPPL3*).

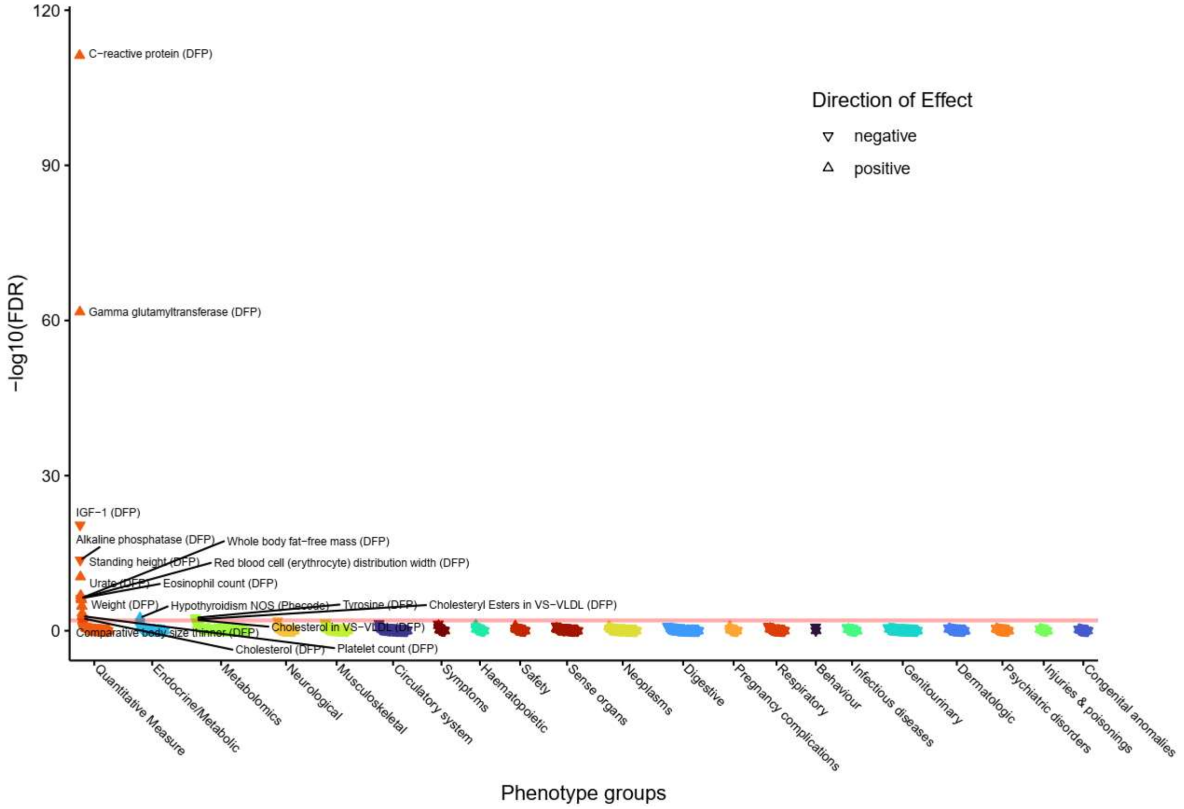

r) rs36213229 (implicating *NRG1*).

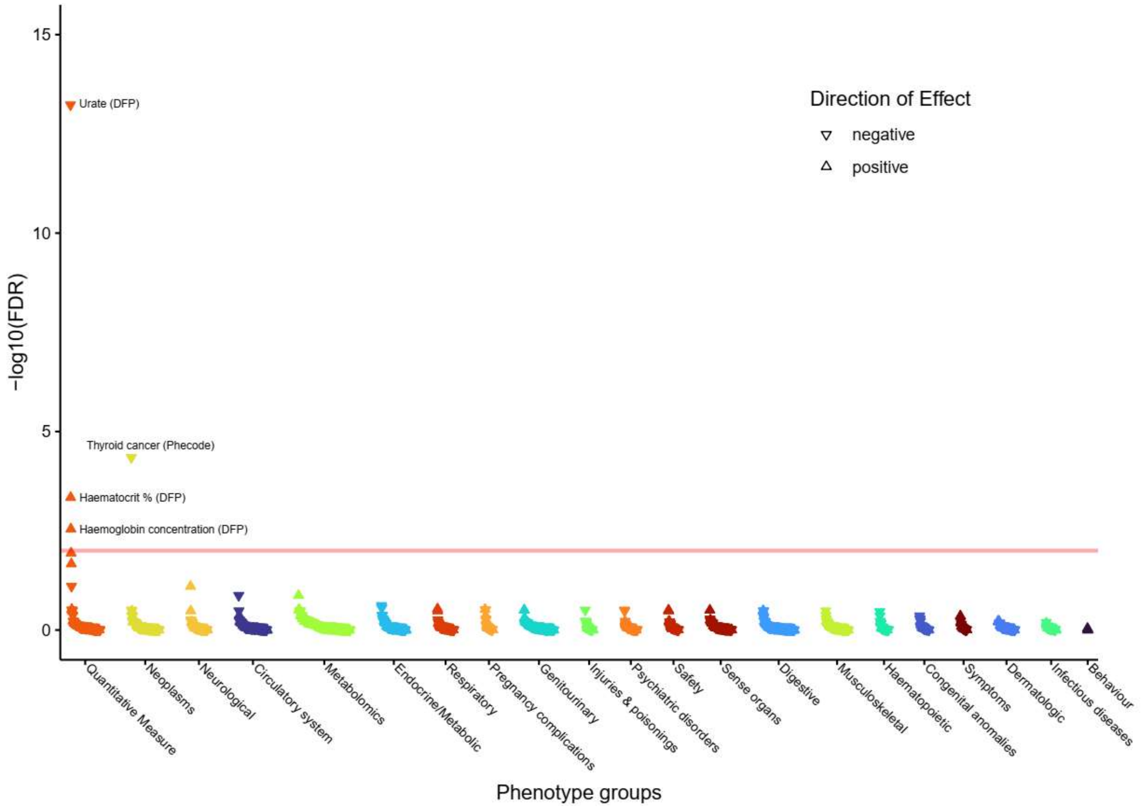

s) rs68030583 (implicating *NRG1*).

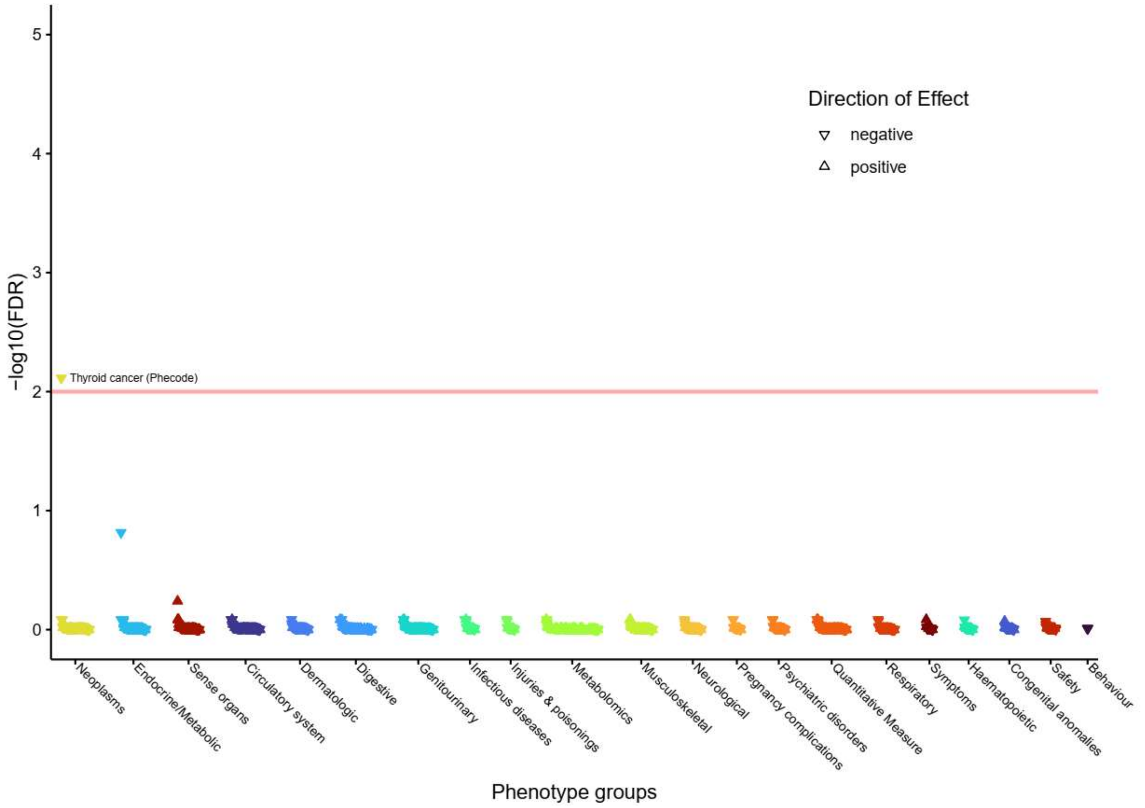

t) rs11554674 (implicating *PHC2*, *ZNF362* and *A3GALT2*).

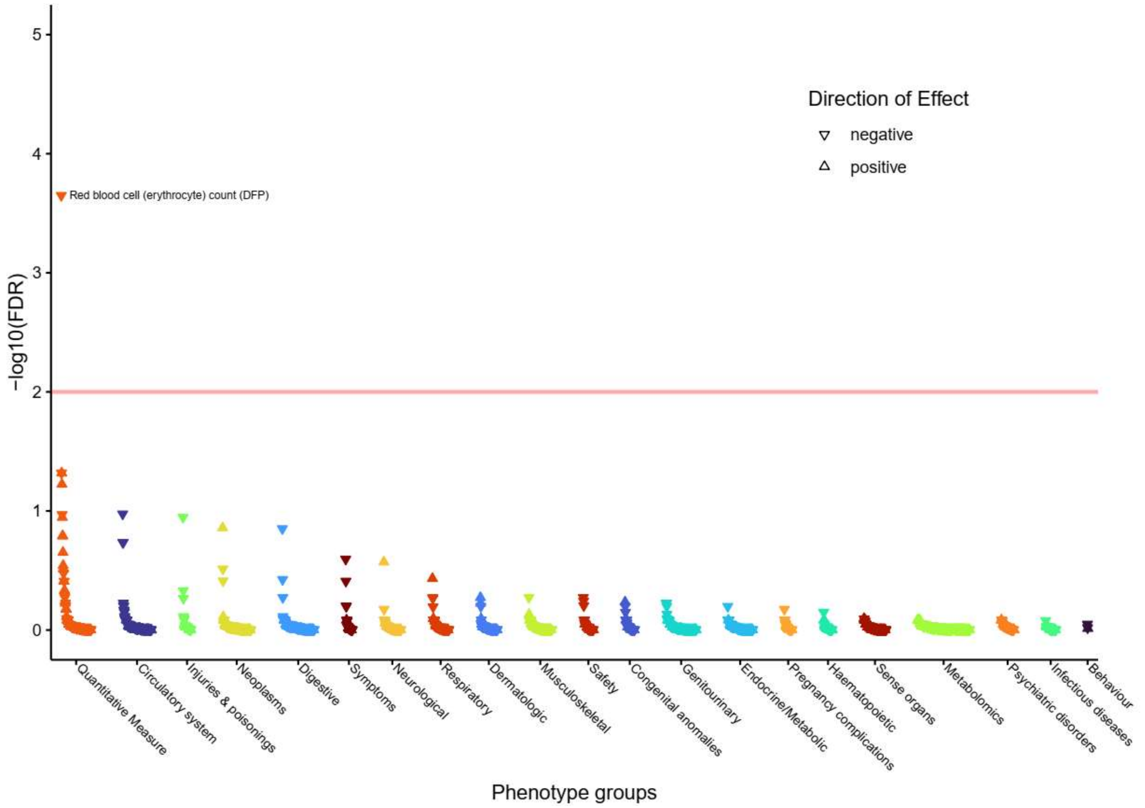

u) rs1801690 (implicating *PRKCA* and *APOH*).

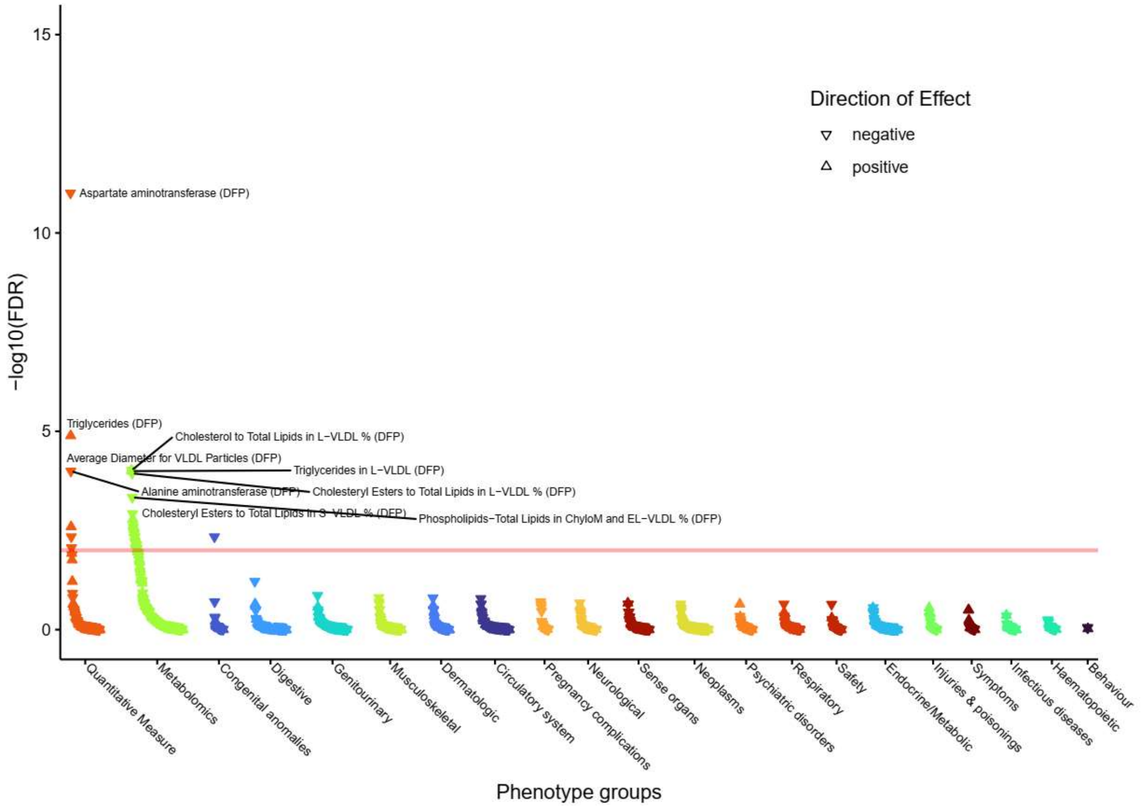

v) rs9497965 (implicating *SASH1*).

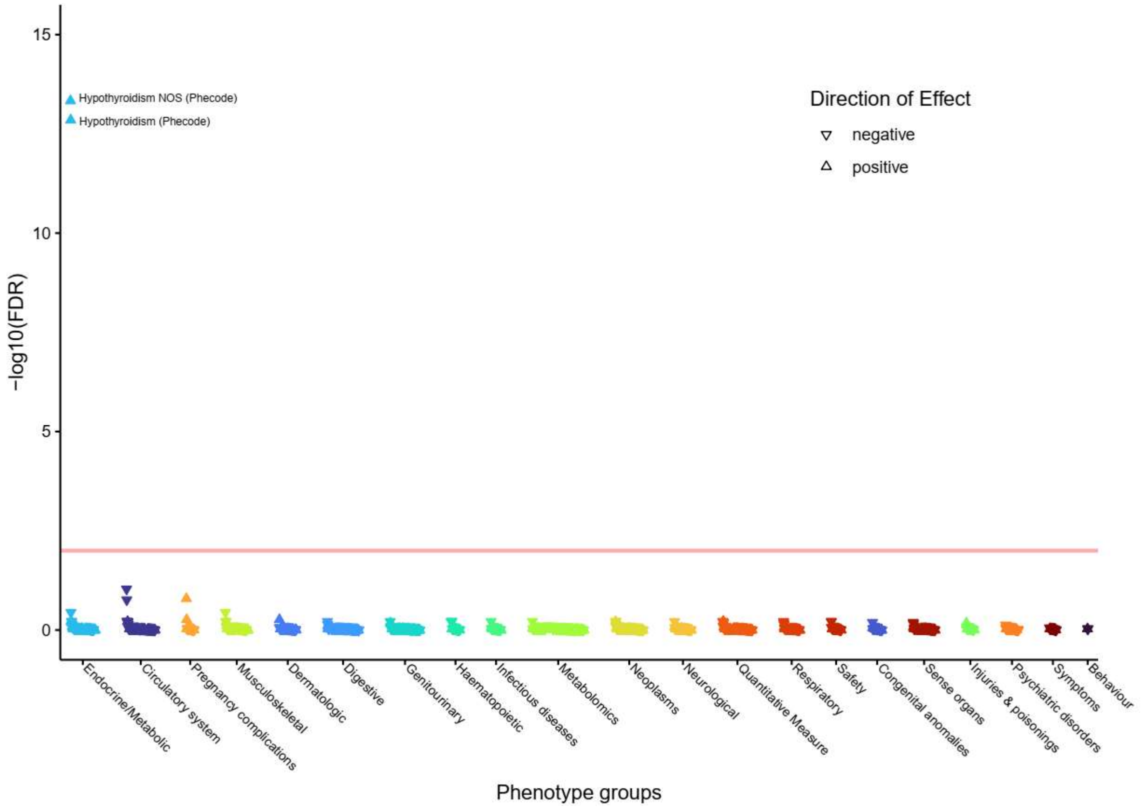

w) rs10926981 (implicating *SDCCAG8*).

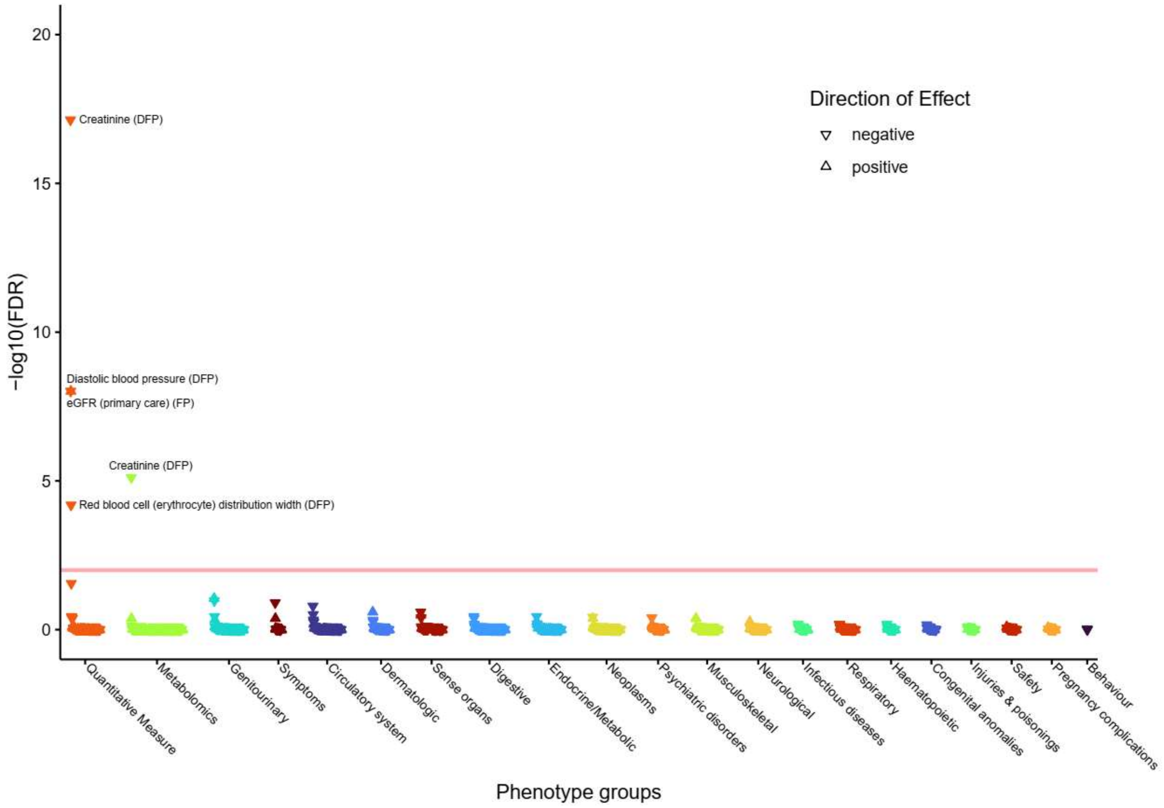

x) rs1743963 (implicating *SGK1*).

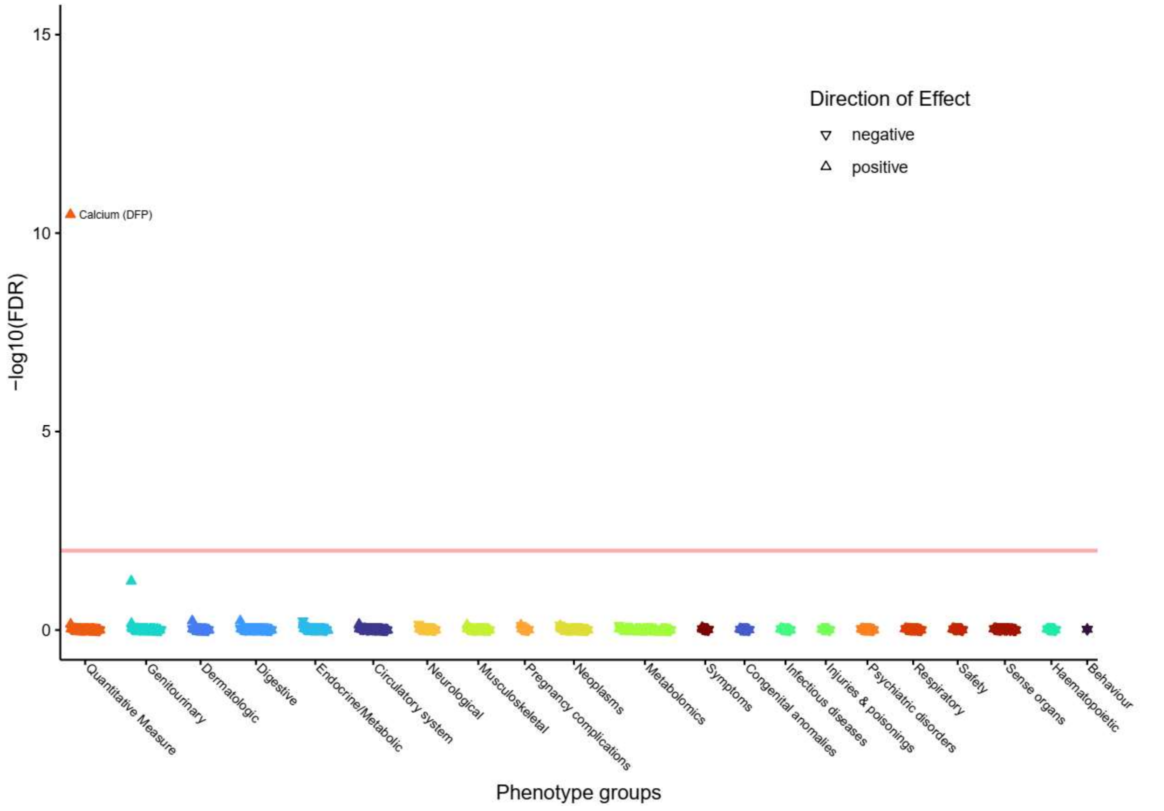

y) rs3184504 (implicating *SH2B3*).

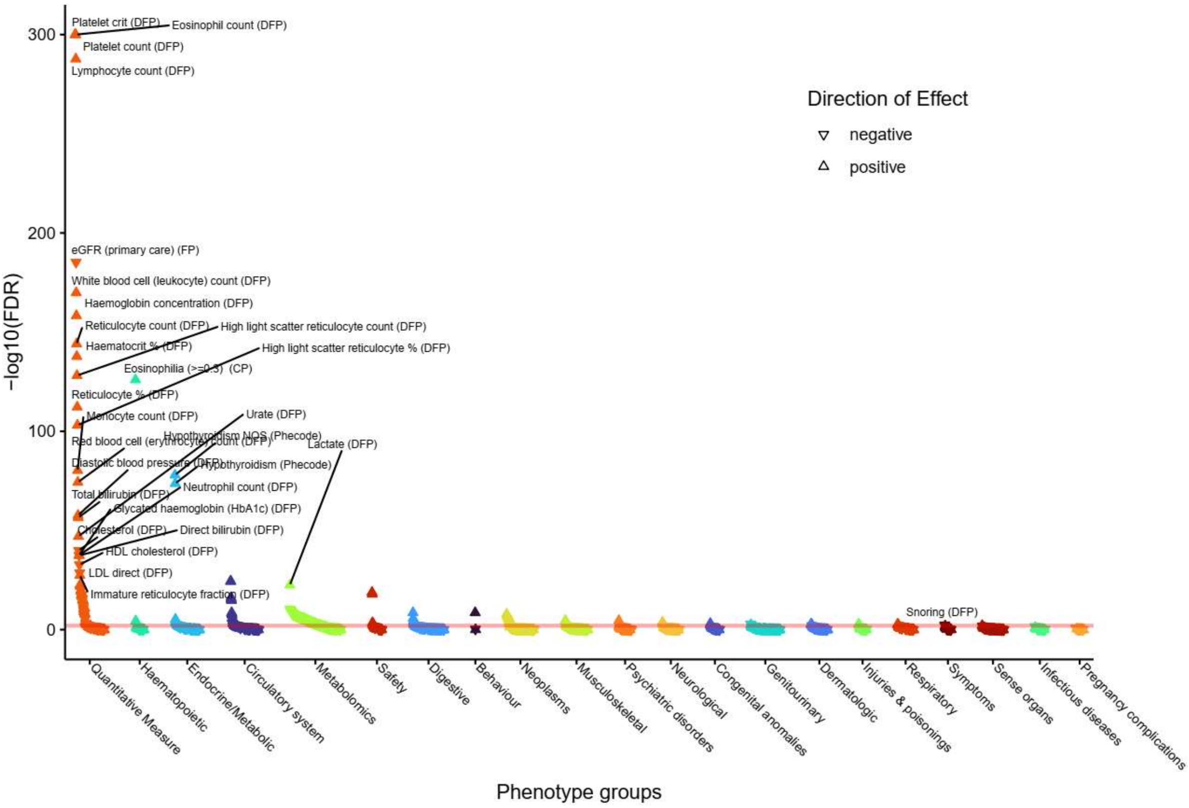

z) rs751171 (implicating *SMOC2*).

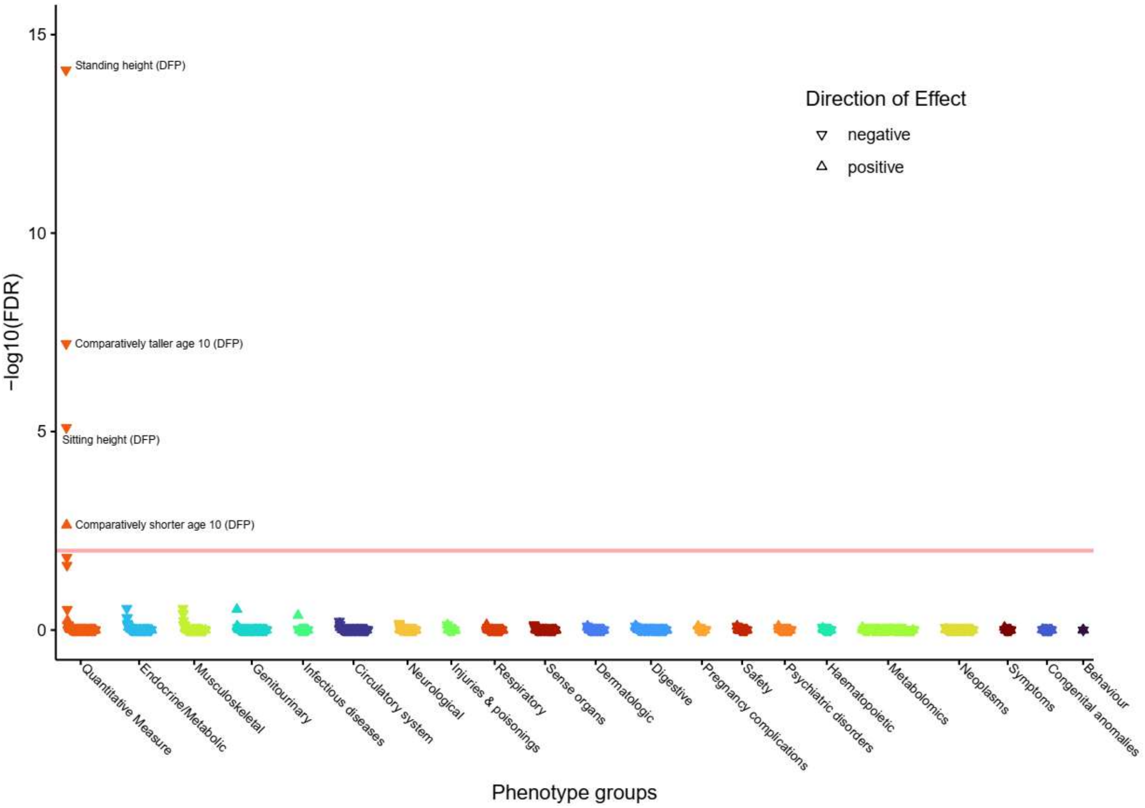

aa) rs9507279 (implicating *SPATA13*).

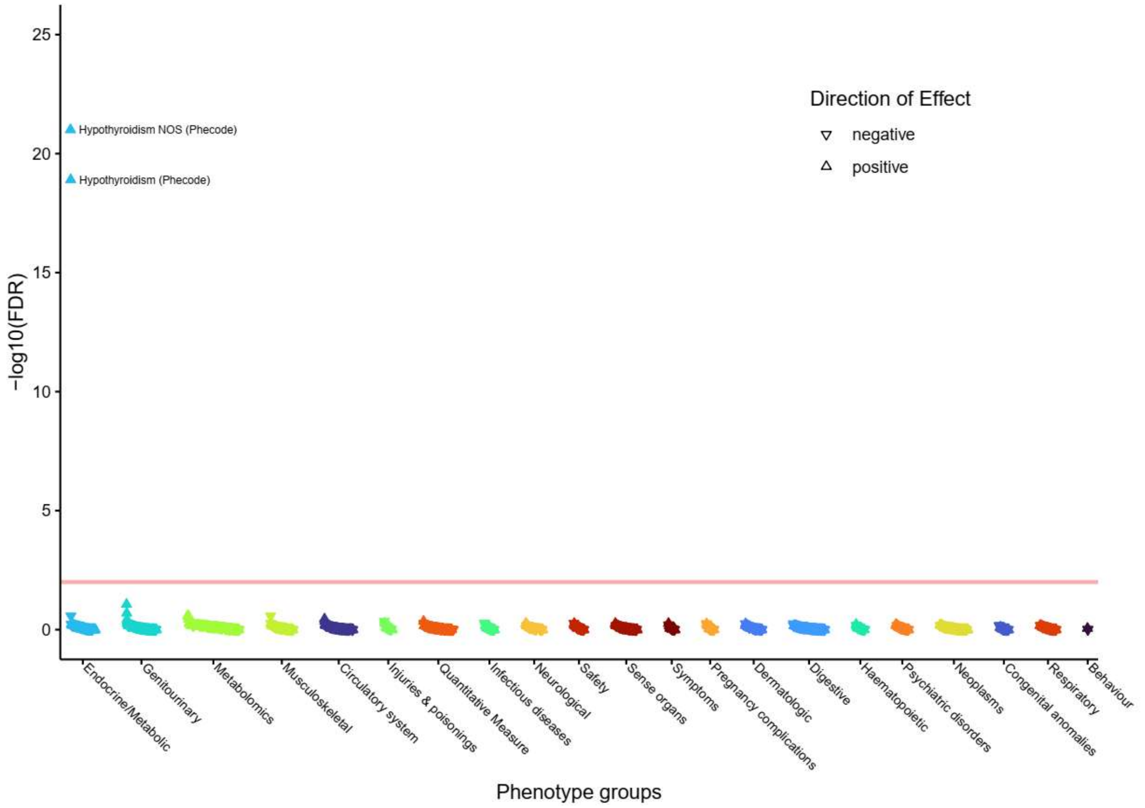

bb) rs17364832 (implicating *SPATA13*).

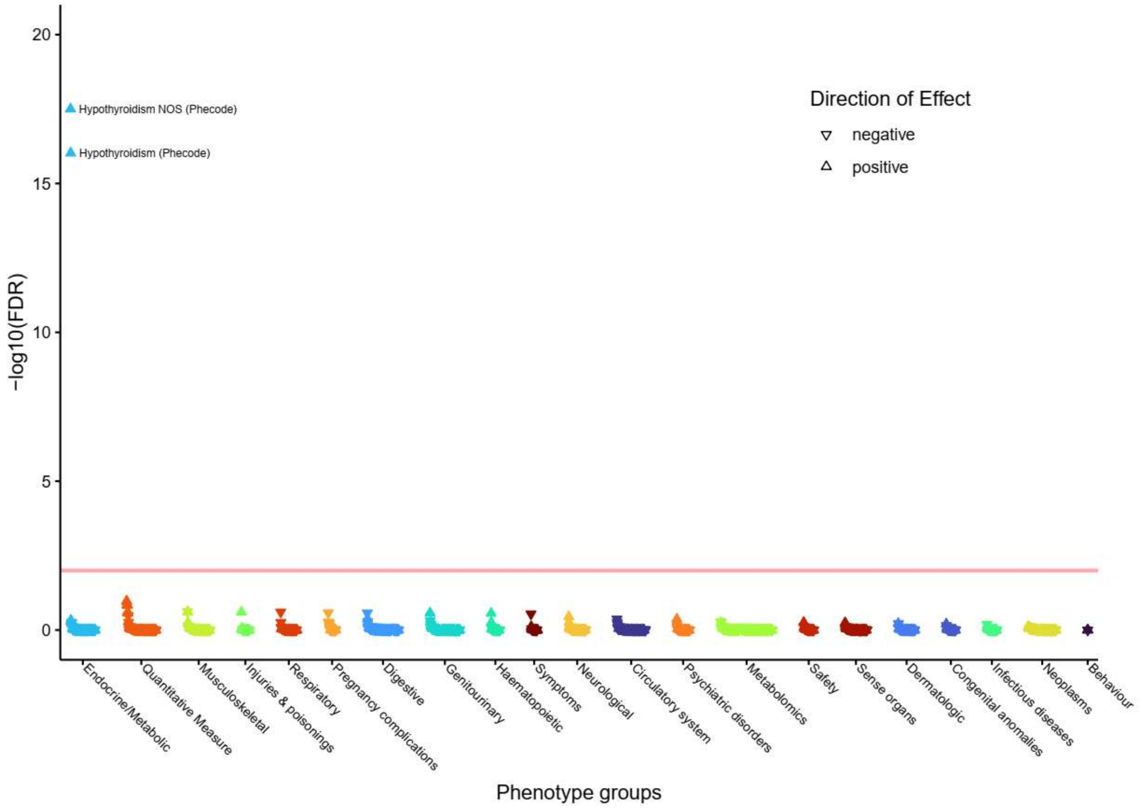

cc) rs10113355 (implicating *SULF1* and *LOC100505739*).

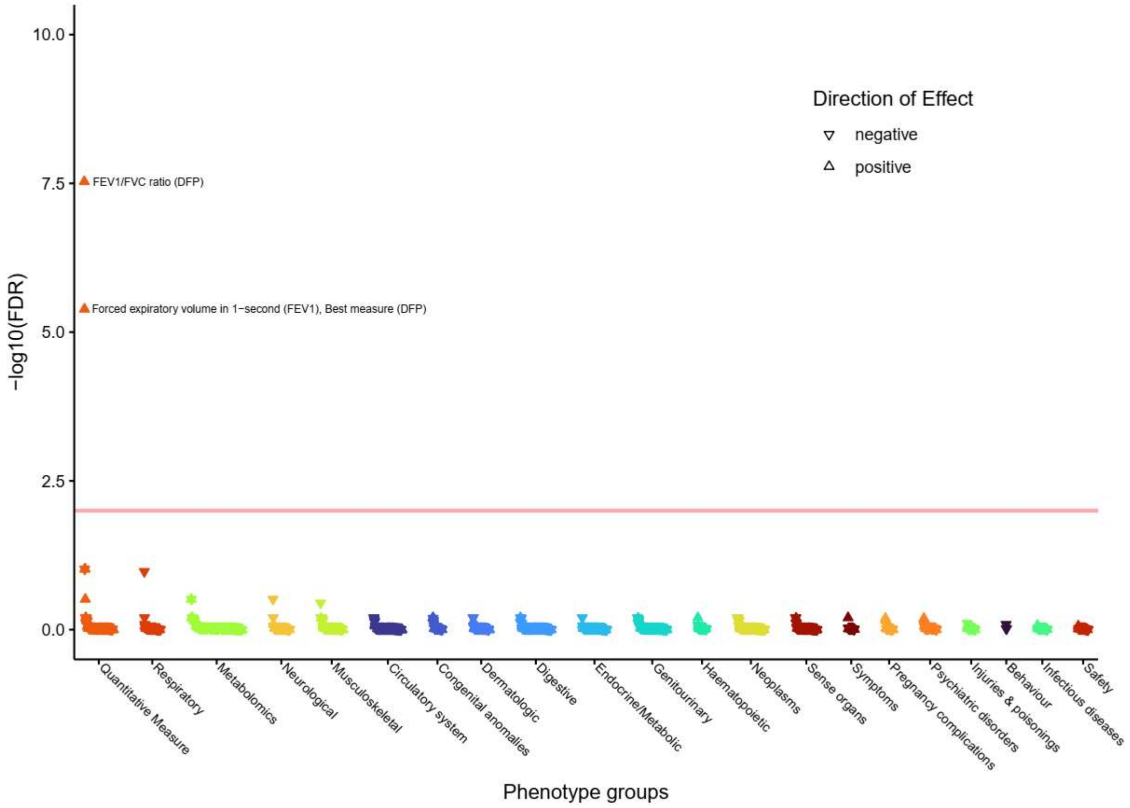

dd) rs2069556 (implicating *TG* and *CCN4*).

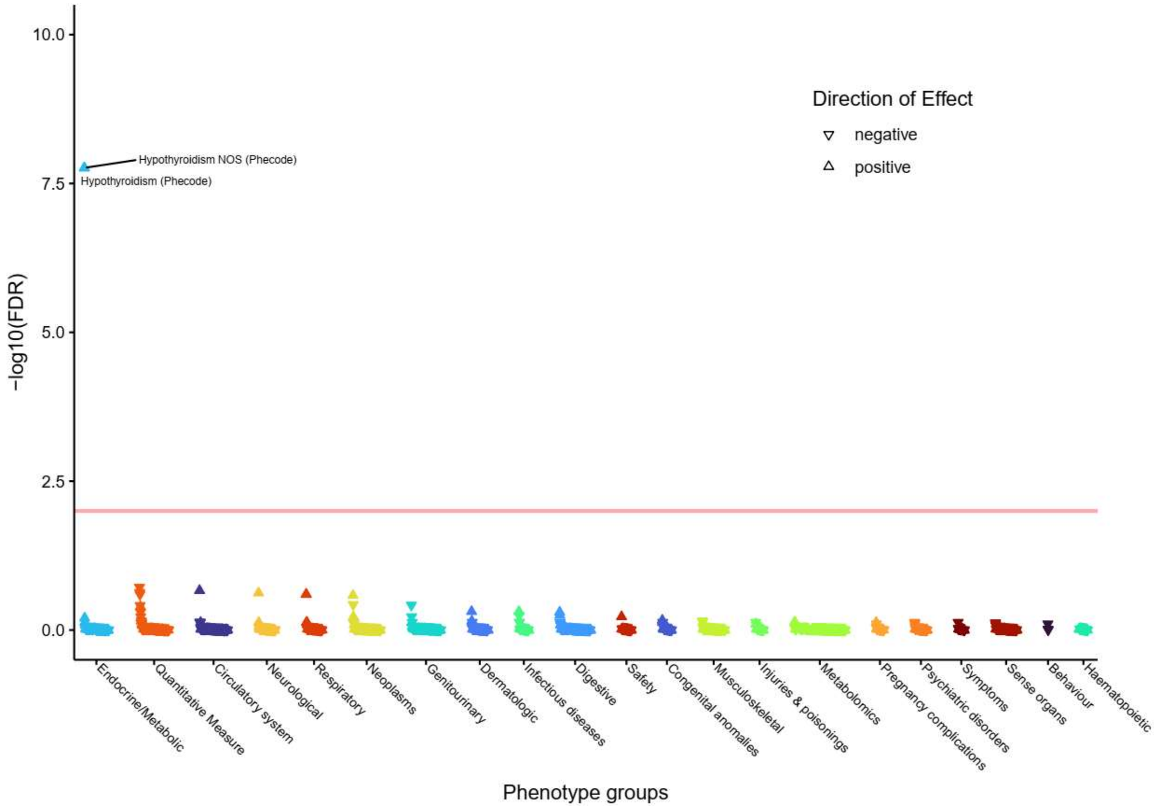

ee) rs2069568 (implicating *TG* and *CCN4*).

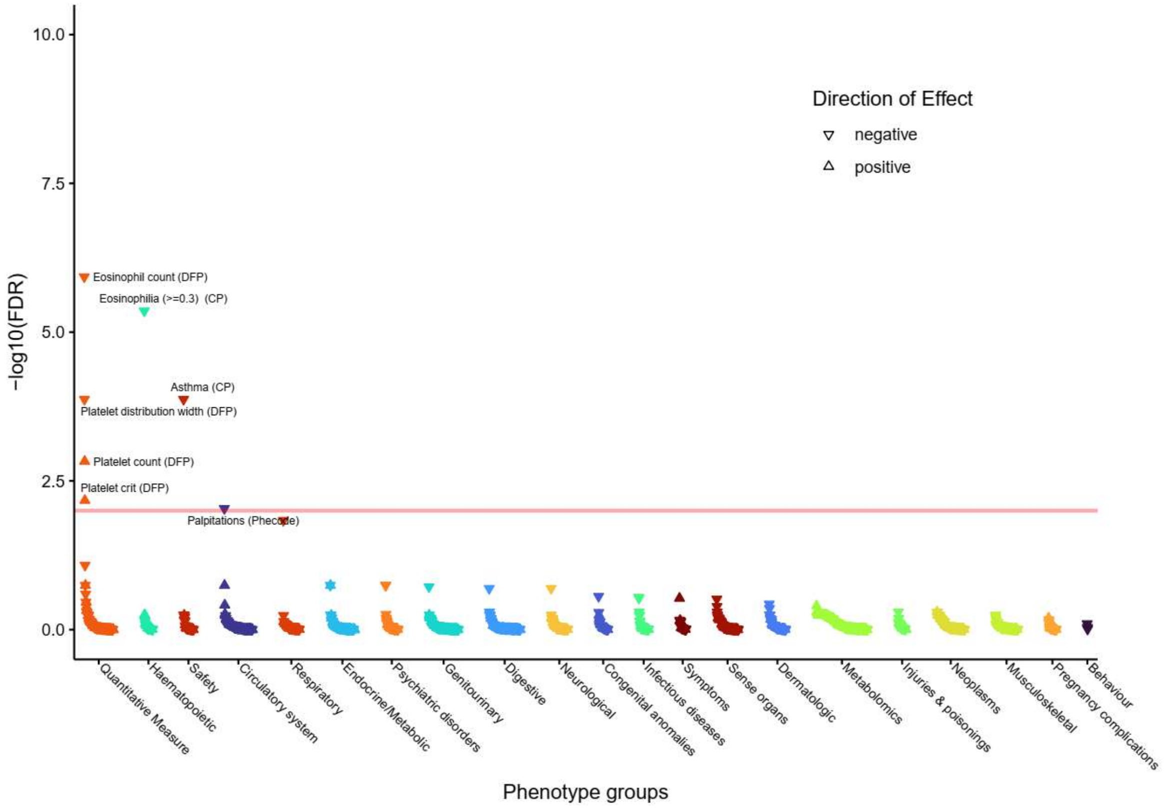

ff) rs114322847 (implicating *TG* and *CCN4*).

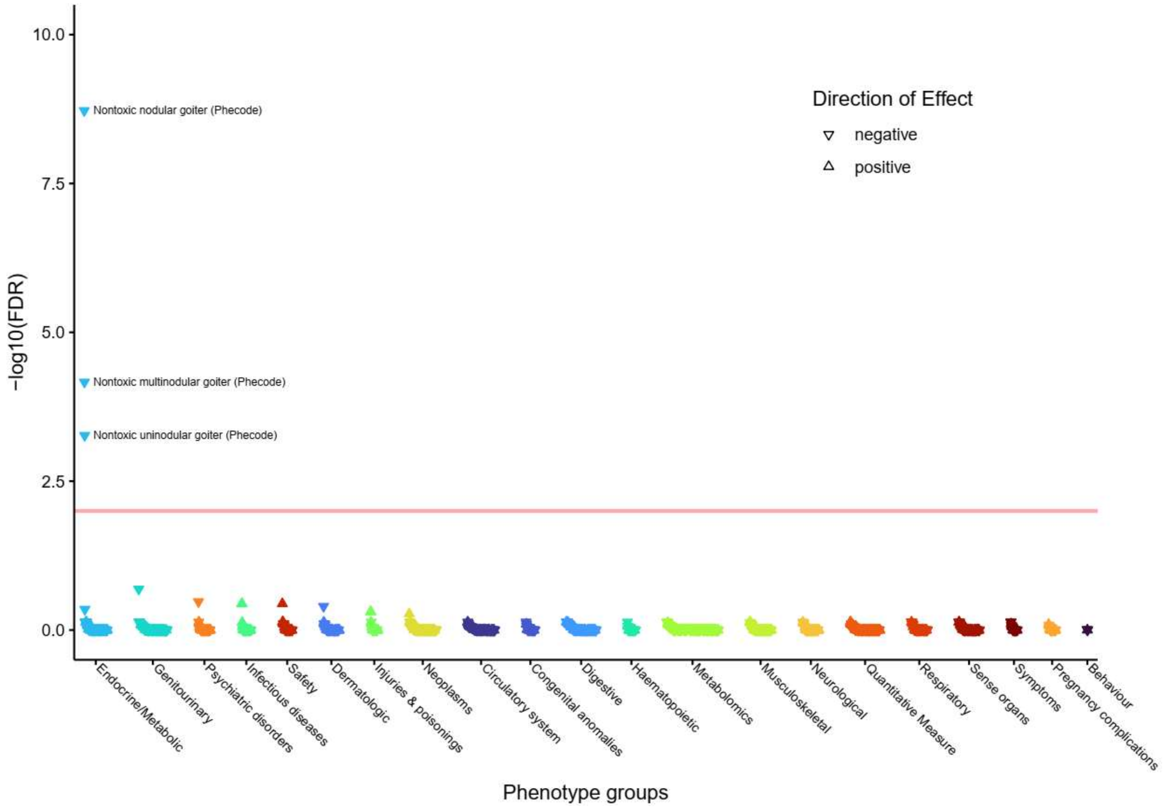

gg) rs519709 (implicating *TGFB2* and *MIR548F3*).

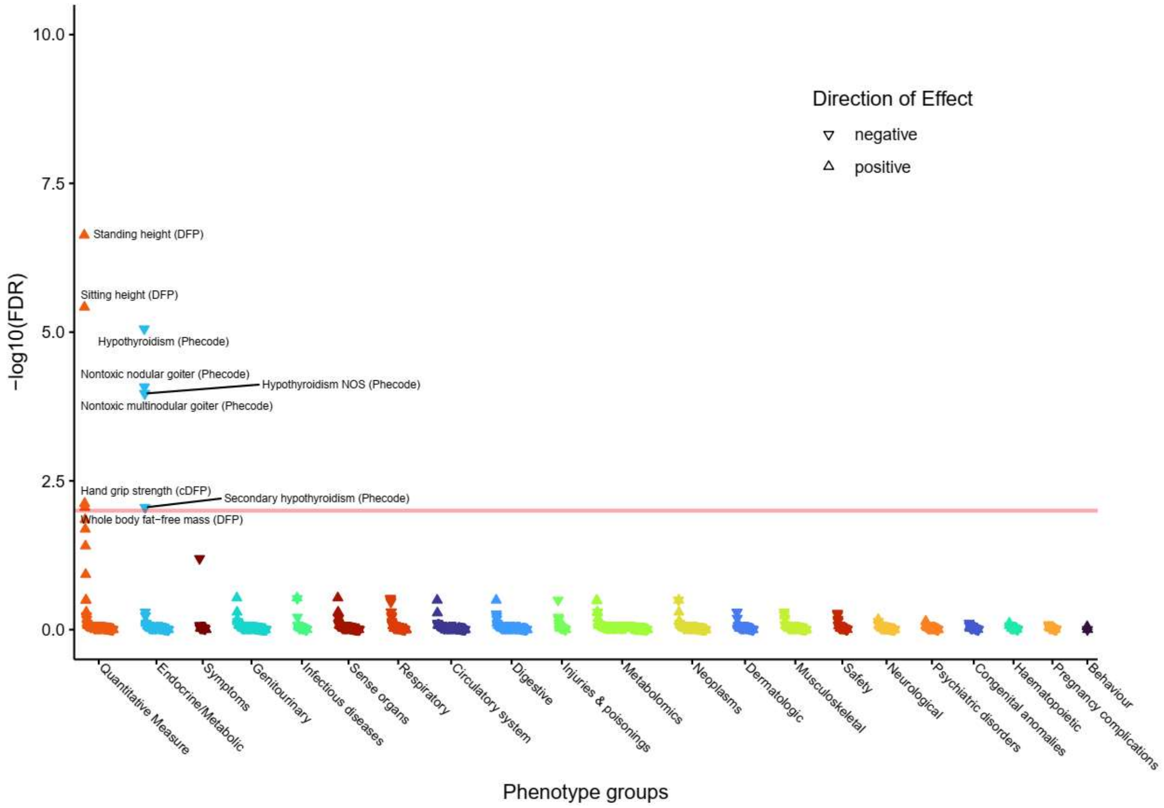

hh) rs11694732 (implicating *TPO*).

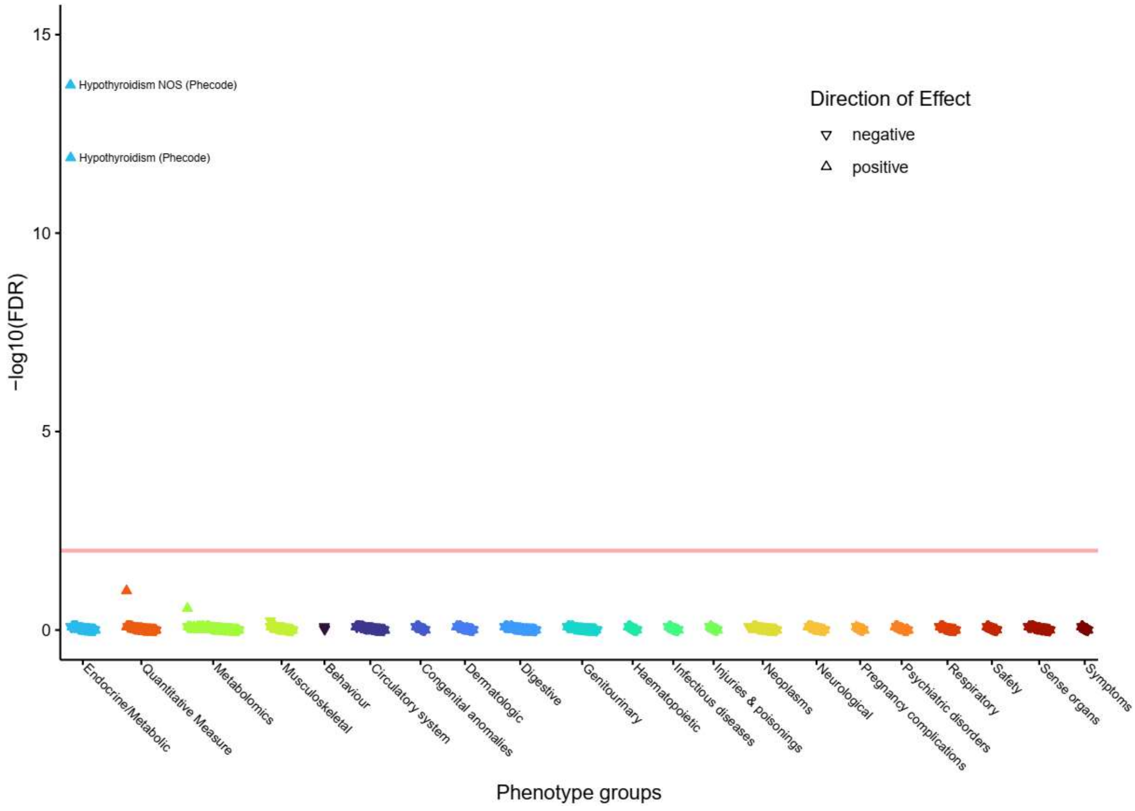

ii) rs3742721 (implicating *TSHR* and *CEP128*).

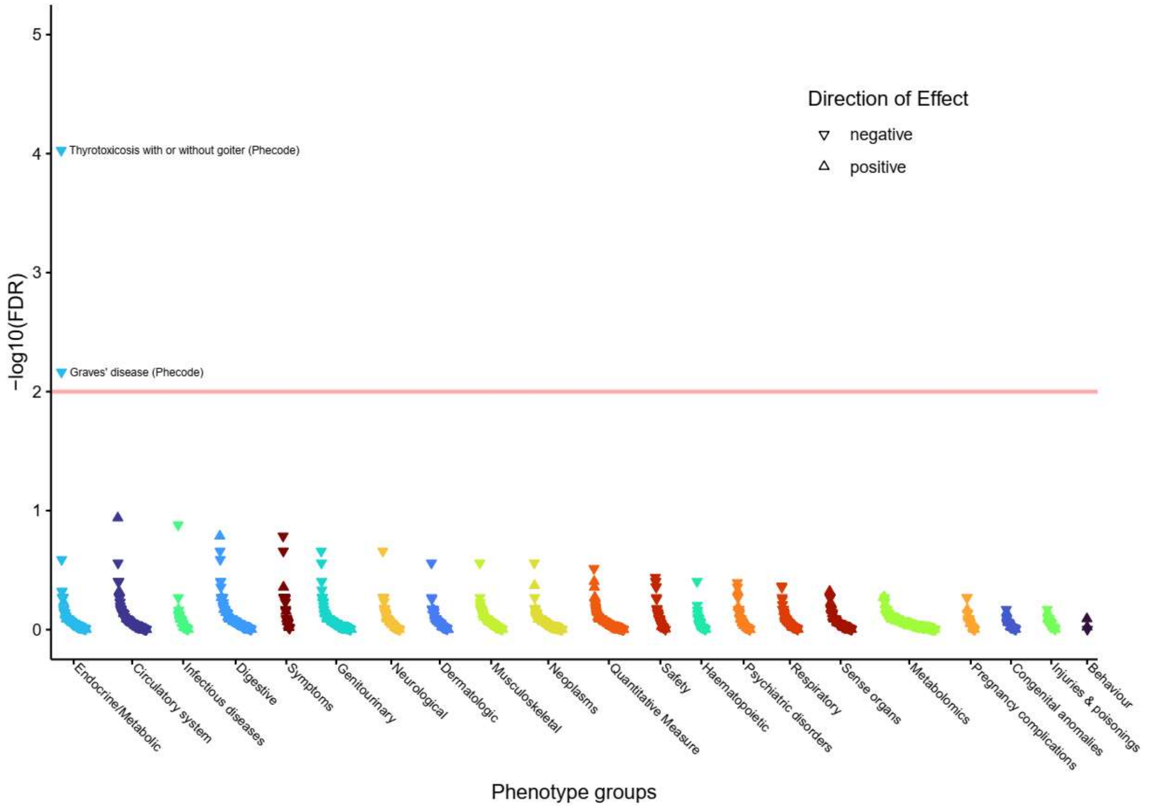

jj) rs6574611 (implicating *TSHR* and *CEP128*).

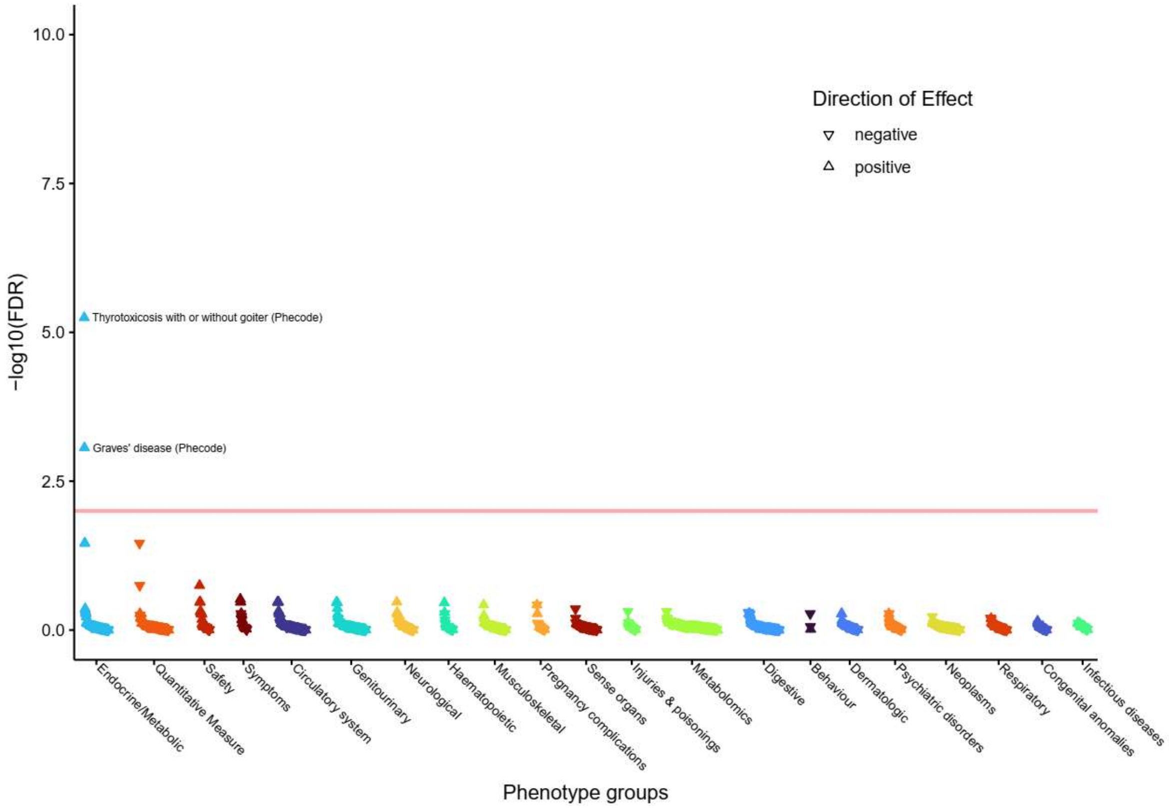

kk) rs2110696 (implicating *TSHR*).

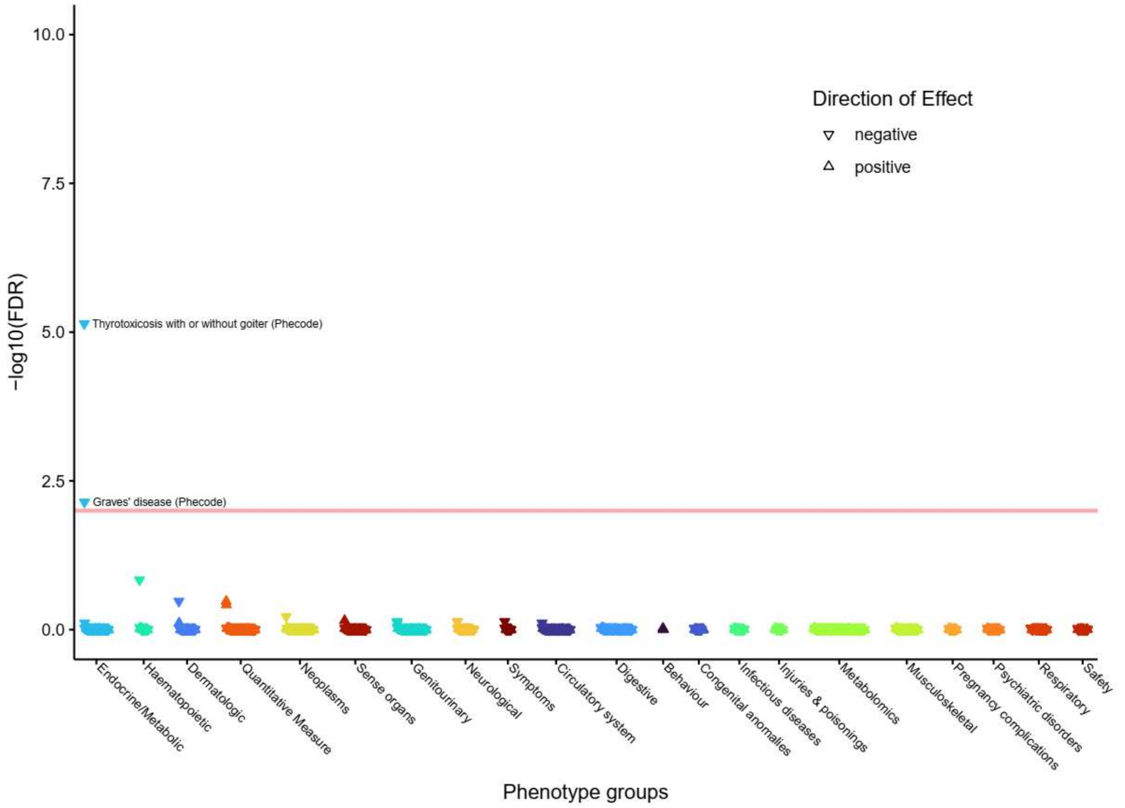

ll) rs1802288 (implicating *TSPAN6*).

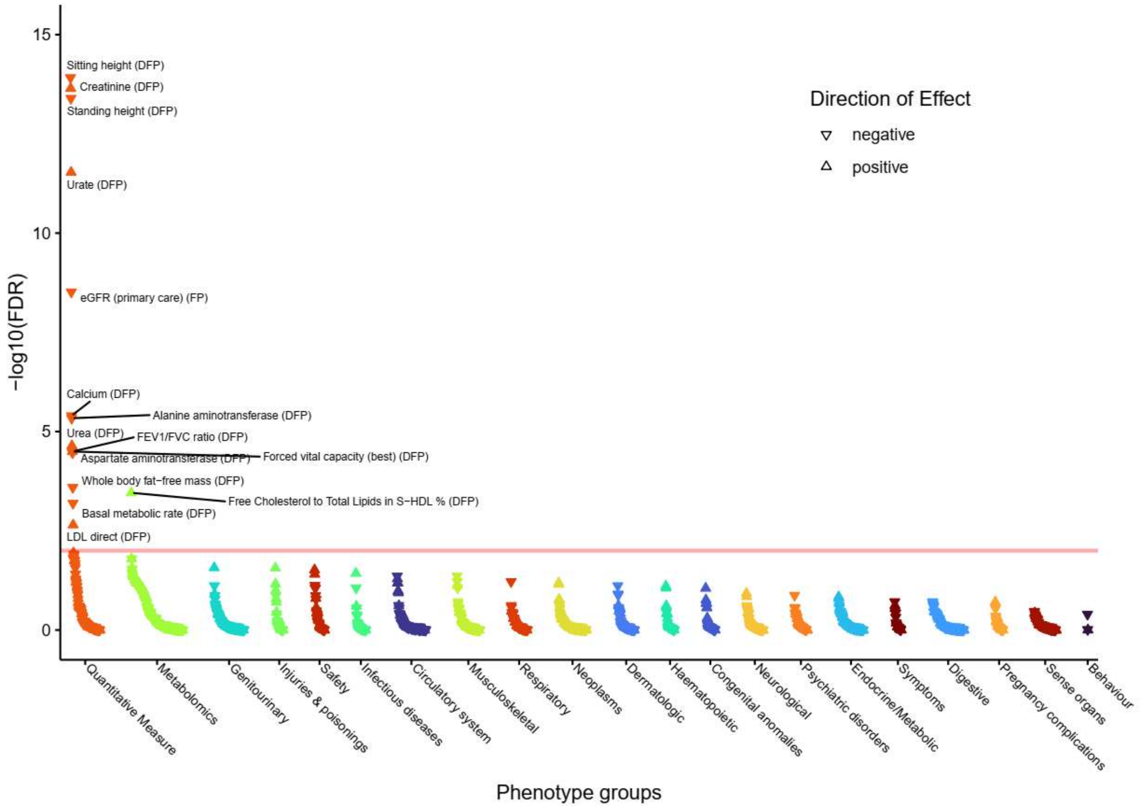

mm) rs3755972 (implicating *VEGFC*).

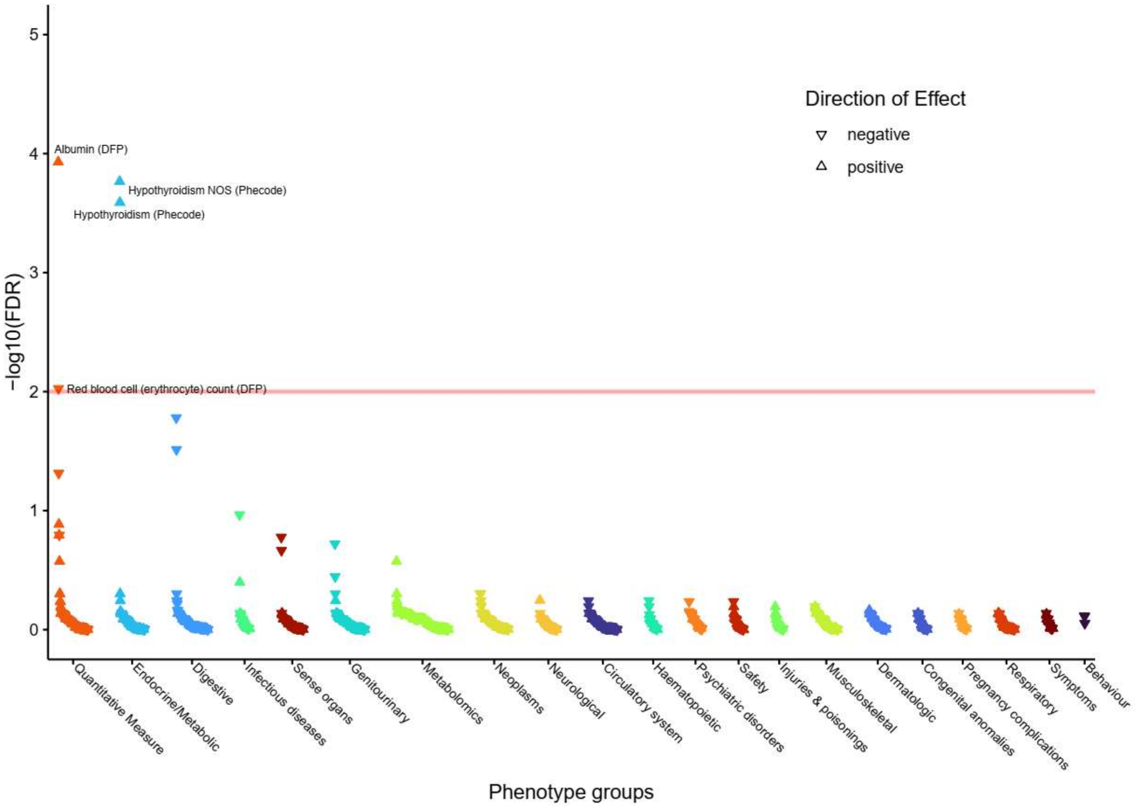

Direction of Effect
negative
positive
-log10(FDR)
Albumin (DFP)
Hypothyroidism NOS (Phecode)
Hypothyroidism (Phecode)
Red blood cell (erythrocyte) count (DFP)
Quantitative Measure
Endocrine/Metabolic
Digestive
Infectious diseases
Sense organs
Genitourinary
Metabolomics
Neoplasms
Neurological
Circulatory system
Haematopoietic
Psychiatric disorders
Safety
Injuries & poisonings
Musculoskeletal
Dermatologic
Congenital anomalies
Pregnancy complications
Respiratory
Symptoms
Behaviour
Phenotype groups

Supplementary Figure 3: 112 genes prioritised by two or more variant-to-gene criteria. The first seven columns indicate that at least one variant implicates the corresponding gene via the evidence for that column. The remaining seven columns indicate the strength of association of the most significant variant implicating the corresponding gene as causal with respect to the TSH increasing allele, such that shades of blue represent associations with the other thyroid phenotypes that have the same direction of effect as the TSH association and shades of red represent an opposite direction of effect to the TSH association. hypo_due_to_Rx = hypothyroidism due to treatment/medication/ablation

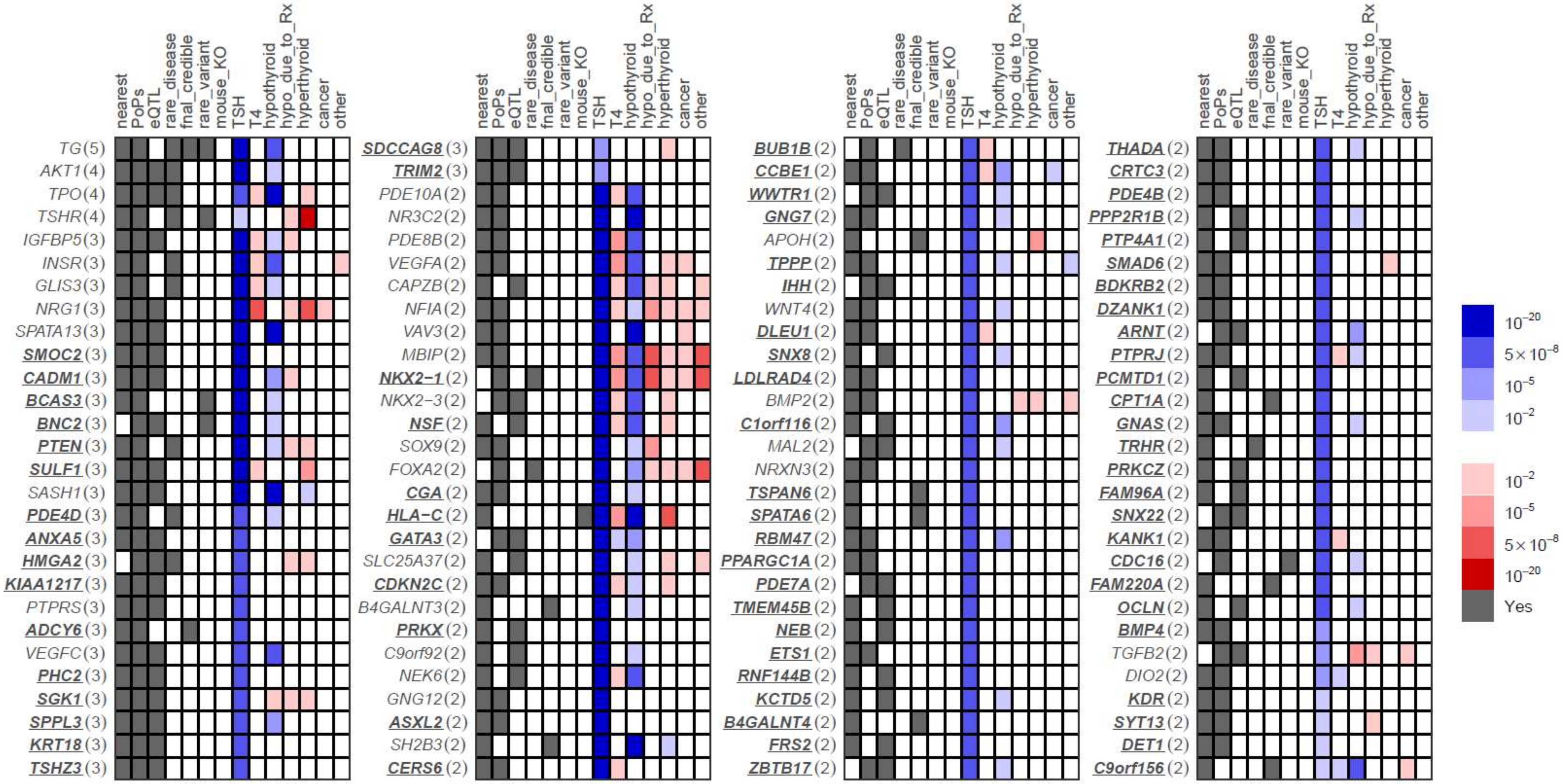

Supplementary Figure 4: Pathway-specific polygenic score phenome-wide association studies (PheWAS)

a) **cAMP (KEGG) signalling pathway**

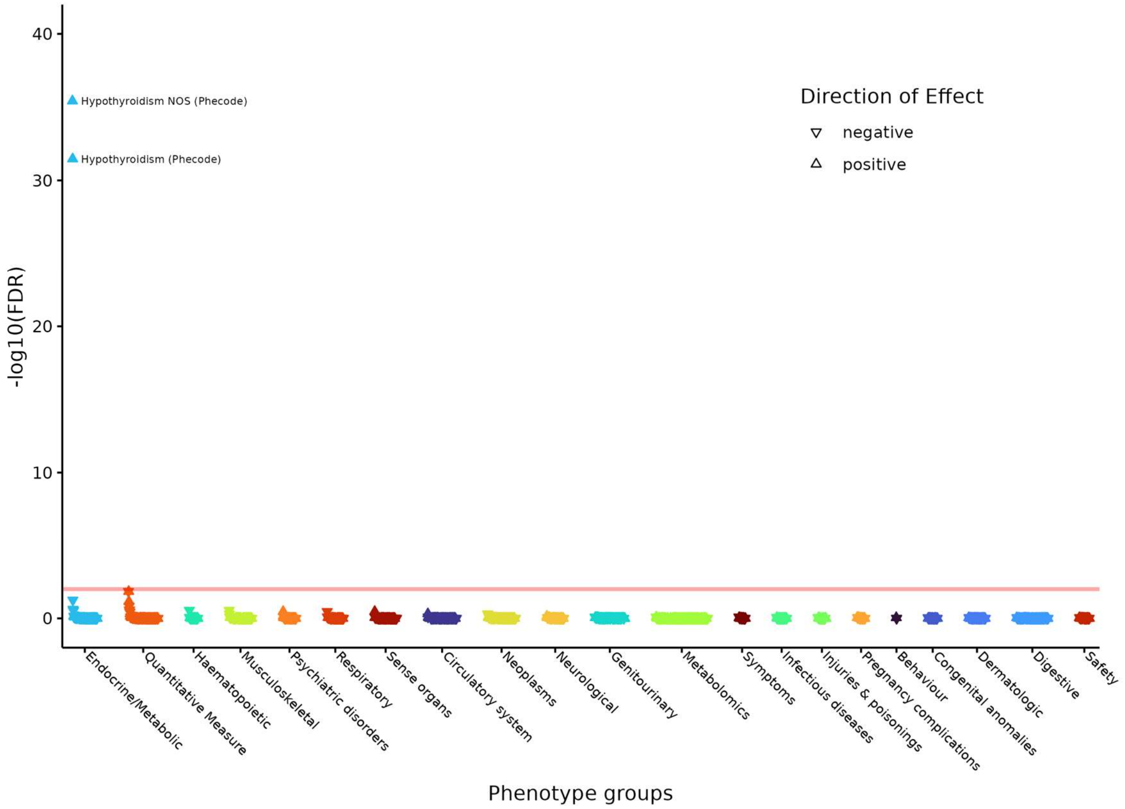

b) **ADORA2B mediated anti-inflammatory cytokines production pathway.**

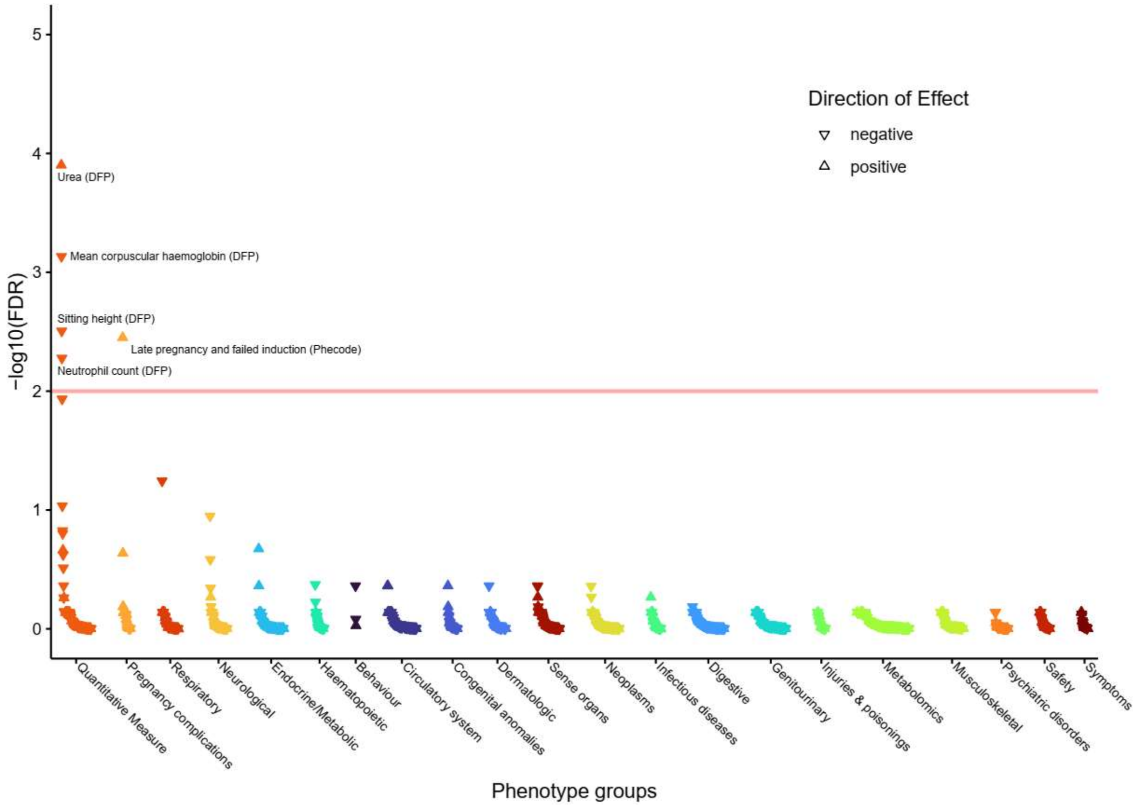

c) **Apoptosis-related network due to altered Notch3 in ovarian cancer pathway.**

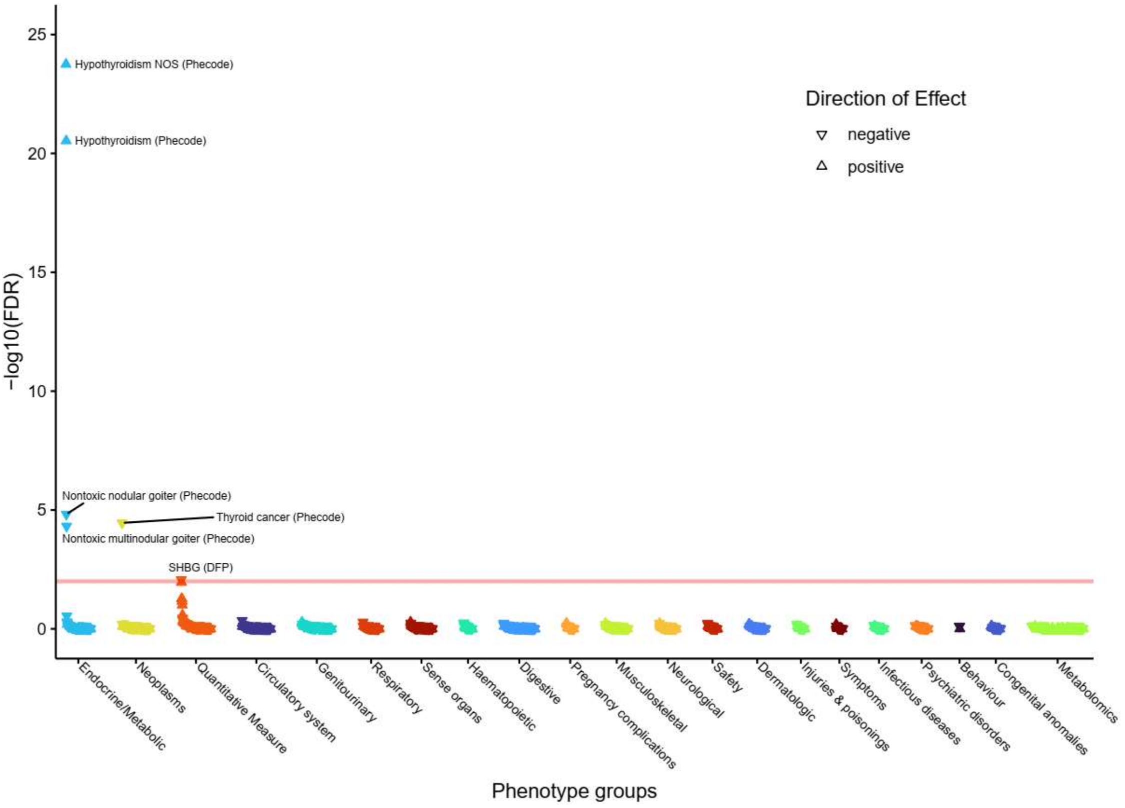

d) **Factors and pathways affecting insulin-like growth factor (IGF1)-Akt signaling pathway.**

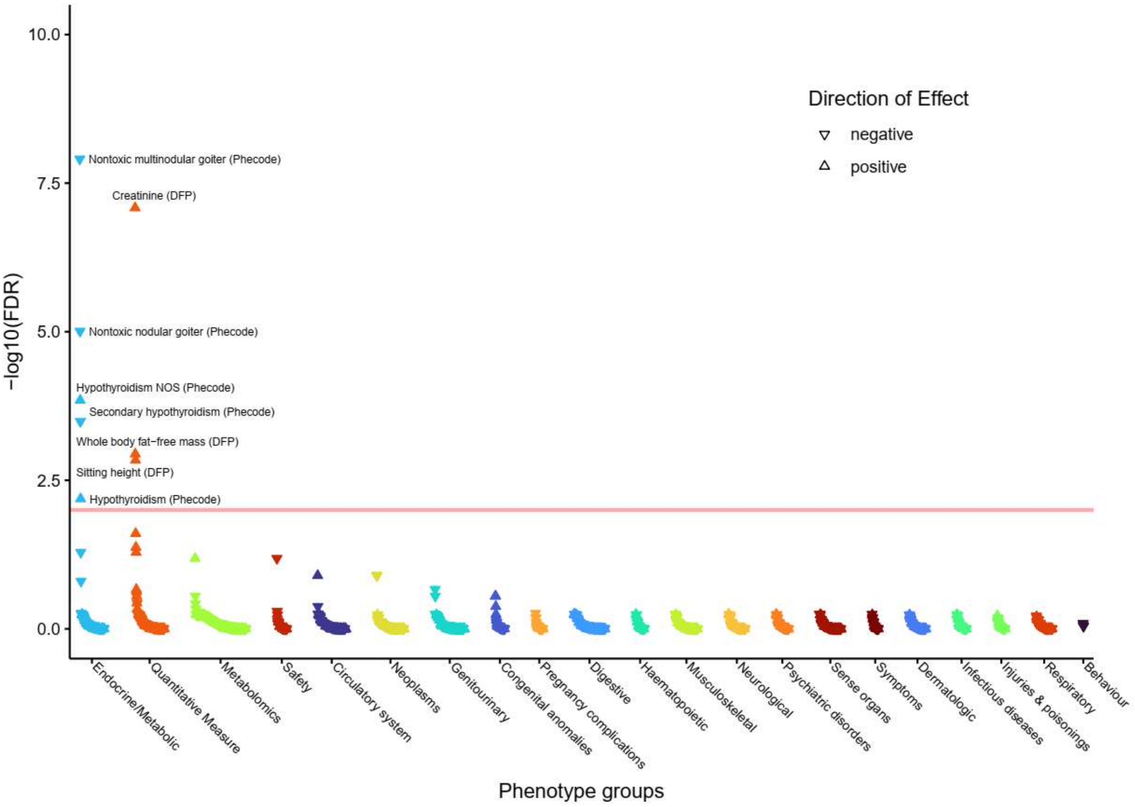

e) FGFR3 signaling in chondrocyte proliferation and terminal differentiation pathway.

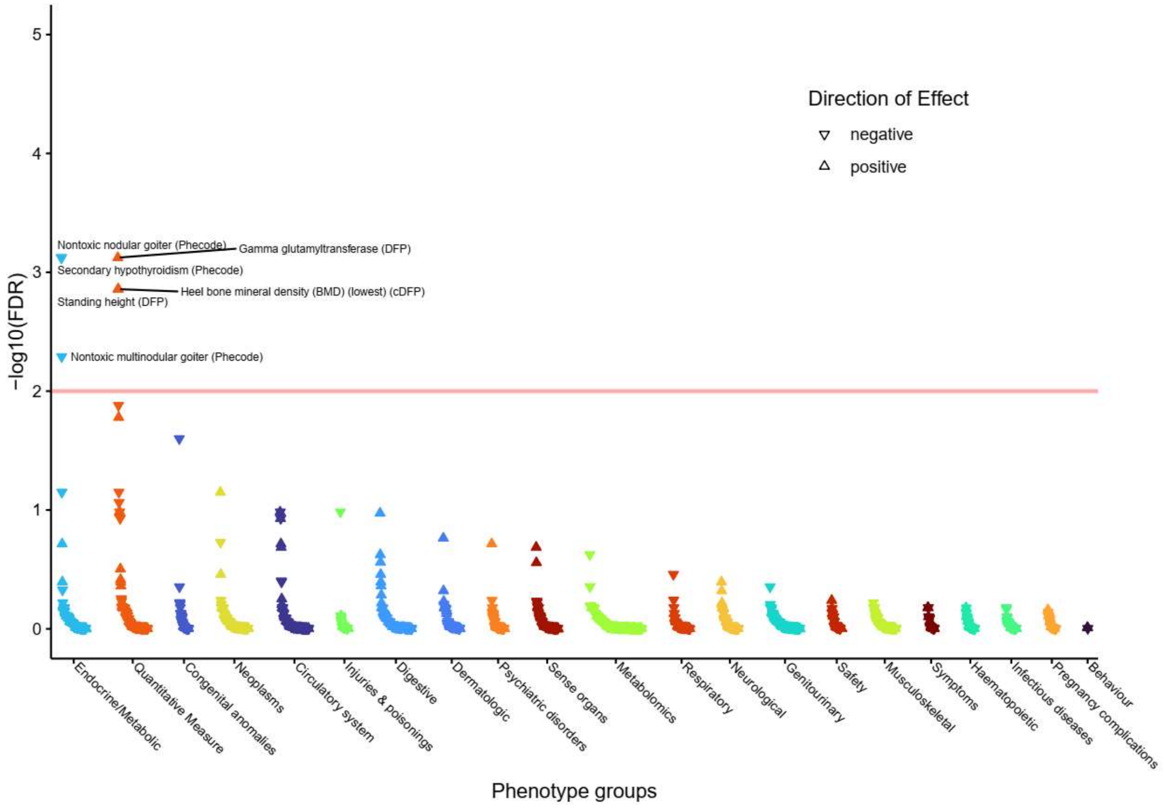

f) Focal Adhesion-PI3K-Akt-mTOR-signaling pathway.

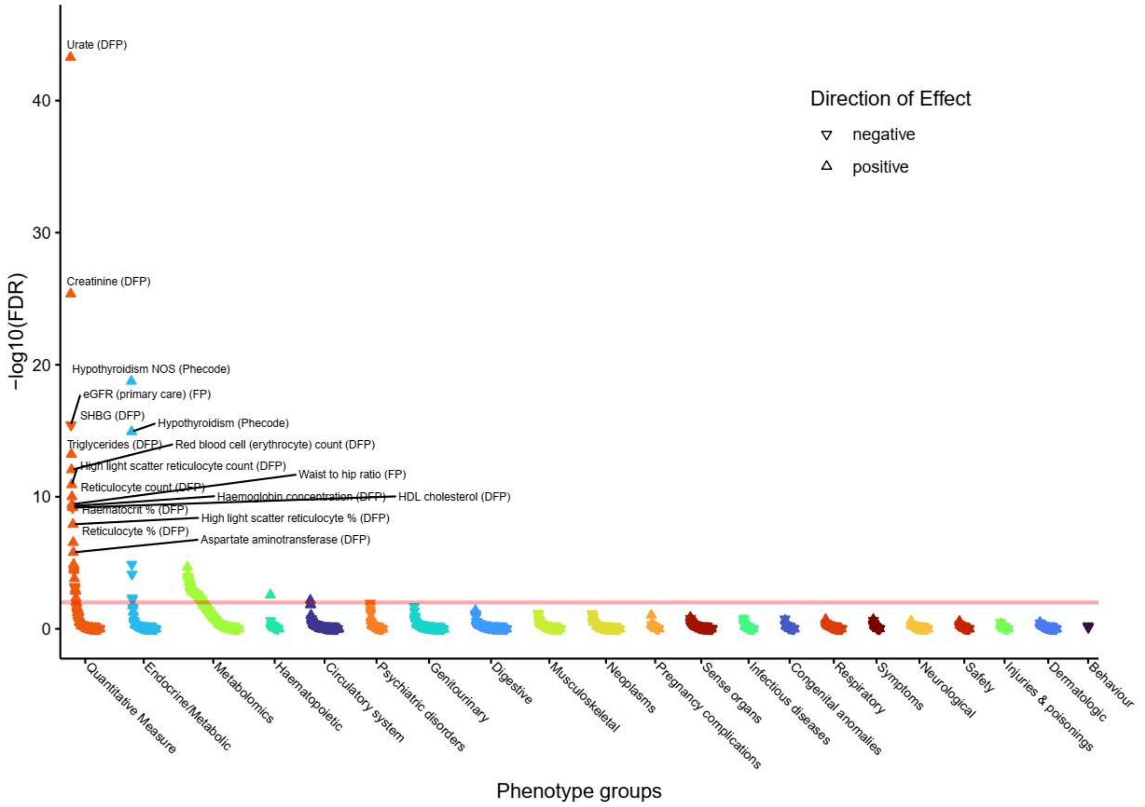

g) G Protein Signaling Pathways pathway.

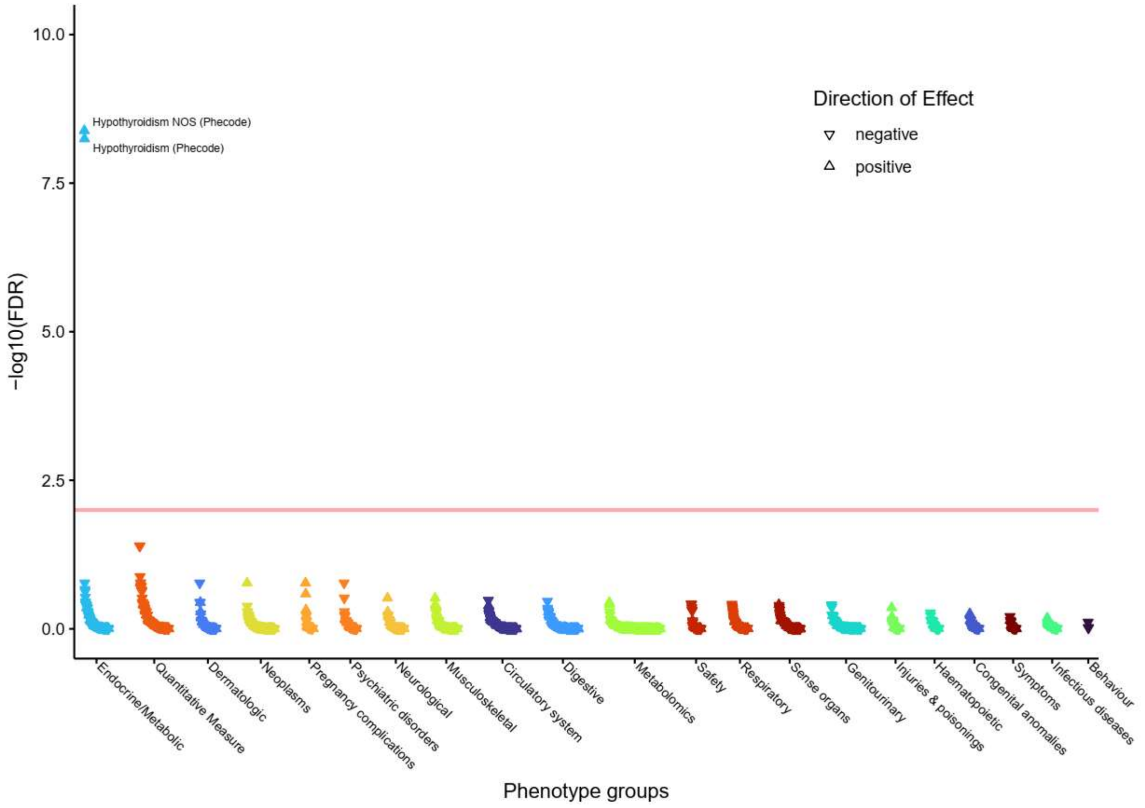

h) Heart Development pathway.

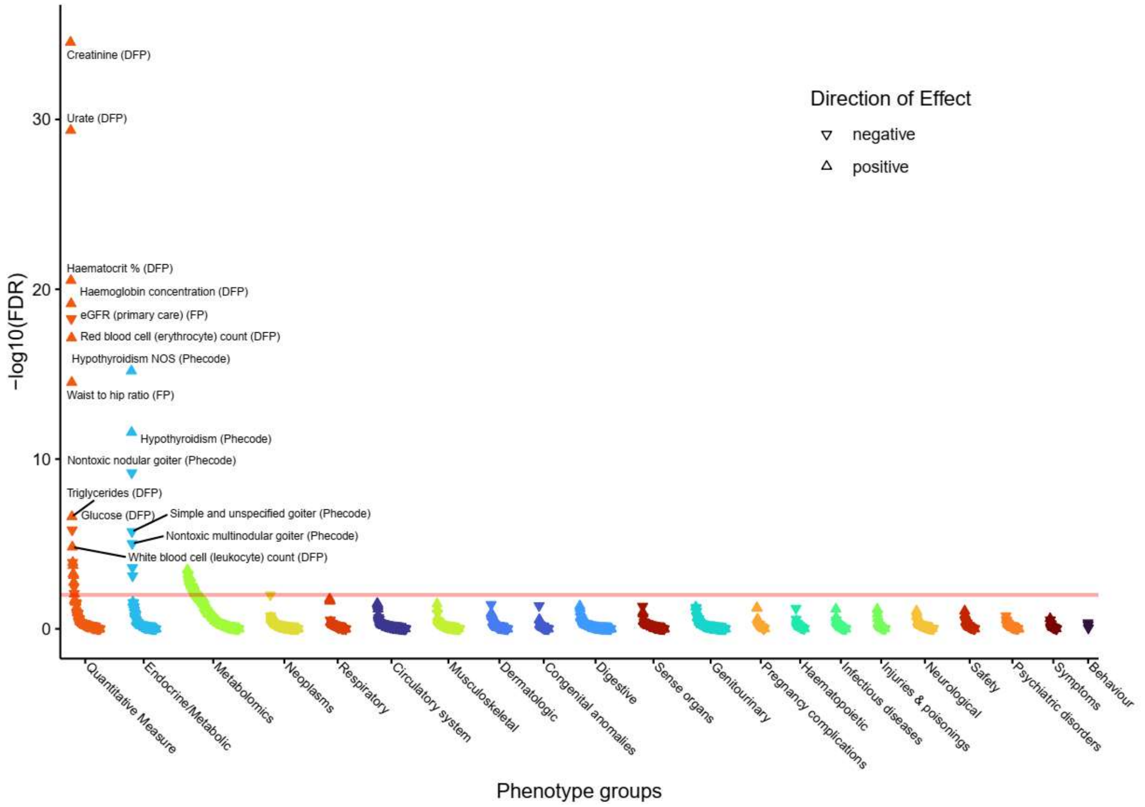

i) Hemostasis pathway.

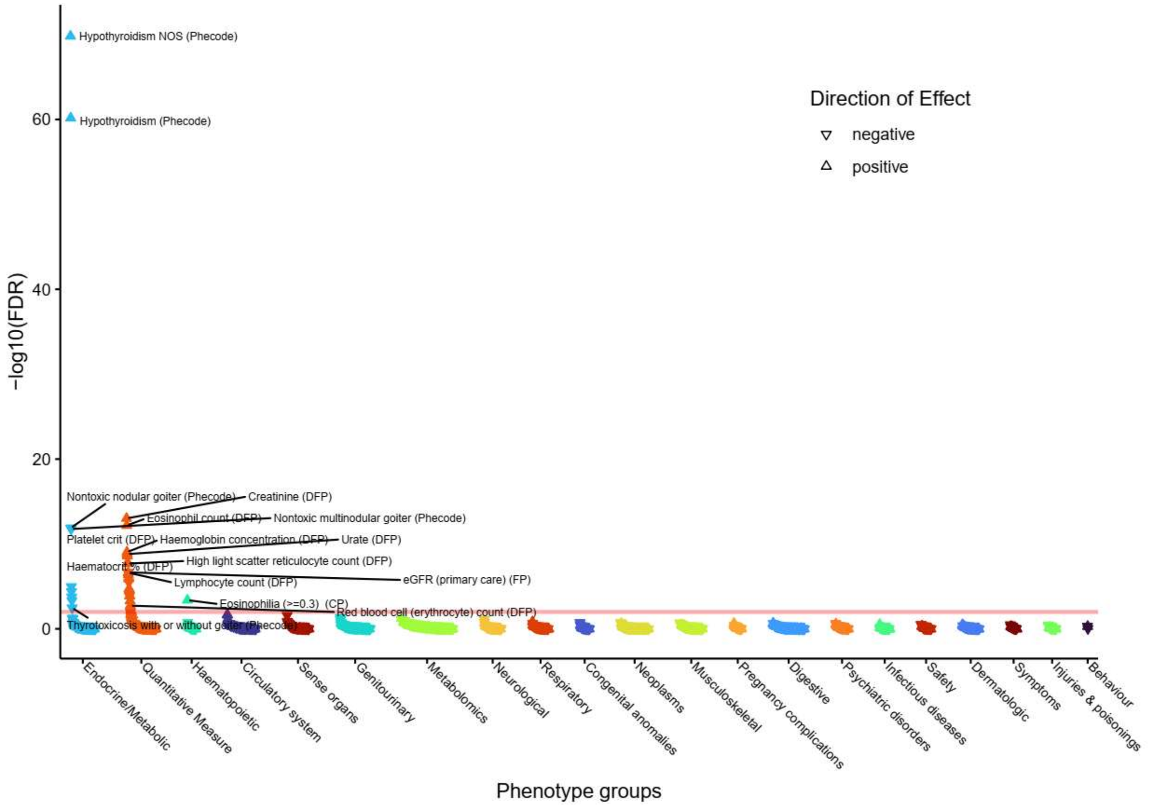

j) Human T-cell leukemia virus 1 infection - Homo sapiens (human) pathway.

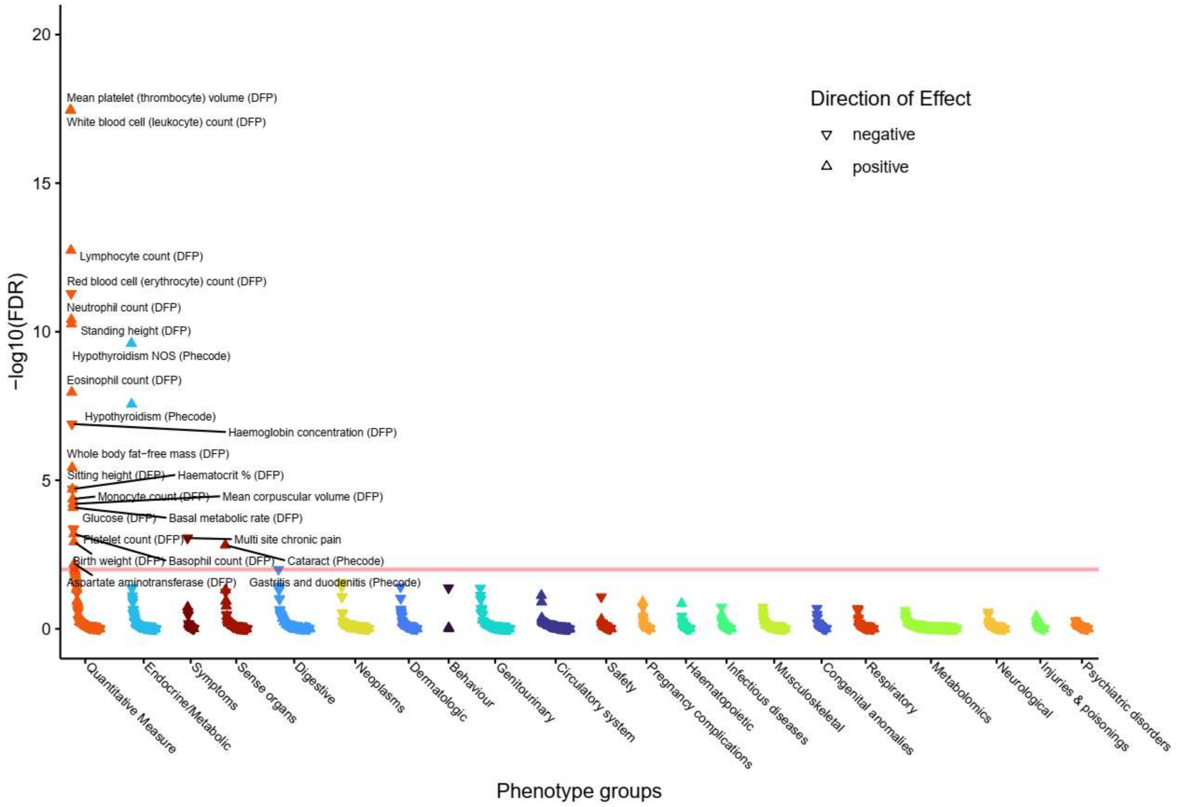

k) Intracellular Signalling Through FSH Receptor and Follicle Stimulating Hormone pathway.

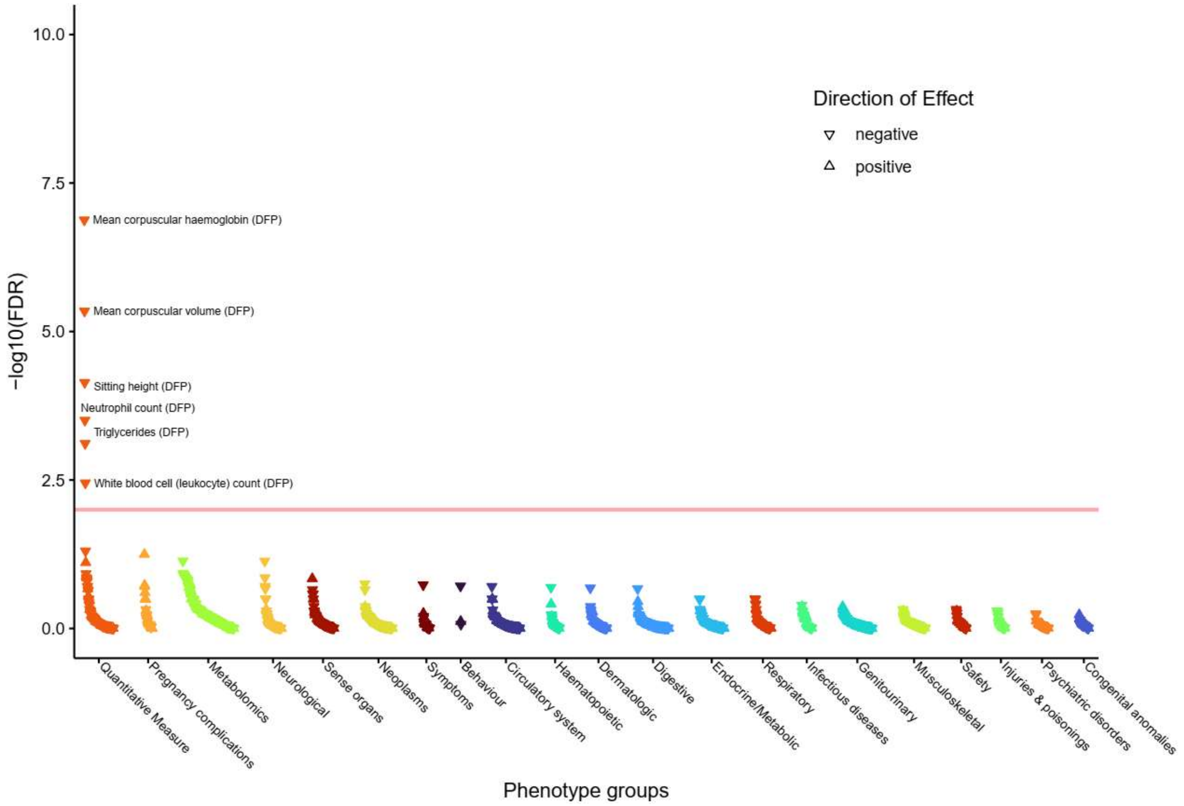

l) Morphine addiction - Homo sapiens (human) pathway.

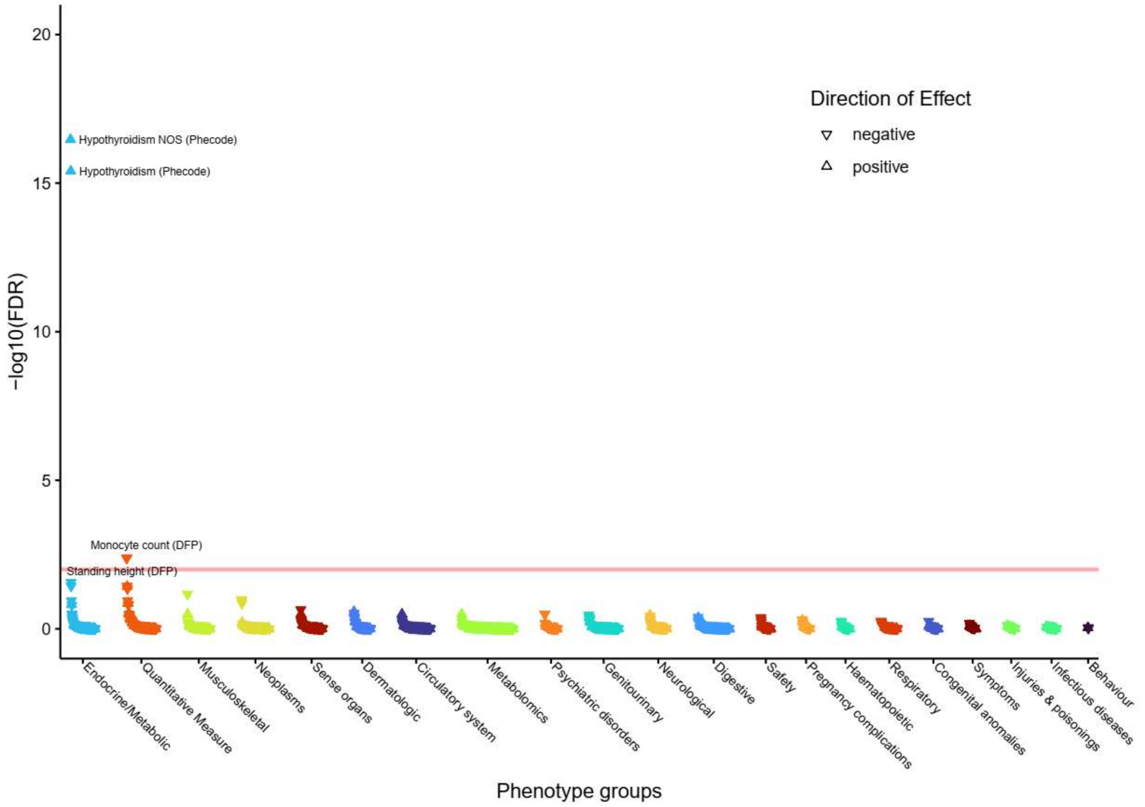

m) Myometrial relaxation and contraction pathways.

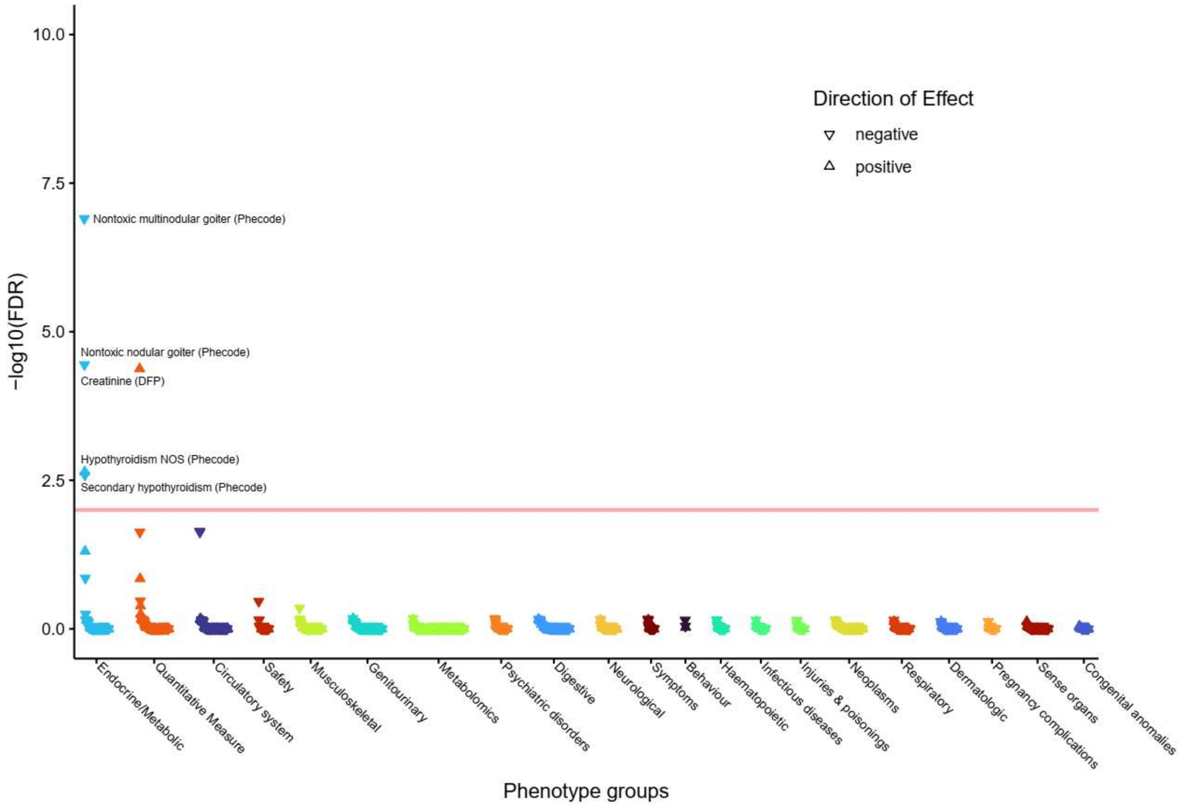

n) Pathways in cancer - Homo sapiens (human) pathway.

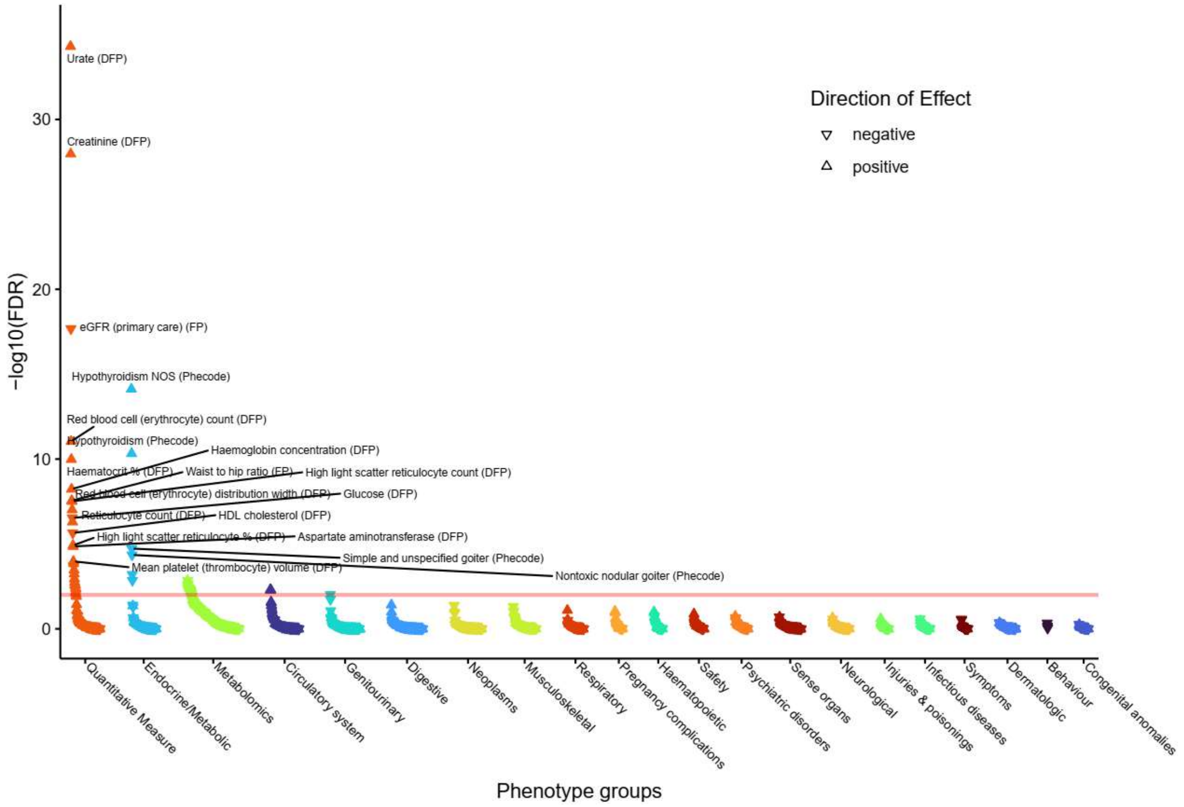

o) Rap1 signaling pathway - Homo sapiens (human) pathway.

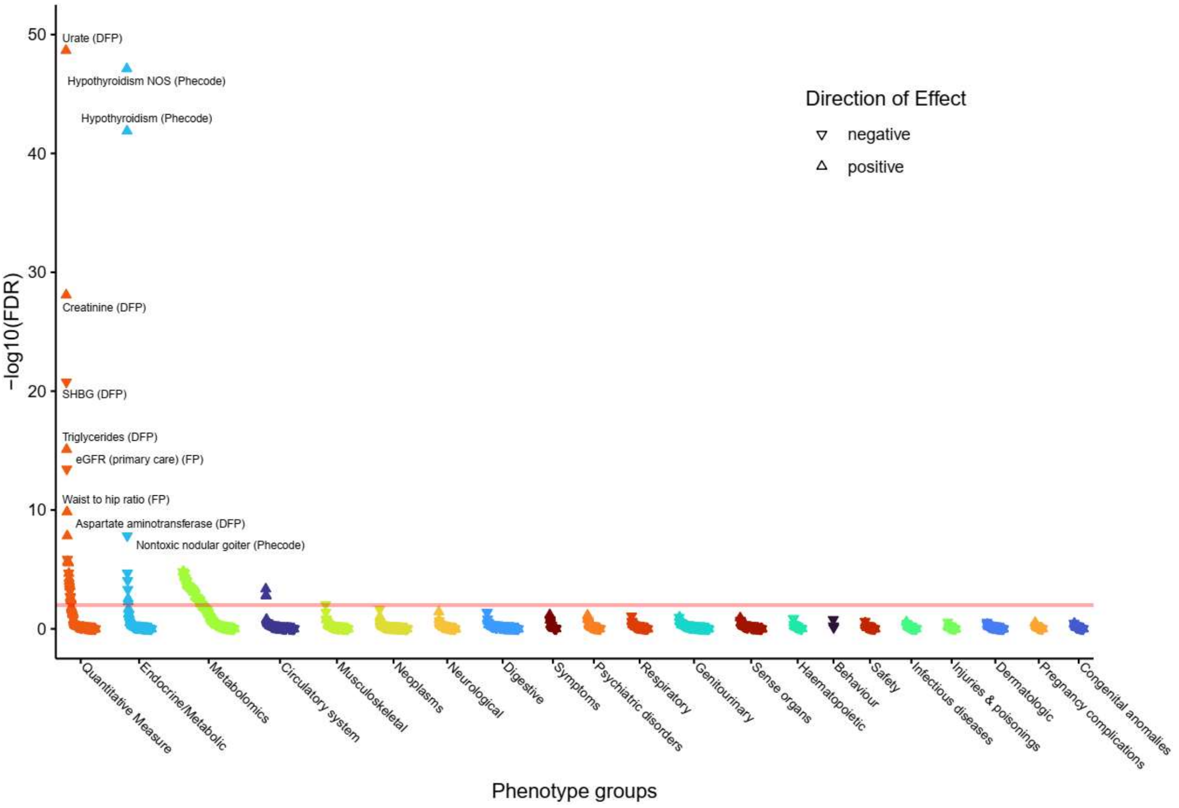

p) Regulation of lipolysis in adipocytes - Homo sapiens (human) pathway.

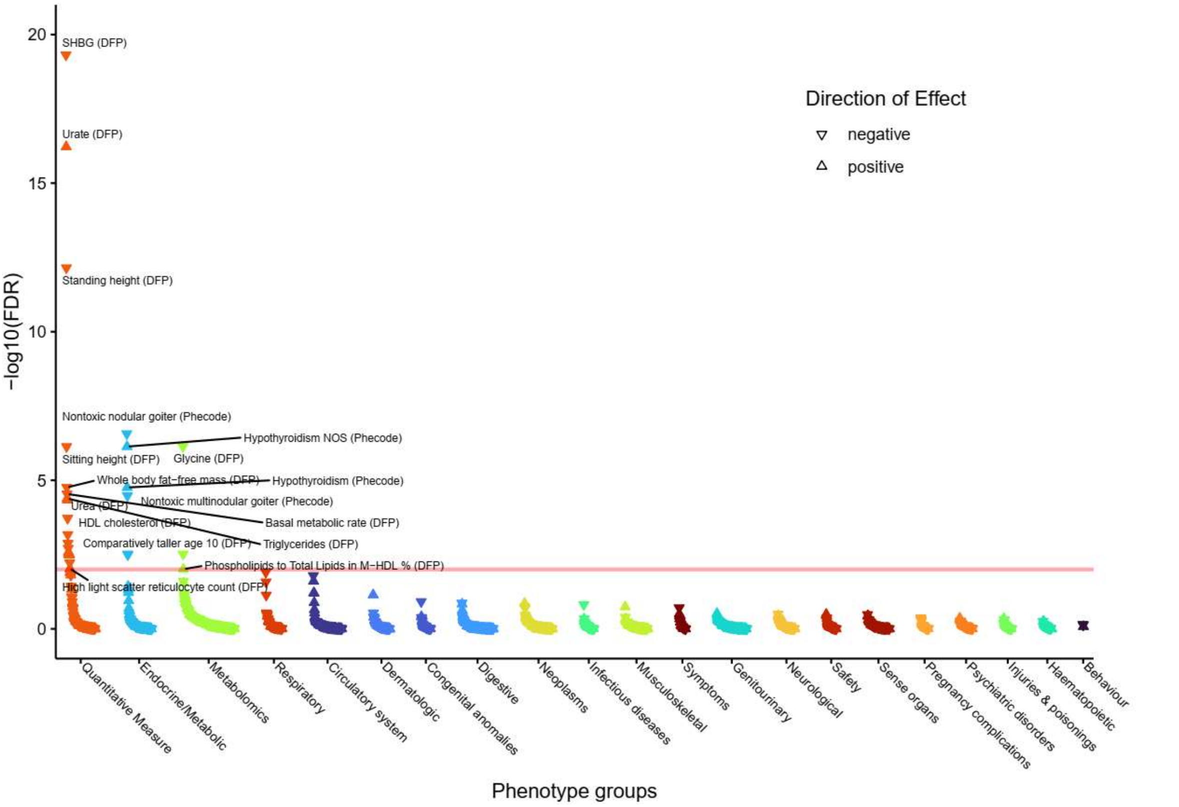

q) Signal Transduction pathway.

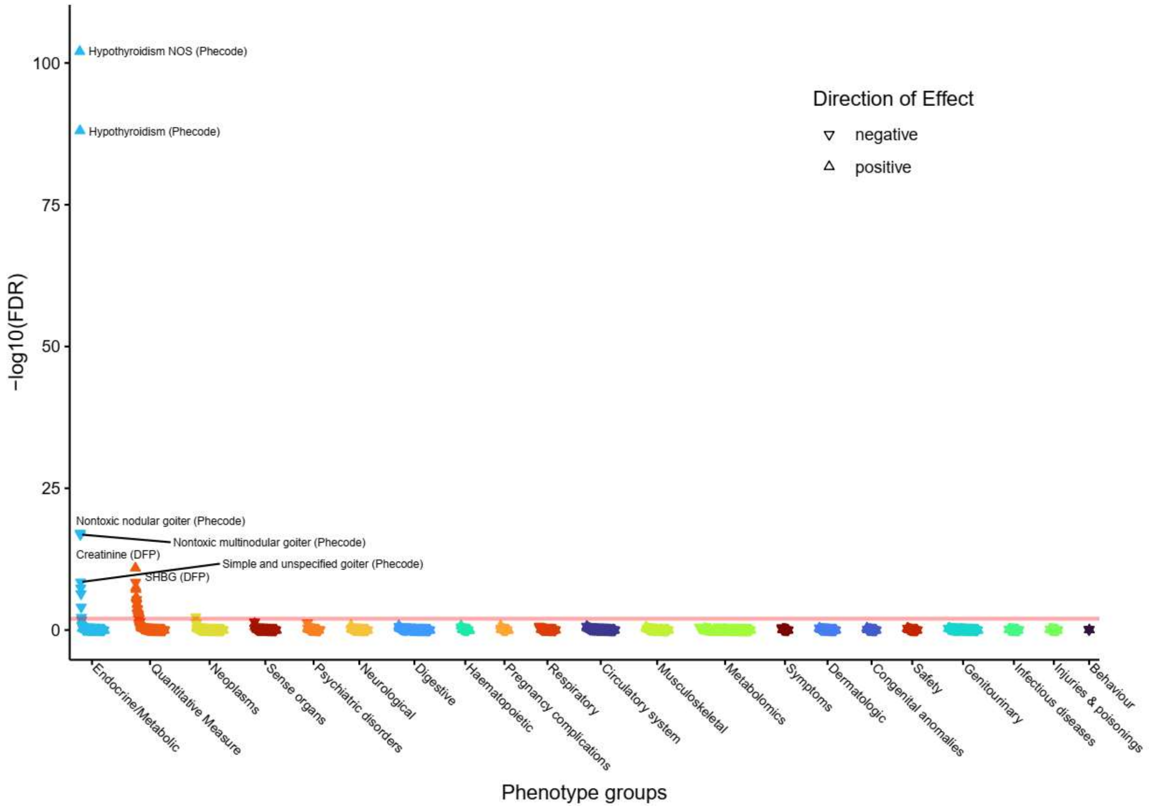

r) Signalling by Receptor Tyrosine Kinases pathway.

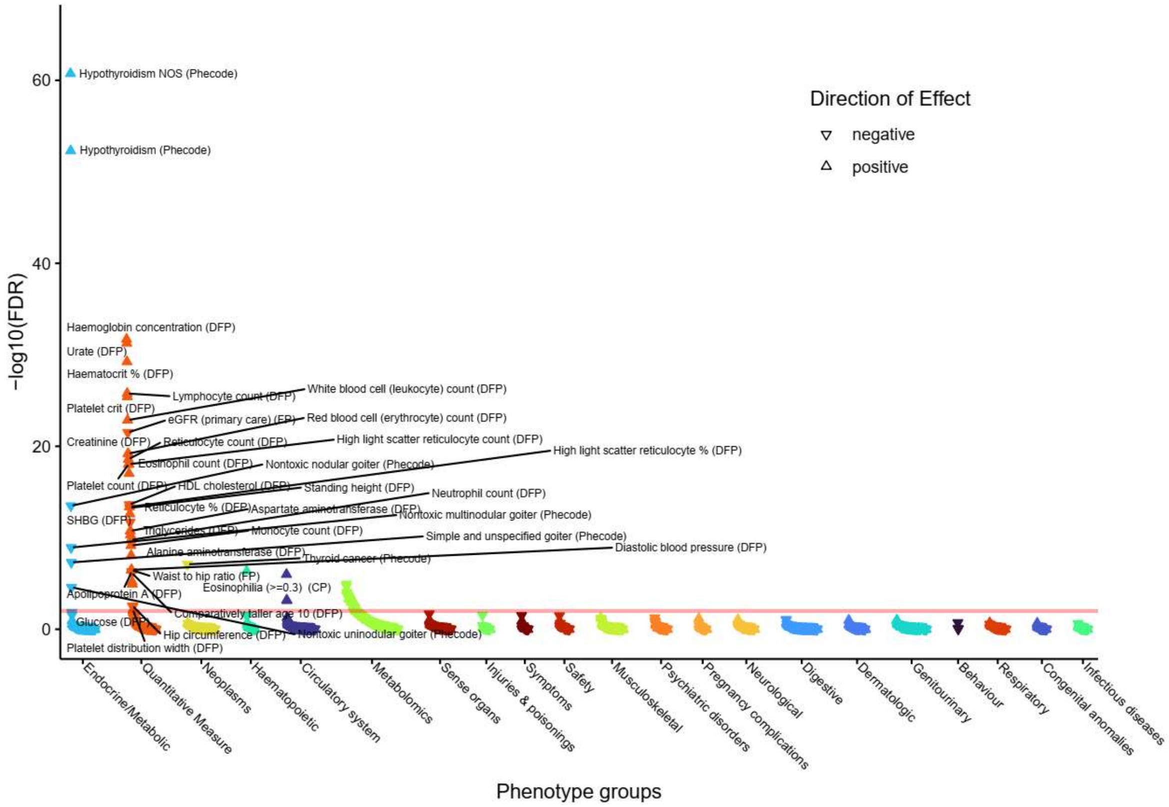

s) Thyroxine (Thyroid Hormone) Production pathway.

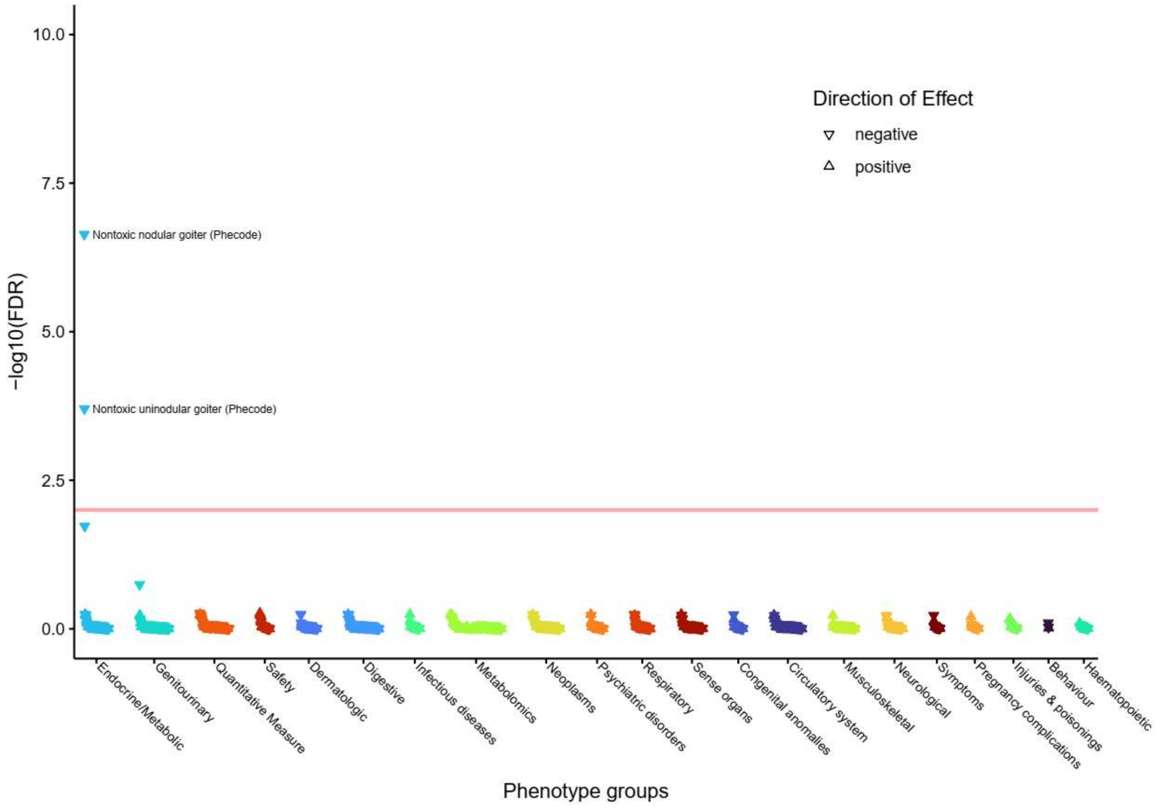

t) Transcriptional regulation by RUNX2 pathway.

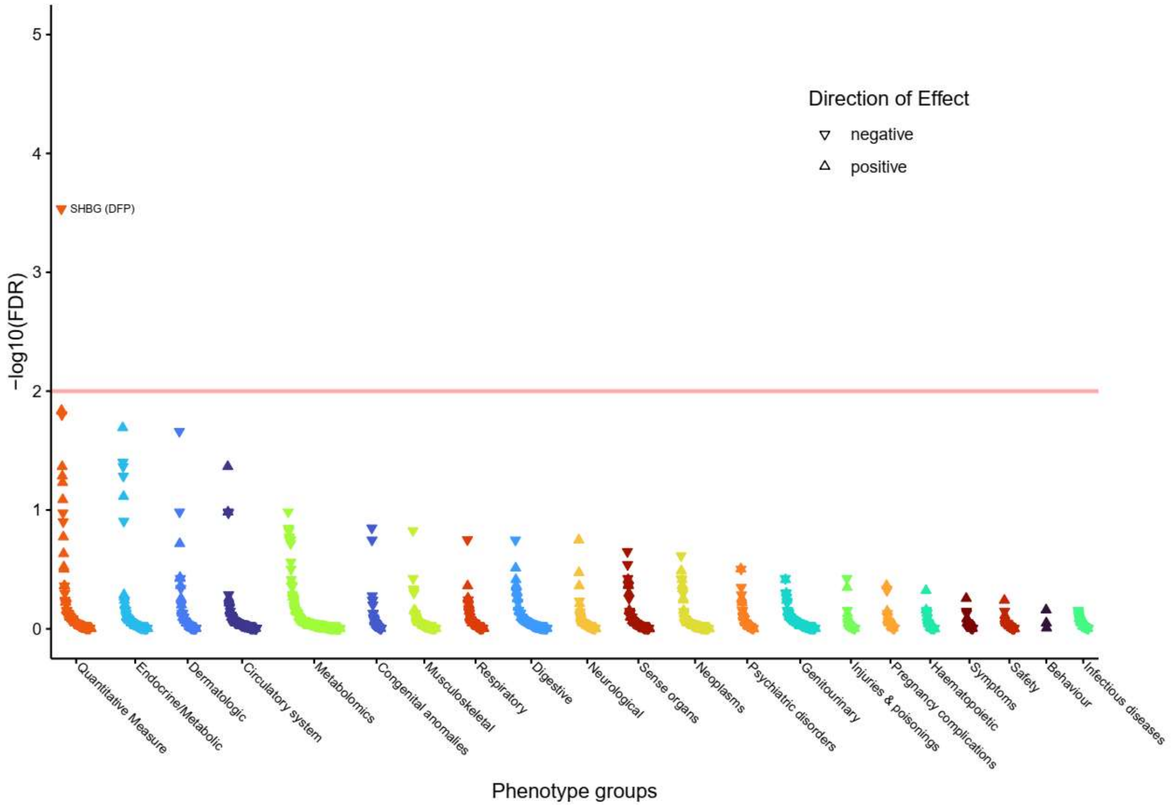

u) **Vegf hypoxia and angiogenesis pathway.**

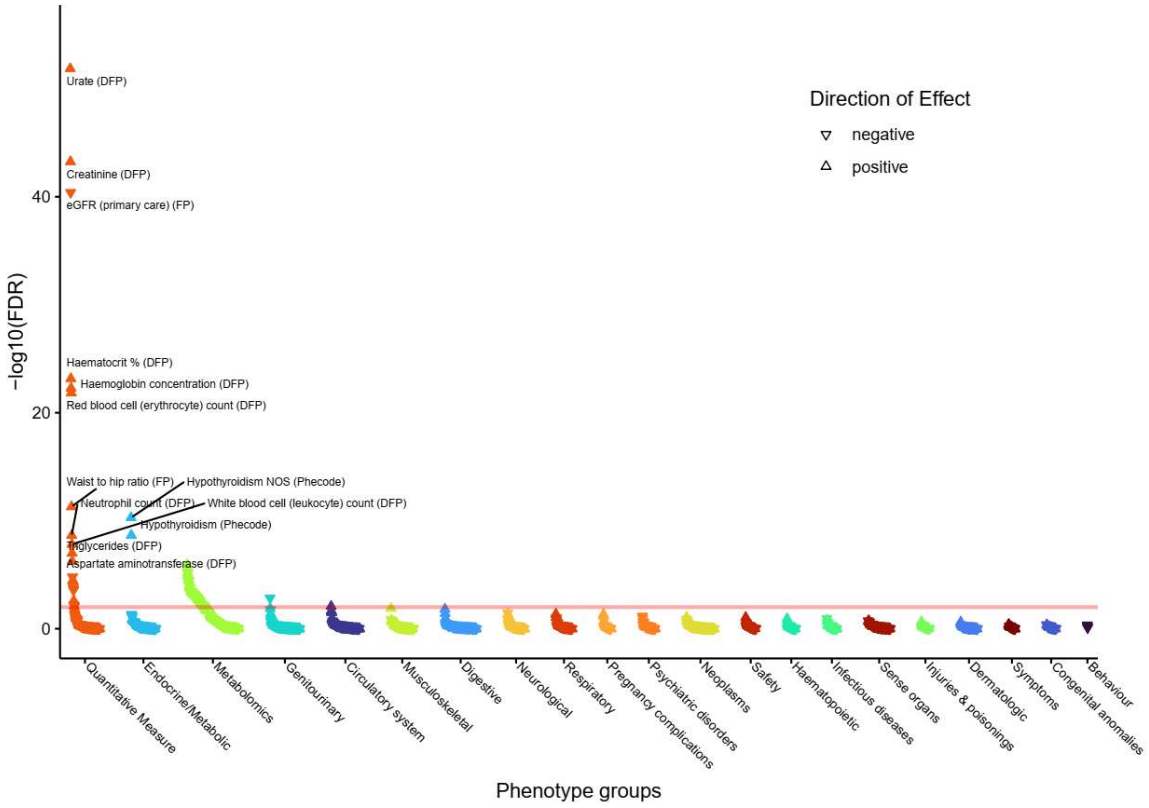

v) **VEGF ligand-receptor interactions pathway.**

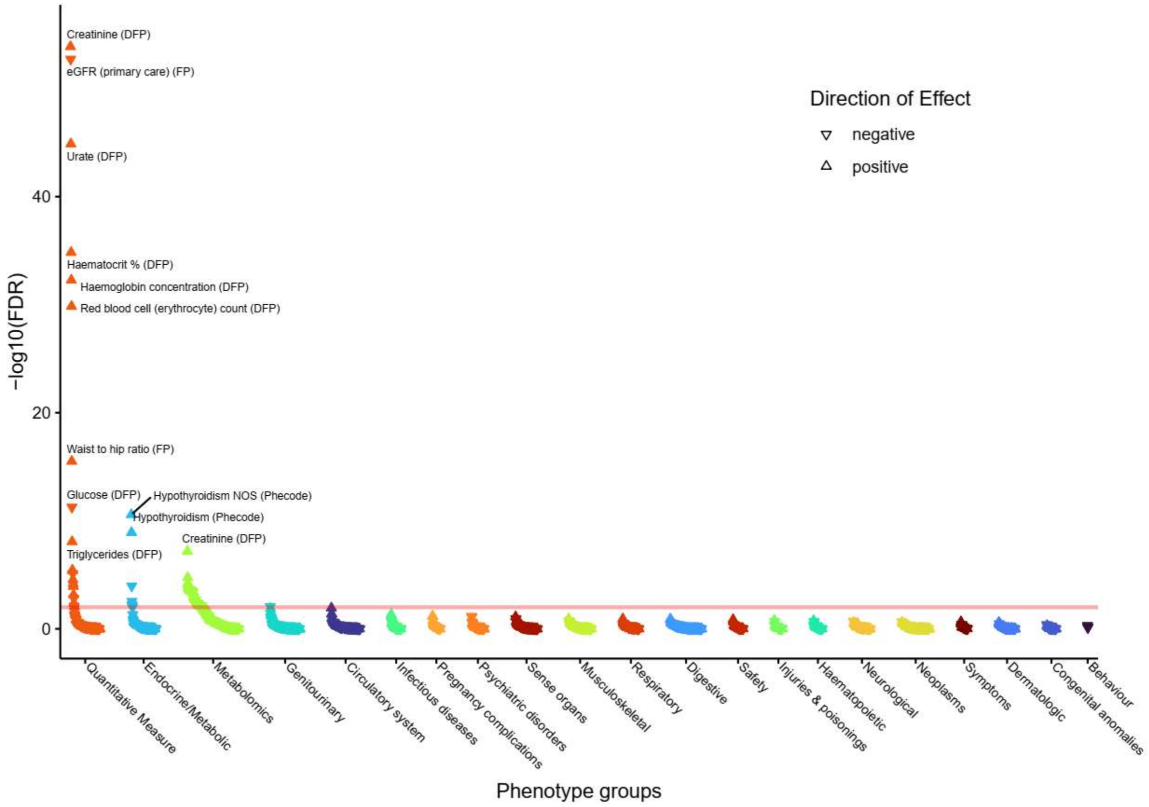

w) VEGFA-VEGFR2 Signaling Pathway.

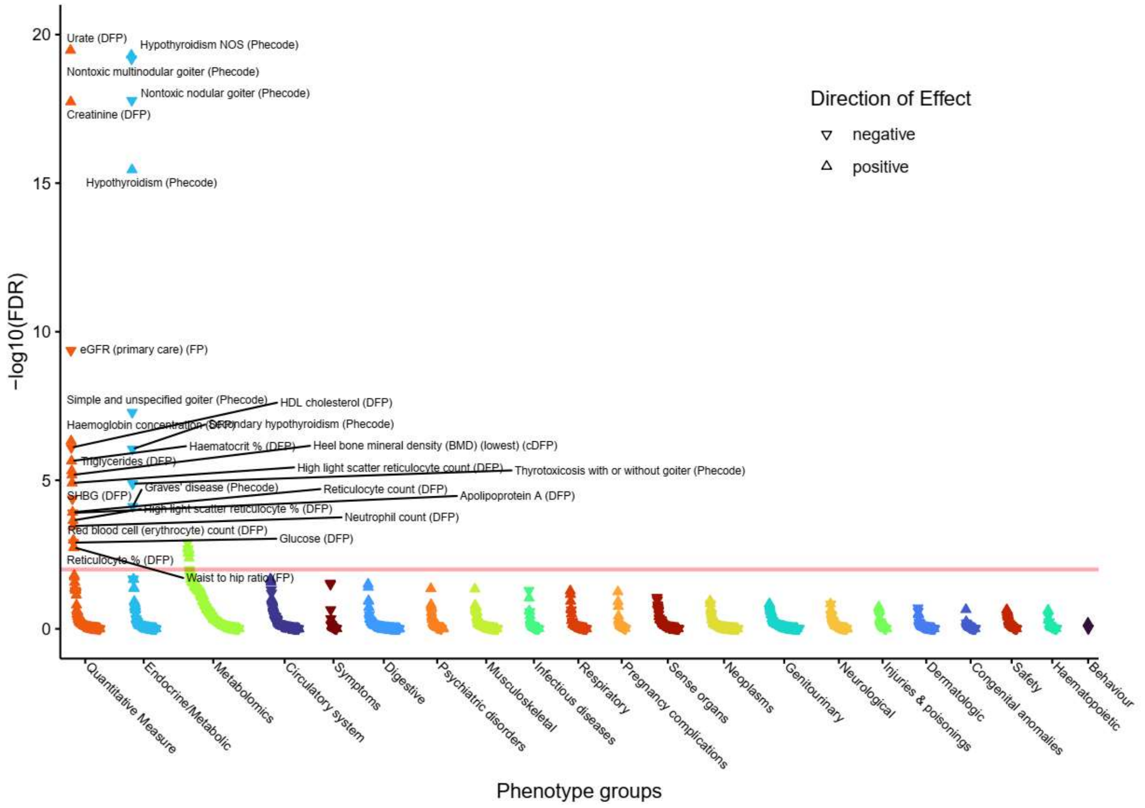

Supplementary Figure 5: Thyroid-stimulating hormone polygenic score PheWAS

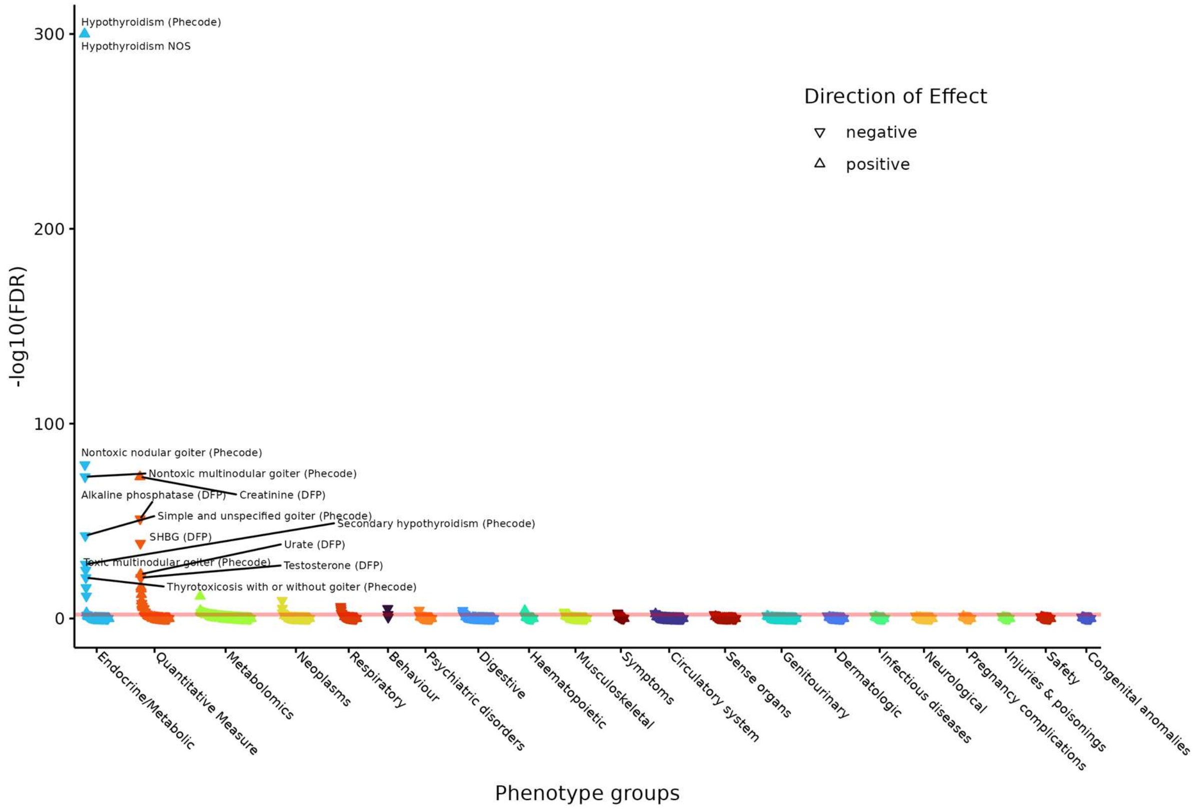

Supplementary Figure 6: ROC curves for TSH PGS, age and sex in prediction of thyroid diseases. a. TSH PGS was used to predict thyroid diseases; b. Age and sex were used to predict thyroid diseases; c. TSH PGS combined with age and sex were used to predict thyroid diseases. A 95% confidence interval of AUC was given in the bracket. Sample size: Hypothyroidism, 29,550 cases and 368,691 controls; Hyperthyroidism, 5,549 cases and 392,692 controls; Thyroid cancer, 624 cases and 358,144 controls; Other thyroid disease, 1,233 cases and 124,515 controls.

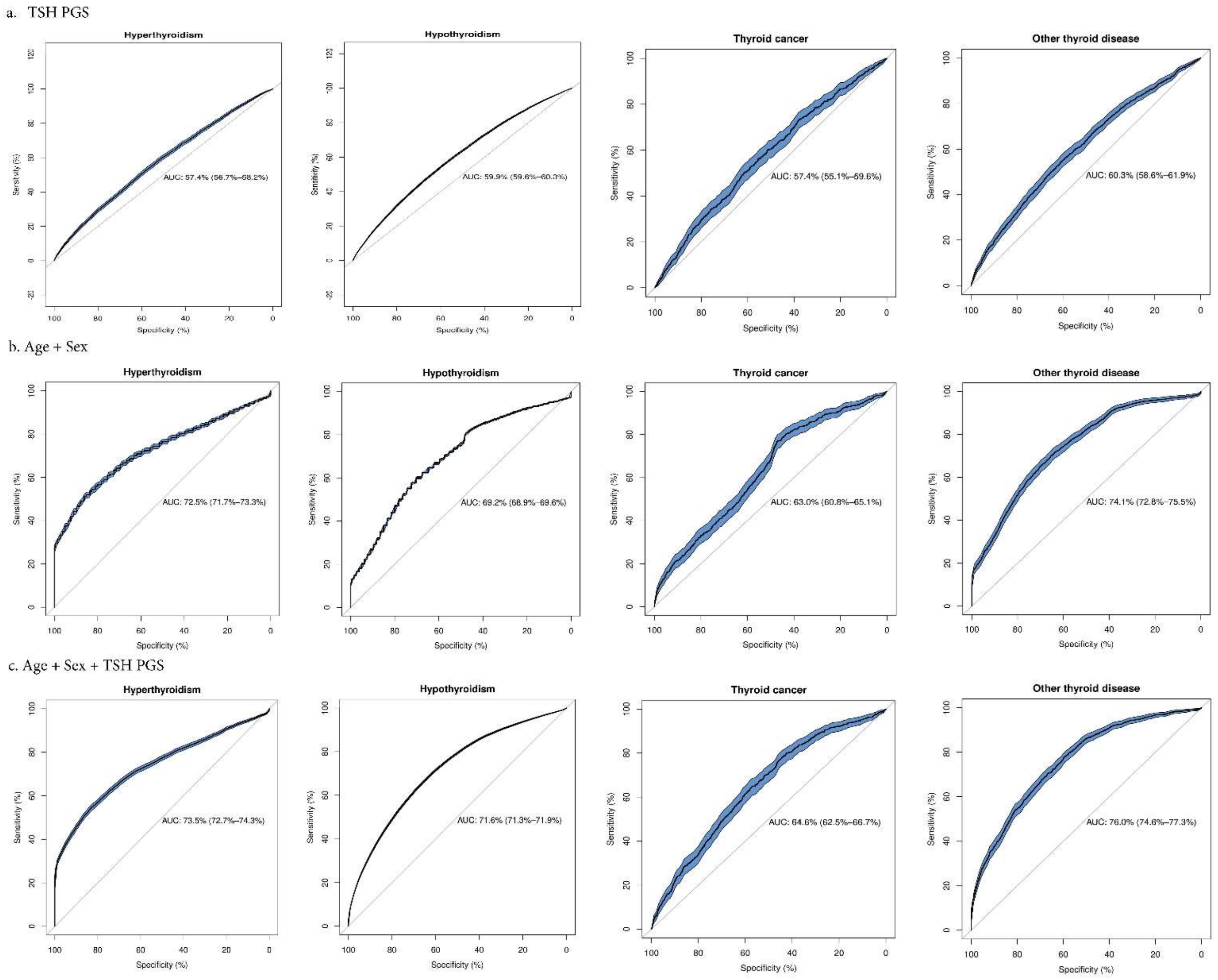

Supplementary Figure 7: Association of TSH PGS deciles with thyroid diseases (sensitivity analysis free of overfitting). The TSH PGS decile analysis for four clinical thyroid phenotypes – (**a, top left**) hypothyroidism, (**b, top right**) hyperthyroidism, **(c, bottom left**) thyroid cancer, and (**d, bottom right**) other thyroid disease. Statistical tests were two-sided, the height of the bars show the point estimate of the effect and whiskers show the 95% CI. OR, odds ratio. The Mann-Kendall test is a test for monotonic trend. Sample size: Hypothyroidism, 23,818 cases and 251,397 controls; Hyperthyroidism, 3,924 cases and 271,114 controls; Thyroid cancer, 445 cases and 244,012 controls; Other thyroid disease, 305 cases and 12,217 controls (Supplementary Data 15).

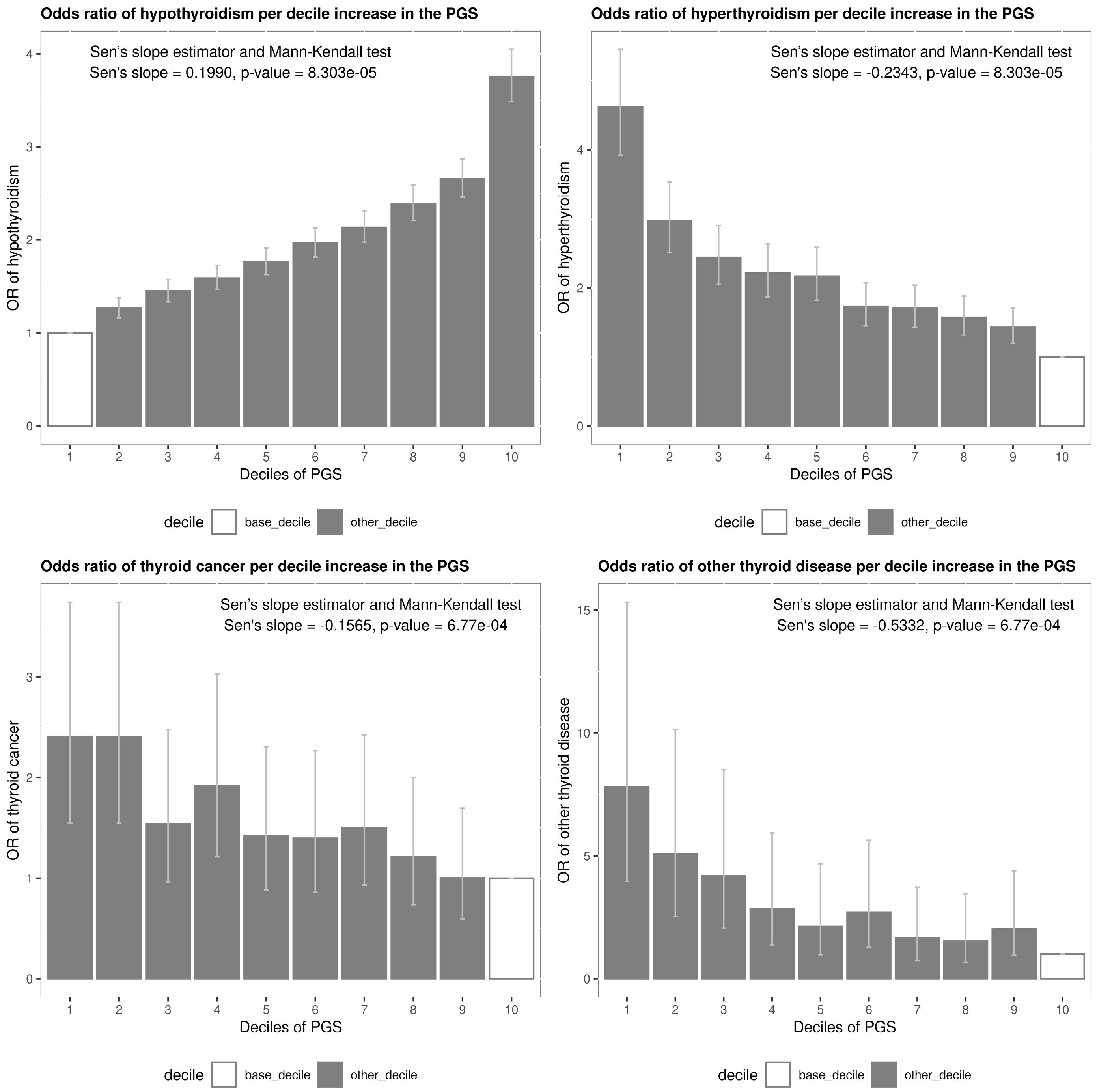

Supplementary Figure 8: Association of TSH PGS with age-of-onset of hypothyroidism and hyperthyroidism (sensitivity analysis free of overfitting). Proportion of hypothyroidism (a, left) and hyperthyroidism (b, right) cases diagnosed by age stratified into lowest (grey), median (blue) and highest (yellow) decile for the TSH PGS. Shaded bands indicate 95% confidence intervals. Sample size: Hypothyroidism, 23,818 cases and 251,397 controls; Hyperthyroidism, 3,924 cases and 271,114 controls. Statistical tests were two-sided.

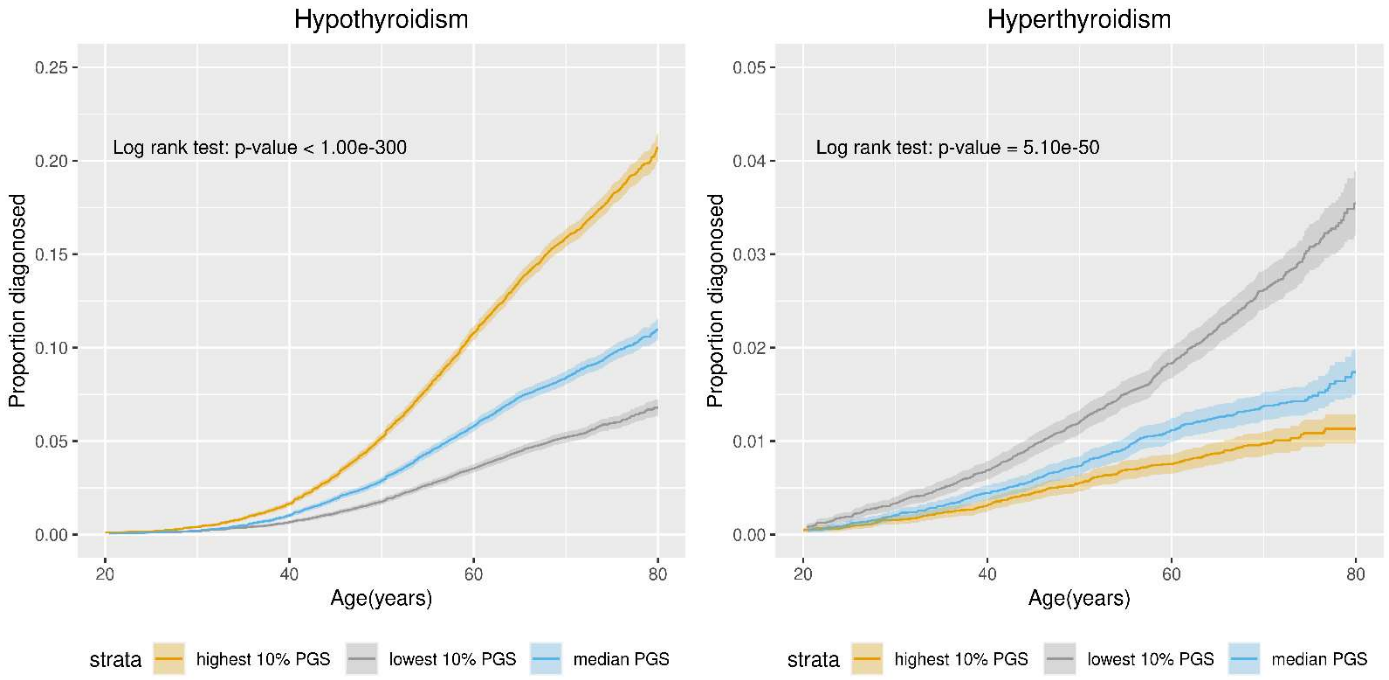

Supplementary Figure 9: ROC curves for TSH PGS, age and sex in prediction of thyroid diseases (sensitivity analysis free of overfitting). a. TSH PGS was used to predict thyroid diseases; b. Age and sex were used to predict thyroid diseases; c. TSH PGS combined with age and sex were used to predict thyroid diseases. A 95% confidence interval of AUC was given in the bracket. Sample size: Hypothyroidism, 23,818 cases and 251,397 controls; Hyperthyroidism, 3,924 cases and 271,114 controls.

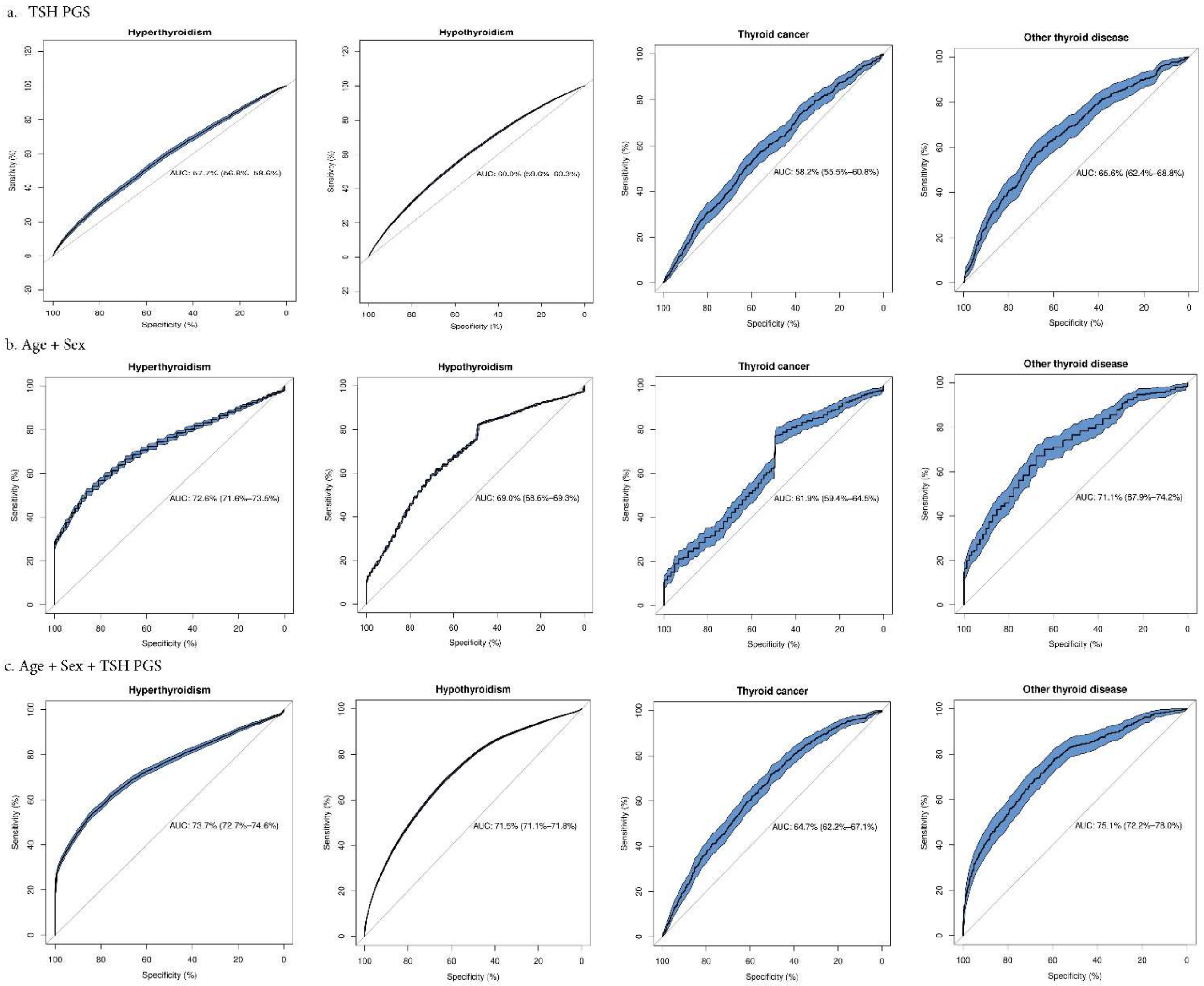

Supplementary Figure 10: Comparison of -log10P values from association analyses for the 260 TSH sentinel variants using the whole range of TSH measures (unrestricted, *y*-axis) versus using the restricted range (*x*-axis) (Supplementary Data 2). Each point represents one sentinel variant, the dashed black line represents the line *y=x*, and the blue solid line represents the line of best fit.

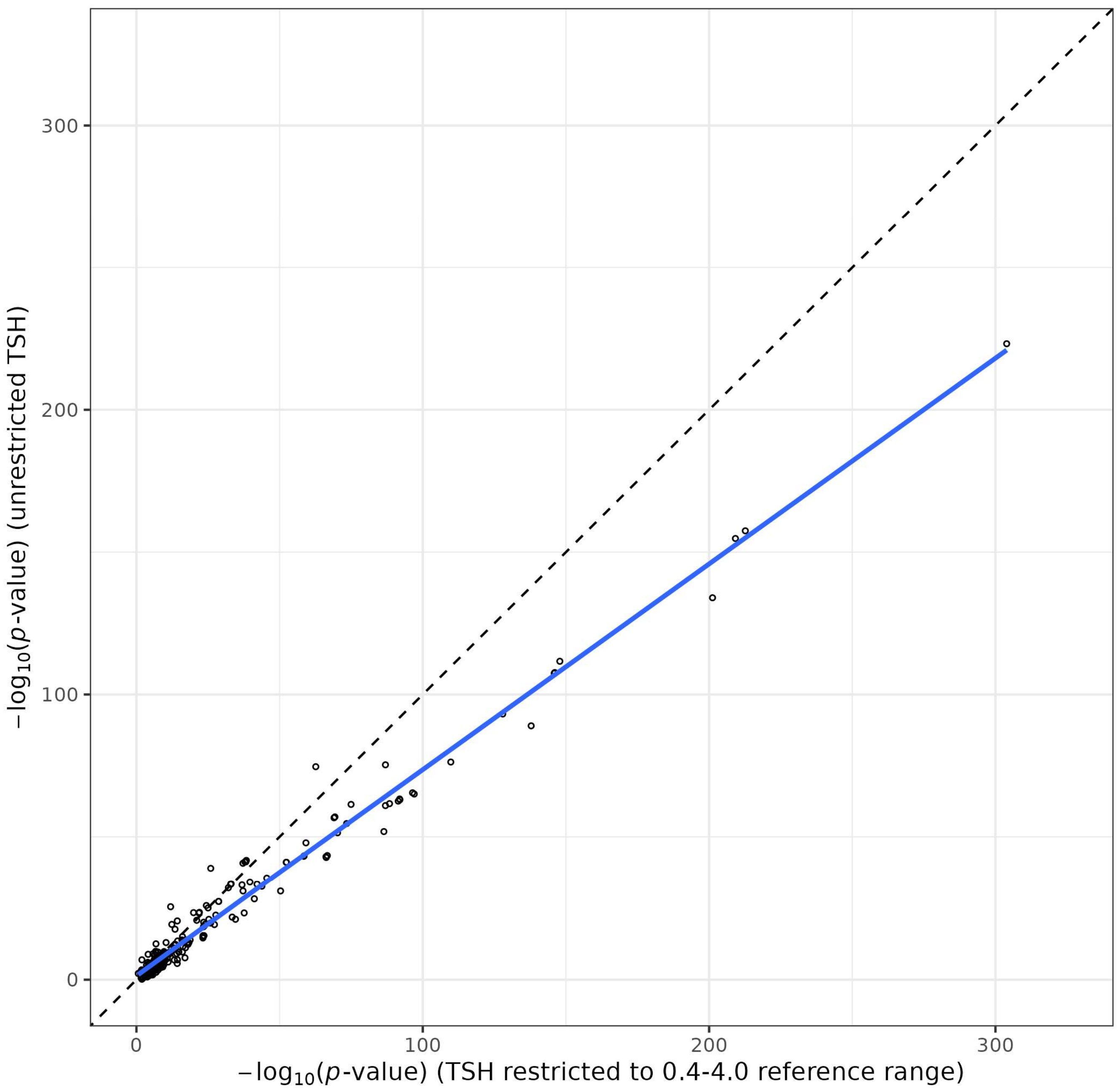

Supplementary Data Legends

Supplementary Data 1: (Excel sheet) Participant demographics across the five studies contributing to the meta-analysis of stages 1 and 2

Supplementary Data 2: (Excel sheet) Results for Stage 1 and resulting sentinel variant selection, Stage 2, and meta-analysis of Stages 1 and 2.
Each row represents the sentinel variant for an independent signal of association: nearest_gene = closest gene to sentinel variant; MarkerName = chromosome, position (b37), followed by the alleles sorted alphabetically; locus + credible_set = unique identifier for a credible set at a genomic locus; method = approach used for fine-mapping; posterior_probability = posterior probability; beta = effect estimate from Stage 1 meta-analysis; se = standard error of effect estimate; p = p-value; direction = direction of effect estimate in the order of UK Biobank/EXCEED/Zhou; sample_size = total sample size for sentinel variant in Stage 1; I2 = estimate of heterogeneity in Stage 1 meta-analysis; novel_signal = is the corresponding sentinel variant not in linkage disequilibrium (R2 <0.2) with any previously reported genetic variants for TSH; novel_locus = does the 2Mb region centred on the corresponding sentinel variant overlap the 2Mb region centred on a previously reported genetic variant for TSH; SNP_reported = ID for previously reported sentinel variant; R2 = extent of linkage disequilibrium between previous sentinel variant and the sentinel variant in the present study; ref = PubMed ID for study where previously reported sentinel variant was identified; n_gene = count of the number of genes implicated by sentinel variant; evidence = sources of evidence implicating gene; annotated_gene = annotated gene; annotated_allele = annotated allele; consequence = consequence of sentinel variant; SIFT = SIFT score; PolyPhen = PolyPhen score; CADD_PHRED = phred-scaled CADD score; topSNP = chromosome, position (b37) followed by the alleles sorted alphabetically of most significant SNP at the locus; beta_stg2 = effect estimate from Stage 2 meta-analysis; se_stg2 = standard error of effect estimate; p_stg2 = p-value; direction_stg2 = direction of effect estimate in the order of Estonian Biobank/Genes & Health; sample_size_stg2 = total sample size for sentinel variant in Stage 2; beta_stg12 = effect estimate from the meta-analysis of Stages 1 and 2; se_stg12 = standard error of effect estimate; p_stg12 = p-value; direction_stg12 = direction of effect estimate in the order of Stage1/Stage2; sample_size_stg12 = total sample size for sentinel variant in the meta-analysis of Stages 1 and 2; beta_UKB = effect estimate in UK Biobank; se_UKB = standard error of effect estimate; p_UKB = p-value; beta_EXCEED = effect estimate in EXCEED; se_EXCEED = standard error of effect estimate; p_EXCEED = p-value; beta_Zhou = effect estimate in Zhou et al; se_Zhou = standard error of effect estimate; p_Zhou = p-value; beta_EstBB = effect estimate in Estonian Biobank; se_EstBB = standard error of effect estimate; p_EstBB = p-value; beta_gh = effect estimate in Genes & Health; se_gh = standard error of effect estimate; p_gh = p-value; beta_sensitivity = effect estimate of sensitivity analysis; se_sensitivity = standard error of effect estimate; pval_sensitivity = p-value

Supplementary Data 3: (Excel sheet) Prioritised genes
List of prioritised genes according to the number of lines of variant-to-gene evidence implicates the gene (n_evidence column). The "Novel" column indicates whether the gene was previously reported. The n_novel_signals lists the number of novel signals implicating the gene.

Supplementary Data 4: (Excel sheet) Previously reported genes
Genes typically implicated by a single criterion and the reference for the previous publication reporting the gene.

Supplementary Data 5: (Excel sheet) Epidemiological associations
Epidemiological associations between the 257 top PIP variants available in UK Biobank and thyroxine (T4) levels or clinical thyroid diseases: hypothyroidism, hyperthyroidism, thyroid cancer, and other non-cancer thyroid diseases. Also shown are epidemiological associations with hypothyroidism due to medication defined using phecode 244.1 - these associations were only assessed in genetic variants implicating genes that met two or more variant-to-gene criteria.

Supplementary Data 6: (Excel sheet) Single-variant PheWAS associations
Phenome-wide association study (PheWAS) results for select TSH sentinel variants for associations at FDR <1% (European ancestry). N_ID = Number of participants in the analysis, FDR = false discovery rate, P = p value, OR = odds ratio, L95 = lower boundary of the 95% confidence interval, L95 = upper boundary of the 95% confidence interval, MAF = minor allele frequency, MAC = minor allele count, MAC_cases = minor allele count in cases, MAC_controls = minor allelecount in controls. Z_T_STAT = the Z or T STAT output from Plink2, SE = standard error. The P value is the

P value from Plink2 for association for a linear model or logistic regression for the phenotype after adjusting for age, sex and first 10 principal components.

Supplementary Data 7: (Excel sheet) Variants selected at each gene for look up of epidemiological associations
For each gene the variant with the most significant association across either 6 or 7 binary traits is shown.

Supplementary Data 8: (Excel sheet) Druggability

Drug Gene Interaction Database (DGIDB) results. Columns:

- Drug; drug/compound name,
- ChEMBL_ID; drug/compound identification number from ChEMBL,
- Gene; mapped gene(s),
- Gene_source; line(s) of evidence for each mapped gene, and signal implicated (including the associated lung function trait),
- Indication(Phase); drug indication phase. Phase 1: Testing of drug on healthy volunteers for dose-ranging; Phase 2: Testing of drug on patients to assess efficacy and safety; Phase 3: Testing of drug on patients to assess efficacy, effectiveness and safety; and Phase 4: Approval of drug and post-marketing surveillance,
- Thyroid_related: whether the drug is used to for the treatment of thyroid conditions.
- Cancer_related: whether the drug is used to for the treatment of some form of cancer.

Supplementary Data 9: (Excel sheet) ConsensuspathDB results
ConsensuspathDB pathways enriched (FDR <5%) for genes from our 112 with at least 2 lines of evidence for being causal. The P value and FDR for enrichment of genes in pathways are those returned by ConsensuspathDB{Herwig, 2016 #78}.

Supplementary Data 10: (Excel sheet) Pathway-based PheWAS results
Results of 29 pathway-specific PheWAS for FDR <1%: FDR = false discovery rate, P = p value, OR = odds ratio, L95 = lower boundary of the 95% confidence interval, L95 = upper boundary of the 95% confidence interval.

Supplementary Data 11: (Excel sheet) TSH polygenic risk score PheWAS results
Results of TSH PGS PheWAS for FDR <1%: FDR = false discovery rate, P = p value, OR = odds ratio, L95 = lower boundary of the 95% confidence interval, L95 = upper boundary of the 95% confidence interval.

Supplementary Data 12: (Excel sheet) Association results of TSH and free T4 with TSH PGS in ancestry groups in UK Biobank

Supplementary Data 13: (Excel sheet) Association results of diseases with TSH PGS in ancestry groups in UK Biobank

Supplementary Data 14: (Excel sheet) Association results of diseases with TSH PGS in ancestry groups in UK Biobank (winner's curse free)

Supplementary Data 15: (Excel sheet) 95% credible sets
All variants in the 95% credible sets are shown. The posterior probability (PIP column) returned from Polyfun Susie or Wakefield (method column). Allele1 is the coded/effect allele.

Supplementary Data 16: (Excel sheet) Clinical codes used to define thyroid-stimulating hormone (TSH), free T4, hypo- and hyperthyroidism, other thyroid diseases, and thyroid cancer

Supplementary Data 17: (Excel sheet) 95% credible sets

Supplementary Data 18: (Excel sheet) FUMA eQTL look up results
Look up of eQTL results from the GTEx v8 (thyroid, hypothalamus and pituitary tissues) and eQTLGen (blood, cis- and trans-eQTLs) datasets for variants in our 95% credible sets.

Supplementary Data 19: (Excel sheet) Polygenic Priority Score (PoPS) results - 500KB window
Polygenic Priority Score (PoPS) results using a +/-250KB window

Supplementary Data 20: (Excel sheet) Genes near our signals associated with rare Mendelian respiratory diseases
34 genes associated with a rare Mendelian respiratory disease were implicated.

Supplementary Data 21: (Excel sheet) Mouse ortholog genes near our signals associated with a respiratory disease
6 genes were implicated

Supplementary Data 22: (Excel sheet) Look up of UK Biobank WES variants
Look up of UK Biobank WES variants within +/-500kb of TSH sentinels.

Supplementary Data 23: (Excel sheet) Putative causal variants
Variants from credible sets annotated as missense/damaging/deleterious and with a PIP>50%
