# Supplementary material for: Genome-wide association study of thyroid-stimulating hormone highlights new genes, pathways and associations with thyroid disease
Source: Nat Commun. 2023 Oct 23;14:6713. doi: 10.1038/s41467-023-42284-5 (PMC10593800; doi:10.1038/s41467-023-42284-5)
Supplement: Supplementary file 3 — Description of Additional Supplementary Files [file 41467_2023_42284_MOESM3_ESM.pdf]

Description of Additional Supplementary Files

Supplementary Data 1: Participant demographics across the five studies contributing to the meta-analysis of stages 1 and 2

Supplementary Data 2: Results for Stage 1 and resulting sentinel variant selection, Stage 2, and meta-analysis of Stages 1 and 2.

Supplementary Data 3: Prioritised genes

Supplementary Data 4: Previously reported genes

Supplementary Data 5: Epidemiological associations

Supplementary Data 6: Single-variant PheWAS associations

Supplementary Data 7: Variants selected at each gene for look up of epidemiological associations

Supplementary Data 8: Druggability

Supplementary Data 9: ConsensuspathDB results

Supplementary Data 10: Pathway-based PheWAS results

Supplementary Data 11: TSH polygenic risk score PheWAS results

Supplementary Data 12: Association results of TSH and free T4 with TSH PGS in ancestry groups in UK Biobank

Supplementary Data 13: Association results of diseases with TSH PGS in ancestry groups in UK Biobank

Supplementary Data 14: Association results of diseases with TSH PGS in ancestry groups in UK Biobank (winner's curse free)

Supplementary Data 15: 95% credible sets

Supplementary Data 16: Clinical codes used to define thyroid-stimulating hormone (TSH), free T4, hypo- and hyperthyroidism, other thyroid diseases, and thyroid cancer

Supplementary Data 17: 95% credible sets

Supplementary Data 18: FUMA eQTL look up results

Supplementary Data 19: Polygenic Priority Score (PoPS) results - 500KB window

Supplementary Data 20: Genes near our signals associated with rare Mendelian respiratory diseases

Supplementary Data 21: Mouse ortholog genes near our signals associated with a respiratory disease

Supplementary Data 22: Look up of UK Biobank WES variants

Supplementary Data 23: Putative causal variants
