# Supplementary material for: Genome-wide association study of thyroid-stimulating hormone highlights new genes, pathways and associations with thyroid disease
Source: Nat Commun. 2023 Oct 23;14:6713. doi: 10.1038/s41467-023-42284-5 (PMC10593800; doi:10.1038/s41467-023-42284-5)
Supplement: Supplementary file 5 — Reporting Summary [file 41467_2023_42284_MOESM5_ESM.pdf]

nature portfolio

Corresponding author(s): Alexander T Williams and Catherine John

Last updated by author(s): 25/8/23

Reporting Summary

Nature Portfolio wishes to improve the reproducibility of the work that we publish. This form provides structure for consistency and transparency in reporting. For further information on Nature Portfolio policies, see our Editorial Policies and the Editorial Policy Checklist.

Statistics

For all statistical analyses, confirm that the following items are present in the figure legend, table legend, main text, or Methods section.

n/a	Confirmed	
☐	☒	The exact sample size (*n*) for each experimental group/condition, given as a discrete number and unit of measurement
☒	☐	A statement on whether measurements were taken from distinct samples or whether the same sample was measured repeatedly
☐	☒	The statistical test(s) used AND whether they are one- or two-sided<br>*Only common tests should be described solely by name; describe more complex techniques in the Methods section.*
☐	☒	A description of all covariates tested
☐	☒	A description of any assumptions or corrections, such as tests of normality and adjustment for multiple comparisons
☐	☒	A full description of the statistical parameters including central tendency (e.g. means) or other basic estimates (e.g. regression coefficient) AND variation (e.g. standard deviation) or associated estimates of uncertainty (e.g. confidence intervals)
☐	☒	For null hypothesis testing, the test statistic (e.g. *F*, *t*, *r*) with confidence intervals, effect sizes, degrees of freedom and *P* value noted<br>*Give P values as exact values whenever suitable.*
☐	☒	For Bayesian analysis, information on the choice of priors and Markov chain Monte Carlo settings
☒	☐	For hierarchical and complex designs, identification of the appropriate level for tests and full reporting of outcomes
☐	☒	Estimates of effect sizes (e.g. Cohen's *d*, Pearson's *r*), indicating how they were calculated

Our web collection on statistics for biologists contains articles on many of the points above.

Software and code

Policy information about availability of computer code

Data collection	None
Data analysis	R 4.1.0, Plink v1.9 & Plink v2.0, LDSC v1.0.1, METAL v2018-08-28, LiftOver v2011-09-27, PolyFun v2022-01-27, susieR v0.12.27, FUMA v1.5.4, PRS-CS-auto v1.0.0, DeepPheWAS v0.2.9

For manuscripts utilizing custom algorithms or software that are central to the research but not yet described in published literature, software must be made available to editors and reviewers. We strongly encourage code deposition in a community repository (e.g. GitHub). See the Nature Portfolio guidelines for submitting code & software for further information.

Data

Policy information about availability of data

All manuscripts must include a data availability statement. This statement should provide the following information, where applicable:

- Accession codes, unique identifiers, or web links for publicly available datasets
- A description of any restrictions on data availability
- For clinical datasets or third party data, please ensure that the statement adheres to our policy

Access to UK Biobank (https://www.ukbiobank.ac.uk/), Estonian Biobank (https://genomics.ut.ee/en/content/estonian-biobank), Genes & Health (https://www.genesandhealth.org/) and EXCEED (https://exceed.org.uk/) datasets is available to bona fide researchers upon application (in accordance with the terms of ethical approval and participant consent). The genome-wide summary statistics from Zhou et al. (2020) can be freely downloaded from http://csg.sph.umich.edu/

willer/public/TSH2020/.

Genome-wide summary statistics will be made publicly available via the EMBL-EBI GWAS Catalog.

URLs for other external datasets are as follows: GTEx (https://www.gtexportal.org/home/), eQTLGen (https://www.eqtlgen.org/), FUMA (https://fuma.ctglab.nl/), deCODE (https://www.decode.com/), SCALLOP (https://olink.com/our-community/scallop/), ConsensusPathDB (http://cpdb.molgen.mpg.de/), Ensembl Variant Effect Predictor (https://www.ensembl.org/info/docs/tools/vep/index.html), the Drug Gene Interaction Database (https://www.dgidb.org/), PoPS (https://github.com/FinucaneLab/pops), Orphanet (https://www.orpha.net/), the International Mouse Phenotyping Consortium (https://www.mousephenotype.org/), PolyFun (https://github.com/omerwe/polyfun), PubMed (https://pubmed.ncbi.nlm.nih.gov/), EMBL-EBI GWAS Catalog (https://www.ebi.ac.uk/gwas/), DEPICT (https://github.com/perslab/depict), AZ PheWAS portal (https://azphewas.com/), genome assembly GRCh37 (https://www.ncbi.nlm.nih.gov/datasets/genome/GCF_000001405.13/).

Human research participants

Policy information about studies involving human research participants and Sex and Gender in Research.

Reporting on sex and gender

Sex (self-reported or from health record data depending on the cohort) was used as a co-variate. In UK Biobank, EXCEED, Genes & Health and Estonian Biobank there were 134,185 females and 89,704 males.

Population characteristics

Genotype information, age, sex and principal components of ancestry were used in the genome-wide association analysis. Age and sex characteristics of the contributing cohorts are given in Supplementary Table 1. Thyroid disease characteristics relevant to the polygenic score analyses are given in Supplementary Tables 14 and 15.

We studied 124,358 individuals from UK Biobank. Of these, 67,808 (54.5%) were female, 57,173 (46.5%) were smokers, 5258 (4.4%) had been diagnosed as hypothyroid, and 1529 (1.3%) had been diagnosed as hyperthyroid. The mean (standard deviation) age was 56.0 (8.8) and the mean (standard deviation) TSH measurement was 1.80 (0.79).
We studied 3034 individuals from the EXCEED cohort. Of these, 1753 (57.8%) were female, 1421 (46.8%) were smokers, 110 (3.6%) had been diagnosed as hypothyroid, and 21 (0.7%) had been diagnosed as hyperthyroid. The mean age was 51.5 (9.1) and the mean TSH measurement was 1.84 (0.78).

We studied 63,326 individuals from the Estonian Biobank. Of these, 45,589 (72.0%) were female, 21,742 (44.8%) were smokers, 6893 (10.9%) had been diagnosed as hypothyroid, and 3984 (6.3%) had been diagnosed as hyperthyroid. The mean age was 53.1 (17.1) and the mean TSH measurement was 1.65 (0.83).

Finally, we studied 33,171 individuals from Genes & Health. Of these, 19,035 (57.4%) were female, 9358 (28.2%) were smokers, 2596 (7.3%) had been diagnosed as hypothyroid, and 691 (2.1%) had been diagnosed as hyperthyroid. The mean age was 36.1 (12.7) and the mean TSH measurement was 2.05 (1.49).

Overall participant demographics were not provided in the Zhou et al. study (PMID: 32769997).

Recruitment

Recruitment to the cohorts is fully described in publications cited in the manuscript.

Ethics oversight

The UK Biobank genetic and phenotypic data were analysed under UK Biobank Application 43027. UK Biobank has ethical approval from the UK National Health Service (NHS) National Research Ethics Service (11/NW/0382). EXCEED received ethical approval from the Leicester Central Research Ethics Committee (13/EM/0226). Genes & Health received ethical approval from the NRES Committee London – South East (14/LO/1240). The activities of the EstBB are regulated by the Human Genes Research Act, which was adopted in 2000 specifically for the operations of EstBB. Individual level data analysis in EstBB was carried out under ethical approval 1.1-12/624 from the Estonian Committee on Bioethics and Human Research (Estonian Ministry of Social Affairs), using data according to release application 6-7/GI/2013 from the Estonian Biobank.

Note that full information on the approval of the study protocol must also be provided in the manuscript.

Field-specific reporting

Please select the one below that is the best fit for your research. If you are not sure, read the appropriate sections before making your selection.

☒ Life sciences ☐ Behavioural & social sciences ☐ Ecological, evolutionary & environmental sciences

For a reference copy of the document with all sections, see nature.com/documents/nr-reporting-summary-flat.pdf

Life sciences study design

All studies must disclose on these points even when the disclosure is negative.

Sample size

The analysis included readily available datasets which had both the relevant exposure and outcomes; with the largest sample size assembled to date for a genome-wide association study of TSH, and demonstrated appropriate power by identifying 158 previously unknown genetic associations. It is recognised that the number of samples from diverse ancestries is a limitation and further work is required in future to increase representation.

Data exclusions	Samples which did not meet genotyping quality control criteria were excluded
Replication	In addition to reaching P<5×10-8 in the Stage 1 meta-analysis, 230 of 249 sentinel variants available in Stage 2 reached P<5×10-8 after meta-analysing Stages 1 and 2 (N=343,604, Supplementary Table 2). Directions of effect and heterogeneity between Stage 1 studies are given in Supplementary Table 1.
Randomization	This was an observational study. Genetic variants are, in effect, randomized by the independent assortment of chromosomes during meiosis and are fixed at conception; thus, genetic association studies are not expected to be subject to confounding and reverse causation typically seen in non-genetic observational studies.
Blinding	Outcome data (TSH) were obtained from electronic health records collected prior to the conceptualisation of this analysis; thus, blinding at data collection was not needed. Blinding during analysis was not possible as all data were essential for analysis; in addition, in GWAS methods there is no hypothesis regarding the association between a given exposure (genetic variant) and outcome.

Reporting for specific materials, systems and methods

We require information from authors about some types of materials, experimental systems and methods used in many studies. Here, indicate whether each material, system or method listed is relevant to your study. If you are not sure if a list item applies to your research, read the appropriate section before selecting a response.

Materials & experimental systems

n/a	Involved in the study	
☒	☐	Antibodies
☒	☐	Eukaryotic cell lines
☒	☐	Palaeontology and archaeology
☒	☐	Animals and other organisms
☒	☐	Clinical data
☒	☐	Dual use research of concern

Methods

n/a	Involved in the study	
☒	☐	ChIP-seq
☒	☐	Flow cytometry
☒	☐	MRI-based neuroimaging
